# Supplementary material for: Climate change-induced shifts in the food systems and diet-related non-communicable diseases in sub-Saharan Africa: a scoping review and a conceptual framework
Source: BMJ Open. 2024 Jun 18;14(6):e080241. doi: 10.1136/bmjopen-2023-080241 (PMC11191816; doi:10.1136/bmjopen-2023-080241)
Supplement: Supplementary data [file bmjopen-2023-080241supp004.pdf]

**Supplementary Table 1**
**List of Sub-Saharan Africa countries**

| No | Country | Population (World Bank 2022) | GDP per capita (PPP) ($) (World Bank 2022) | World Bank 2023 Income Classification |
|---|---|---|---|---|
| 1 | Angola | 35,588,987 | 6,976.0 | Lower middle income |
| 2 | Benin | 13,352.86 | 4,057.5 | Lower middle income |
| 3 | Botswana | 2,384,246 | 18,329.8 | Upper middle income |
| 4 | Burkina Faso | 22,673,762 | 2,549.9 | Low income |
| 5 | Burundi | 12,889,576 | 836.5 | Low income |
| 6 | Cabo Verde | 593,149 | 8,716.0 | Lower middle income |
| 7 | Cameroon | 27,914,536 | 4,398.0 | Lower middle income |
| 8 | Central African Republic | 5,579,144 | 973.2 | Low income |
| 9 | Chad | 17,723,315 | 1,668.6 | Low income |
| 10 | Comoros | 836,774 | 3,833.7 | Lower middle income |
| 11 | Congo, Democratic Republic | 99,010,212 | 1,337.8 | Low income |
| 12 | Congo Republic | 5,970,424 | 4,335. | Lower middle income |
| 13 | Côte d'ivoire | 28,160,542 | 6,540.5 | Lower middle income |
| 14 | Equatorial Guinea | 1,674,908 | 17,620.7 | Upper middle income |
| 15 | Eritrea | 3,684,032 | 1,628.8 | Low income |
| 16 | Eswatini | 1,201,670 | 10,699.5 | Lower middle income |
| 17 | Ethiopia | 123,379,924 | 2,812.5 | Low income |
| 18 | Gabon | 2,388,992 | 16,465.0 | Upper middle income |
| 19 | The Gambia | 2,705,992 | 2,496.5 | Low income |
| 20 | Ghana | 33,475,870 | 6,473.1 | Lower middle income |
| 21 | Guinea | 13,859,341 | 3,188.1 | Lower middle income |
| 22 | Guinea-Bissau | 2,105,566 | 2,191.2 | Low income |
| 23 | Kenya | 54,027,487 | 5,765.8 | Lower middle income |
| 24 | Lesotho | 2,305,825 | 2,646.2 | Lower middle income |
| 25 | Liberia | 5,302,681 | 1,570.5 | Low income |
| 26 | Madagascar | 29,611,714 | 1,774.7 | Low income |
| 27 | Malawi | 20,405,317 | 1,732.6 | Low income |
| 28 | Mali | 22,593,590 | 2,518.9 | Low income |
| 29 | Mauritania | 4,736,139 | 6,295.8 | Lower middle income |
| 30 | Mauritius | 1,262,523 | 26,979.1 | Upper middle income |
| 31 | Mozambique | 32,969,518 | 1,477.3 | Low income |
| 32 | Namibia | 2,567,012 | 11,531.3 | Upper middle income |
| 33 | Niger | 26,207,977 | 1,505.5 | Low income |
| 34 | Nigeria | 218,541,212 | 5,862.2 | Lower middle income |
| 35 | Rwanda | 13,776,698 | 2,793.2 | Low income |
| 36 | Sao Tome and Principe | 227,38 | 4,061.8 | Lower middle income |
| 37 | Senegal | 17,316,449 | 4,210.4 | Lower middle income |
| 38 | Seychelles | 119,878 | 29,771.7 | High income |

| | | | | |
|---|---|---|---|---|
| 39 | Sierra Leone | 8,605,718 | 1,930.9 | Low income |
| 40 | Somalia | 17,597,511 | 1,711.0 | Low income |
| 41 | South Africa | 59,893,885 | 15,920.4 | Upper middle income |
| 42 | South Sudan | 10,913,164 | 1,181.9 | Low income |
| 43 | Sudan | 46,874,204 | 4,217.4 | Low income |
| 44 | Tanzania | 65,497,748 | 3,099.2 | Lower middle income |
| 45 | Togo | 8,848,699 | 2,601.8 | Low income |
| 46 | Uganda | 47,249,585 | 2,693.1 | Low income |
| 47 | Zambia | 20,017,675 | 3,975.6 | Lower middle income |
| 48 | Zimbabwe | 16,320,537 | 2,607.9 | Lower middle income |

Data retrieved from the World Bank website.
