# Supplementary material for: Climate change-induced shifts in the food systems and diet-related non-communicable diseases in sub-Saharan Africa: a scoping review and a conceptual framework
Source: BMJ Open. 2024 Jun 18;14(6):e080241. doi: 10.1136/bmjopen-2023-080241 (PMC11191816; doi:10.1136/bmjopen-2023-080241)
Supplement: Supplementary data [file bmjopen-2023-080241supp001.pdf]

Appendix 1

Search terms-Academic databases

"Sub-Saharan Africa" OR Angola OR Benin OR Botswana OR Burkina Faso OR Burundi OR "Cabo Verde" OR Cameroon OR "Central African Republic" OR Chad OR Comoros OR "Democratic Republic of Congo" OR "Republic of Congo" OR "Cote d'Ivoire" OR "Equatorial Guinea" OR Eritrea OR Eswatini OR Swaziland OR Ethiopia OR Gabon OR Gambia OR Ghana OR Guinea OR Guinea-Bissau OR Kenya OR Lesotho OR Liberia OR Madagascar OR Malawi OR Mali OR Mauritania OR Mauritius OR Mozambique OR Namibia OR Niger OR Nigeria OR Rwanda OR "Sao Tome and Principe" OR Senegal OR Seychelles OR "Sierra Leone" OR Somalia OR "South Africa" OR "South Sudan" OR Sudan OR Tanzania OR Togo OR Uganda OR Zambia OR Zimbabwe AND "Noncommunicable Diseases"[Mesh] OR "Noncommunicable Disease*" [tw] OR "Non-communicable Diseases" [tw] OR "Non communicable Diseases" [tw] OR "Non-communicable Chronic Diseases" [tw] OR "Non-communicable Chronic Disease*" [tw] OR "Non-infectious Diseases" [tw] OR "Chronic diseases" [tw] OR diabetes*[tw] OR DM* OR T2DM* OR overweight OR Hypertension*[tw] OR "high blood pressure"[tw] OR obesity*[tw] OR cholestrol* [tw] OR cancer*[tw] OR "Chronic respiratory diseases" AND "Climate Change"[Mesh] OR "Climate Changes*" [tw] OR "Climate change*" [tw] OR "climate impacts" OR "greenhouse gas" [tw] OR floods*[tw] OR drought*[tw] OR "heat waves*" [tw] OR greenhouse*[tw] OR "global warming"[tw] OR famine*[tw] OR temperature[tw] OR "extreme weather*"[tw] OR humidity OR precipitation OR "water-related"
