# Supplementary material for: Climate change-induced shifts in the food systems and diet-related non-communicable diseases in sub-Saharan Africa: a scoping review and a conceptual framework
Source: BMJ Open. 2024 Jun 18;14(6):e080241. doi: 10.1136/bmjopen-2023-080241 (PMC11191816; doi:10.1136/bmjopen-2023-080241)
Supplement: Supplementary data [file bmjopen-2023-080241supp002.pdf]

## Appendix 2

**Search terms-Grey literature**

**Google Scholar and Google**

Sub-Saharan Africa AND Noncommunicable Diseases OR Noncommunicable Disease OR Non-communicable Diseases OR Non communicable Diseases OR Non-communicable Chronic Diseases OR Non-communicable Chronic Disease OR Non-infectious Diseases OR Chronic diseases OR diabetes OR DM OR T2DM OR overweight OR Hypertension OR high blood pressure OR obesity OR cholesterol OR cancer OR Chronic respiratory diseases AND Climate Change OR Climate Changes OR Climate change OR climate impacts OR greenhouse gas OR floods OR drought OR heat waves OR greenhouse OR global warming OR famine OR temperature OR extreme weather OR humidity OR precipitation OR water-related

**FAO**

Climate change OR heat OR famine OR floods AND diabetes OR NCDs OR noncommunicable diseases OR Obesity OR cancer OR cardiovascular diseases AND Sub-Saharan Africa OR Republic of Congo OR Cote d'Ivoire OR Equatorial Guinea OR Eritrea OR Eswatini OR Swaziland OR Ethiopia OR Gabon OR Gambia OR Ghana OR Guinea OR Guinea-Bissau OR Kenya OR Lesotho OR Liberia OR Madagascar OR Malawi OR Mali OR Mauritania OR Mauritius OR Mozambique OR Namibia OR Sub-Saharan Africa OR Angola OR Benin OR Botswana OR Burkina Faso OR Burundi OR Cabo Verde OR Cameroon OR Central African Republic OR Chad OR Comoros OR Democratic Republic of Congo OR Niger OR Nigeria OR Rwanda OR Sao Tome and Principe OR Senegal OR Seychelles OR Sierra Leone OR Somalia OR South Africa OR South Sudan" OR Sudan OR Tanzania OR Togo OR Uganda OR Zambia OR Zimbabwe

**WHO, WFP and World Bank**

Climate change OR heat OR famine OR floods AND diabetes OR noncommunicable diseases OR NCDs OR Obesity OR cancer OR cardiovascular diseases AND Sub-Saharan

**IPFRI, CGIAR**

Search terms were not used in IPFRI and CGIAR websites, but hand searches were used to search studies under the publications and tools tab. This is because the search terms were bringing null results.

**Government websites**

Climate change OR heat OR famine OR floods AND diabetes OR NCDs OR noncommunicable diseases OR obesity OR cancer OR cardiovascular diseases AND Sub-Saharan Africa

No search limits used.
