# Supplementary material for: Climate change-induced shifts in the food systems and diet-related non-communicable diseases in sub-Saharan Africa: a scoping review and a conceptual framework
Source: BMJ Open. 2024 Jun 18;14(6):e080241. doi: 10.1136/bmjopen-2023-080241 (PMC11191816; doi:10.1136/bmjopen-2023-080241)
Supplement: Supplementary data [file bmjopen-2023-080241supp003.pdf]

## Appendix 3: Extraction sheet

| Author | Year | Study type | Aim of the study | Country of study | Climate change components discussed | Food systems pathway | Outcomes | Results on the effects of climate change on the outcomes | Causal or hypothetical association |
|---|---|---|---|---|---|---|---|---|---|
| | | | | | | | | | |
| | | | | | | | | | |
