# Supplementary material for: Climate change-induced shifts in the food systems and diet-related non-communicable diseases in sub-Saharan Africa: a scoping review and a conceptual framework
Source: BMJ Open. 2024 Jun 18;14(6):e080241. doi: 10.1136/bmjopen-2023-080241 (PMC11191816; doi:10.1136/bmjopen-2023-080241)
Supplement: Author's manuscript [file bmjopen-2023-080241.draft_revisions.pdf]

BMJ Open

BMJ Open is committed to open peer review. As part of this commitment we make the peer review history of every article we publish publicly available.

When an article is published we post the peer reviewers' comments and the authors' responses online. We also post the versions of the paper that were used during peer review. These are the versions that the peer review comments apply to.

The versions of the paper that follow are the versions that were submitted during the peer review process. They are not the versions of record or the final published versions. They should not be cited or distributed as the published version of this manuscript.

BMJ Open is an open access journal and the full, final, typeset and author-corrected version of record of the manuscript is available on our site with no access controls, subscription charges or pay-per-view fees (http://bmjopen.bmj.com).

If you have any questions on BMJ Open's open peer review process please email info.bmjopen@bmj.com

BMJ Open

Climate change-induced Shifts in Food Systems and Diet-related Noncommunicable Diseases in Sub-Saharan Africa: A Scoping Review and a Conceptual Framework

Journal:	*BMJ Open*
Manuscript ID	bmjopen-2023-080241
Article Type:	Original research
Date Submitted by the Author:	25-Sep-2023

Complete List of Authors:	Tapkigen, Janet; Tampere University, Department of Health Sciences<br>Harding, Seeromanie; King's College London<br>Jutta, Pulkki; Tampere University, Department of Health Sciences<br>Atkins, Salla; Tampere University, New Social Research and Faculty of Social Sciences; Karolinska Institute, Department of Public Health Sciences<br>Koivusalo, Meri; Tampere University, Faculty of Health Sciences

Keywords:	EPIDEMIOLOGY, Nutrition < TROPICAL MEDICINE, PUBLIC HEALTH

SCHOLARONE™
Manuscripts

I, the Submitting Author has the right to grant and does grant on behalf of all authors of the Work (as defined in the below author licence), an exclusive licence and/or a non-exclusive licence for contributions from authors who are: i) UK Crown employees; ii) where BMJ has agreed a CC-BY licence shall apply, and/or iii) in accordance with the terms applicable for US Federal Government officers or employees acting as part of their official duties; on a worldwide, perpetual, irrevocable, royalty-free basis to BMJ Publishing Group Ltd ("BMJ") its licensees and where the relevant Journal is co-owned by BMJ to the co-owners of the Journal, to publish the Work in this journal and any other BMJ products and to exploit all rights, as set out in our licence.

The Submitting Author accepts and understands that any supply made under these terms is made by BMJ to the Submitting Author unless you are acting as an employee on behalf of your employer or a postgraduate student of an affiliated institution which is paying any applicable article publishing charge ("APC") for Open Access articles. Where the Submitting Author wishes to make the Work available on an Open Access basis (and intends to pay the relevant APC), the terms of reuse of such Open Access shall be governed by a Creative Commons licence – details of these licences and which Creative Commons licence will apply to this Work are set out in our licence referred to above.

Other than as permitted in any relevant BMJ Author's Self Archiving Policies, I confirm this Work has not been accepted for publication elsewhere, is not being considered for publication elsewhere and does not duplicate material already published. I confirm all authors consent to publication of this Work and authorise the granting of this licence.

For peer review only

Climate change-induced Shifts in Food Systems and Diet-related Noncommunicable Diseases in Sub-Saharan Africa: A Scoping Review and a Conceptual Framework

Authors

Tapkigen Janet, MSc.
Affiliation
1. Department of Health Sciences, Faculty of Social Sciences, Tampere University, Finland, Kalevantie 4, 33100 Tampere.

Harding Seeromanie, Ph.D.
Affiliation
2. Department of Population Health Sciences, School of Life Course & Population Sciences, King's College London, London SE1 1UL, UK.

Pulkki Jutta, Ph.D.
Affiliation
3. Department of Health Sciences, Faculty of Social Sciences, Tampere University, Finland, Kalevantie 4, 33100 Tampere.

Atkins Salla, Ph.D.
Affiliations
1. Department of Health Sciences, Faculty of Social Sciences, Tampere University, Finland, Kalevantie 4, 33100 Tampere.
2. Department of Global Public Health, Karolinska Institutet, Stockholm, Sweden.
3. WHO Collaborating Centre on Tuberculosis and Social Medicine, Department of Global Public Health, Karolinska Institutet, Stockholm, Sweden.

Koivusalo Meri, Ph.D.
Affiliations
1. Department of Health Sciences, Faculty of Social Sciences, Tampere University, Finland, Kalevantie 4, 33100 Tampere.
2. Centre for Health in All Policies and Social Determinants of Health, Tampere University, Finland.

Corresponding Author

Janet Tapkigen, MSc.
Faculty of Social Sciences,
Tampere University, Finland, Kalevantie 4, 33100 Tampere
Email: janet.tapkigen@tuni.fi
Tel : +358 (0) 451378262

For peer review only - http://bmjopen.bmj.com/site/about/guidelines.xhtml

Summary

Background: Sub-Saharan Africa (SSA) is facing food system challenges: climate change, food insecurity, and diet-related noncommunicable diseases (DR-NCDs). Climate change is threatening the region's ecosystems, agricultural productivity, and food security, exacerbating existing vulnerabilities. Concurrently, there is a growing burden of DR-NCDs, largely driven by unhealthy dietary patterns resulting from changes in food systems. While the High-Level Panel of Experts has provided broad-scale evidence of environmental influence on food systems and DR-NCDs, the linkages in SSA are unclear. Thus, this review aims to summarise the evidence in the literature on these interconnections while proposing a conceptual framework for SSA.

Methods: A scoping review following Johanna Briggs Institute guidelines was conducted by searching eleven databases. We developed a conceptual framework and searched for evidence in SSA to support or disprove it. A narrative approach was used to analyse the data.

Findings: The search yielded 18,525 studies, with ten included in the review. Most studies used a cross-sectional design (n=7). Five explored the influence of humidity on liver cancer through food storage. Four explored the influence of rainfall and temperature on diabetes and obesity through food production. Evidence from cross-sectional studies showed that high humidity is associated with liver cancer and rainfall with diabetes.

Interpretation: The review highlights knowledge gaps on the linkages between climate change and systemic risks, food systems, and DR-NCDs in SSA. It offers a conceptual framework that can serve as a guide for developing interventions aimed at climate change and DR-NCDs.

Funding: Authors were supported by their respective institutions.

For peer review only - http://bmjopen.bmj.com/site/about/guidelines.xhtml

Key messages

Evidence before this study

Existing frameworks have explored the broad-scale relationships and pathways between climate change and the risks associated with nutrition outcomes. The High level Panel of Experts (HLPE), for instance, has developed a food systems framework illustrating the interplay between diverse drivers within food systems, including climate change, and their effects on health and nutrition outcomes.

Added value of this study

This study is novel as it introduces a distinctive perspective by offering a focused and region-specific insight into how the scientific literature addresses the issue of climate change and DR-NCDs within SSA. The conceptual framework developed in this review for SSA, tailored to the role of food systems in nutrition and DR-NCDs, is designed to facilitate a comprehensive grasp of the specific pathways and driving forces within food systems that contribute to DR-NCDs.

Implications of all available evidence

The HLPE framework and our own conceptual framework share similarities in illustrating the pathways through which climate change impacts food systems and, subsequently on DR-NCDs in SSA. The HLPE framework highlights food production, storage, distribution, processing, and packaging, as well as retail and marketing, as key pathways for the influence of climate change on DR-NCDs. On the other hand, our study specifically identifies food production, storage, and consumption as the primary pathways within SSA. Notably, our research underscores the potential impact of humidity and rainfall on food storage as a crucial pathway influencing DR-NCDs within the region.

For peer review only

Introduction

Noncommunicable diseases (NCDs) account for 74% (41 million) of all deaths globally every year [1]. The primary causes of NCD-related deaths are cardiovascular diseases (accounting for 17.9 million deaths annually), cancer (9.3 million), chronic respiratory diseases (4.1 million), and diabetes (2.0 million) [1]. Collectively, these diseases contribute to 80% of all NCD-related deaths [1]. In Sub-Saharan Africa (SSA), cardiovascular diseases are the leading cause of NCD-related deaths, accounting for 15.1% of the NCD burden. Diabetes and liver cancer also make significant contributions to the NCD burden in the region, accounting for 4.2% and 0.8% of NCD-related deaths, respectively [2]. These three diseases – cardiovascular diseases, liver cancer, and diabetes – are known as diet-related NCDs (DR-NCDs), as they are largely caused by poor dietary habits, characterised by highly processed foods high in saturated fats, sugars, and salt [3].

To effectively address the burden of DR-NCDs, it is essential to understand the dynamics of food systems and their role in shaping diets. Food systems can be described as intricate and interconnected networks comprising the environment, people, processes, infrastructure, and institutions involved in various aspects of food production, processing, transportation, storage, distribution, retailing, and consumption [4]. The outcomes of these activities, as well as their governance, collectively define the functioning of the food system [4]. The interconnected components of food systems, namely the food supply chain, food environments, and consumer behaviour, are illustrated in Figure 1.

The food supply chains encompass all stages from production to waste disposal [5], and they depend on ecological, human, energy, and economic systems to produce and distribute foods [6]. On the other hand, food environments encompass the settings where consumers make food-related choices [5], thus connecting food supply and demand [6]. Food environments, therefore, overlap with food supply chains, consumer behaviour, and diets [6] and are divided into personal environments (accessibility, affordability, convenience, desirability) and external environments (food vendors, prices, income levels, knowledge, sociocultural norms, marketing practices, regulations) [7]. For certain individuals, these environments are local (involving self-produced or locally sourced foods), while for others, they are global (encompassing foods from local, regional, and international sources) [5]. Food environments influence consumer behaviour [6], which refers to decisions and actions individuals take when acquiring and consuming food [4]. These decisions shape diets in terms of quantity, quality, diversity, safety, and adequacy of food [6]. Consequently, diets exert influence on interconnected systems, including nutrition outcomes that directly impact healthcare systems, as well as the climate impacts of diets that affect ecosystems [6].

Climate change, driven by human activities, has been widely acknowledged as a significant contributor to the development and exacerbation of DR-NCDs [8]. The rising global temperatures, erratic rainfall patterns, and increasing frequency of extreme weather events significantly influence agricultural and food systems [9]. These climatic shifts impact the quality, availability, and affordability of food [9 10], potentially altering food access and choices, with

For peer review only - http://bmjopen.bmj.com/site/about/guidelines.xhtml

implications for nutrition and dietary habits [10]. As food systems are undergoing profound transformations driven by population growth, globalisation, technology, urbanisation, trade liberalisation, migration, policies, competition for essential resources (water, energy, land), and shifts in income distribution, the impacts of climate change compound the effects of these drivers [4]. As such, climate change can be seen as amplifying the underlying drivers associated with DR-NCDs [8,9].

To effectively address the burden of DR-NCDs in SSA amid these climate-induced changes in the food systems, a shift from conventional disease-specific approaches [11] to a more holistic and systems-oriented approach is required [12]. Traditional interventions for NCDs in SSA have often narrowly focused on one sector [13], overlooking the interconnected nature of the other drivers influencing NCDs [14]. However, adopting a food systems approach allows for a holistic and comprehensive view of the entire food system, recognising the intricate interplay and feedback loops between different risk factors for NCDs [13]. This approach employs food systems thinking, which acknowledges the interdependence of drivers and relationships within the food systems [13].

In the context of addressing NCDs, adopting a systems thinking approach acknowledges that one system alone, for example, the healthcare system, cannot solely determine health outcomes [15]. Instead, it recognises the significant role played by interconnected systems (such as the environment, energy, and government sectors) in shaping environments that impact DR-NCDs [15]. However, implementing a food systems approach in SSA comes with challenges resulting from limited evidence on the specific relationships between climate change and related systemic risks, food systems, and DR-NCDs in the region. While previous reviews and food systems frameworks have provided valuable broad-scale perspectives and frameworks [8,16], there is an urgent need to generate more localised and region-specific evidence and frameworks. The present framework will serve as a crucial guide for future interventions, research, and preventive measures tailored to SSA.

Aims of the review

This review aims to summarise the evidence on the relationships between climate change, food systems, and DR-NCDs in SSA and develop a conceptual framework for food systems and DR-NCDS and nutrition outcomes for SSA.

For peer review only - http://bmjopen.bmj.com/site/about/guidelines.xhtml

Methods

Conceptual framework development

The development of the conceptual framework (Figure 1) involved a comprehensive process aimed at capturing the broad understanding and interplay between climate change and related systemic risks, food systems, and their impact on nutrition outcomes.

In the framework, climate change-related systemic risks were defined as impacts of climate change on other interconnected sectors or systems that subsequently exert an influence on food systems.

The framework presented in Figure 1 was developed in November 2022. It draws insights from and builds upon the foundational concepts initially presented by the HLPE framework [4]. Additionally, our framework is enriched by insights extracted from scientific literature [17–22], which delves into the intricate connections linking climate change, food systems, and nutritional outcomes. Based on the knowledge drawn from these sources, pathways in which climate change may affect nutrition outcomes and DR-NCDs were developed. These pathways include food (1) production, (2) storage, (3) distribution, (4) processing and packaging, (5) retail and marketing, as well as (6) consumption.

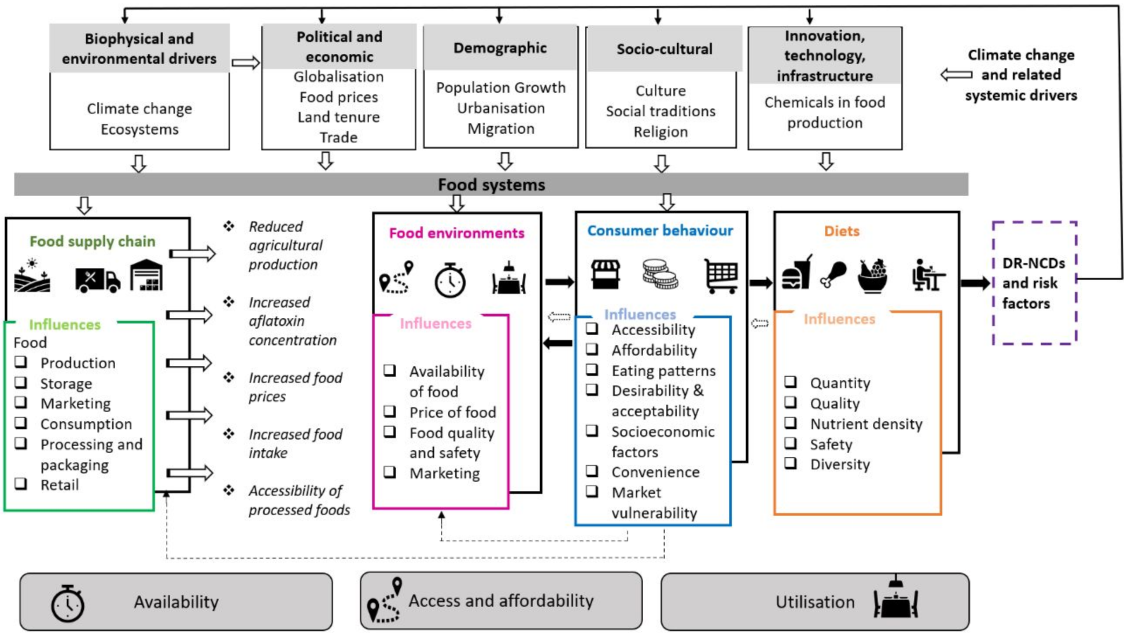

Figure 1: Food Systems Conceptual Framework for Nutrition and DR-NCDs, adapted from the HLPE framework [4]

Regarding the *food production pathway,* we hypothesise that the effects of climate change could reduce crop production and yields, consequently reducing food availability. This decline in food availability may trigger increases in food prices, posing a threat to food security and dietary diversity, particularly in terms of limited fruits and vegetables. This, in turn, could impact health outcomes [18].

For peer review only - http://bmjopen.bmj.com/site/about/guidelines.xhtml

Under the *food storage pathway,* we propose that reliance on stored crops vulnerable to the impacts of extreme rainfall events can have adverse effects on both the quantity and quality of food. Wet conditions can create a conducive environment for the emergence of infectious pathogens. These pathogens, affecting crops during harvesting and storage, could lead to the production of carcinogenic aflatoxins produced by certain moulds. The presence of aflatoxins in food has been associated with hepatocellular carcinoma, a primary contributor to the incidence of liver cancer [19].

Within the contexts of *food distribution and market pathways* and *food processing and packaging pathways*, we put forth the theory that extreme weather events, such as floods, might disrupt distribution networks, infrastructure, and trade channels, resulting in food losses – especially perishable goods [20,21]. These disruptions can impact the affordability and diversity of food items, impacting food and nutrition security. When comparing fresh perishable foods to processed alternatives, it becomes increasingly clear that processed foods tend to withstand environmental impacts more effectively, making them a preferable choice within the realm of climate variability, albeit with potential trade-offs concerning nutrition value and health implications [20,21,23]. Moreover, decreased food availability can subsequently drive up food prices, potentially creating a scenario where imported goods become more easily accessible. Often, these imported products lean towards cost-effectiveness and a high degree of processing.

In the *food consumption pathway*, we posit that climate variability may influence consumption patterns by affecting availability, affordability, and acceptability [24]. These shifts could influence food quantity, quality, and prices as well as reduced income [24]. These combined effects can have multifaceted implications for nutritional access, availability, and affordability. This can lead to shifts in dietary preferences, often towards more economical yet highly processed food options [22].

Scoping review method

This scoping review followed the methodological guidance for conducting a JBI scoping review [25]. The method includes: 1) identifying the research question; 2) inclusion criteria; 3) concept; 4) context; 5) types of evidence sources; 6) search strategy; 7) evidence screening and selection; 8) data extraction; 9) data analysis; and 10) presentation of results.

Scoping reviews are helpful when the literature is complex and serve as a tool to provide policymakers with a comprehensive understanding of the nature of the concept and how that concept has been studied within the existing body of literature [25]. This review was reported per the Preferred Reporting Items for Systematic Reviews and Meta-analysis (PRISMA) reporting guidelines [26]. This study was based on a registered protocol (https://osf.io/9unbd).

Research question

How are changes in food systems affected by climate change and related systemic risks, and how do these changes influence nutrition outcomes and DR-NCDs in SSA?

Inclusion criteria

To be eligible, studies had to

For peer review only - http://bmjopen.bmj.com/site/about/guidelines.xhtml

(1) Investigate the relationship between climate change and related systemic risks, food systems, and DR-NCDs and their risk factors;

(2) Be available as full texts;

(3) Be written in any language;

(4) Be related to/conducted in SSA;

We excluded

(1) Studies that looked at the association between climate change and DR-NCDs through pathways that do not include food systems, such as social inequalities;

(2) Articles not available as full texts;

(3) Studies not conducted in SSA;

(4) Editorials, letters to the editor, or theoretical discussions of potential impacts or planned programmes that have not yet been implemented, theses, newspapers, magazines, conference abstracts, podcasts, radio programmes, and blogs.

Concept

This review primarily focused on investigating the relationship between climate change, along with interconnected systemic factors (i.e., urbanisation, globalisation, food prices), on food systems and subsequently on DR-NCDs.

Context

The scope of this review was centred on examining studies carried out specifically in SSA.

Data sources

An initial search was conducted in January 2022 in ProQuest-9 databases (ProQuest Central, Health and Medical Collection, Environment Science Database, Public Health Collection, Research Library: Health and Medicine, Middle East and Africa Database, Social Science Database, Social Science Premium Collection, and Agriculture Science Database), and PubMed. Another search was conducted in December 2022. This was complimented by searching grey literature, including Google Scholar (first 20 pages), hand searches, and checking international websites (International Food Policy Research Institute, Consultative Group on International Agricultural Research, World Health Organization, Food and Agriculture Organization, World Food Programme, and World Bank) in July 2023.

Search strategy

A review of PubMed enabled the identification of key terms for the search strategy. The search terms used were related to (i) climate change*, (ii) SSA countries, (iii) DR-NCDs*, and (iv) nutrition outcomes*. The identified search terms were adapted for other databases. The detailed search strategy is available in Appendix 1. No language restriction was applied. The search started from 1995, when the Global Framework for Climate Change was instituted to address

For peer review only - http://bmjopen.bmj.com/site/about/guidelines.xhtml

the challenges posed by global warming [27,28]. We included studies published in peer-reviewed journals between 1995 and 2022.

Evidence screening and selection

Following the search, all identified citations were collated and uploaded into Mendeley, where duplicates were removed. The studies were then uploaded to Rayyan.ai [29] for the title and abstract screening. Two authors, JT and MK, independently screened the titles and abstracts and extracted data from studies that met the inclusion criteria. Relevant studies were retrieved in full and assessed individually by two reviewers (JT and MK). Disagreements were resolved through consensus between the two reviewers. Ineligible studies were excluded.

Data extraction

JT extracted quantitative and qualitative data in Excel using an extraction form developed for the present study (Appendix 2). The data included the following characteristics: author, study type, country of study, climate change component discussed, DR-NCD outcomes and risk factors, and impacts of climate change on DR-NCDs.

Data analysis

A narrative synthesis approach [30] was applied in this review. This involved integration of quantitative, mixed-methods, and qualitative studies through collation, analysis, synthesis, and presentation [30].

Presentation of results

The results are presented under three main sections: 1) characteristics of included studies; 2) a conceptual framework for food systems and DR-NCDs in SSA; and 3) influence of climate change on DR-NCDs. The results are presented according to the food system pathways identified.

Role of the funding source

Authors were supported by their respective institutions.

Risk assessment

No risk assessment was conducted.

For peer review only

Results

The search process identified 18,525 studies after removing 1,221 duplicates. Among these, 7,286 studies were peer-reviewed articles, and 11,239 were grey literature. For peer-reviewed articles, we screened both the titles and abstracts. As for the grey literature, we first screened the titles, and if they were unclear, we also screened the abstracts. We excluded 18,428 records based on irrelevant titles and abstracts, leaving us with 106 studies for which we retrieved the full texts for detailed assessment. Of these, ten studies met the eligibility criteria and were included in this review. The search process is depicted in Figure 2.

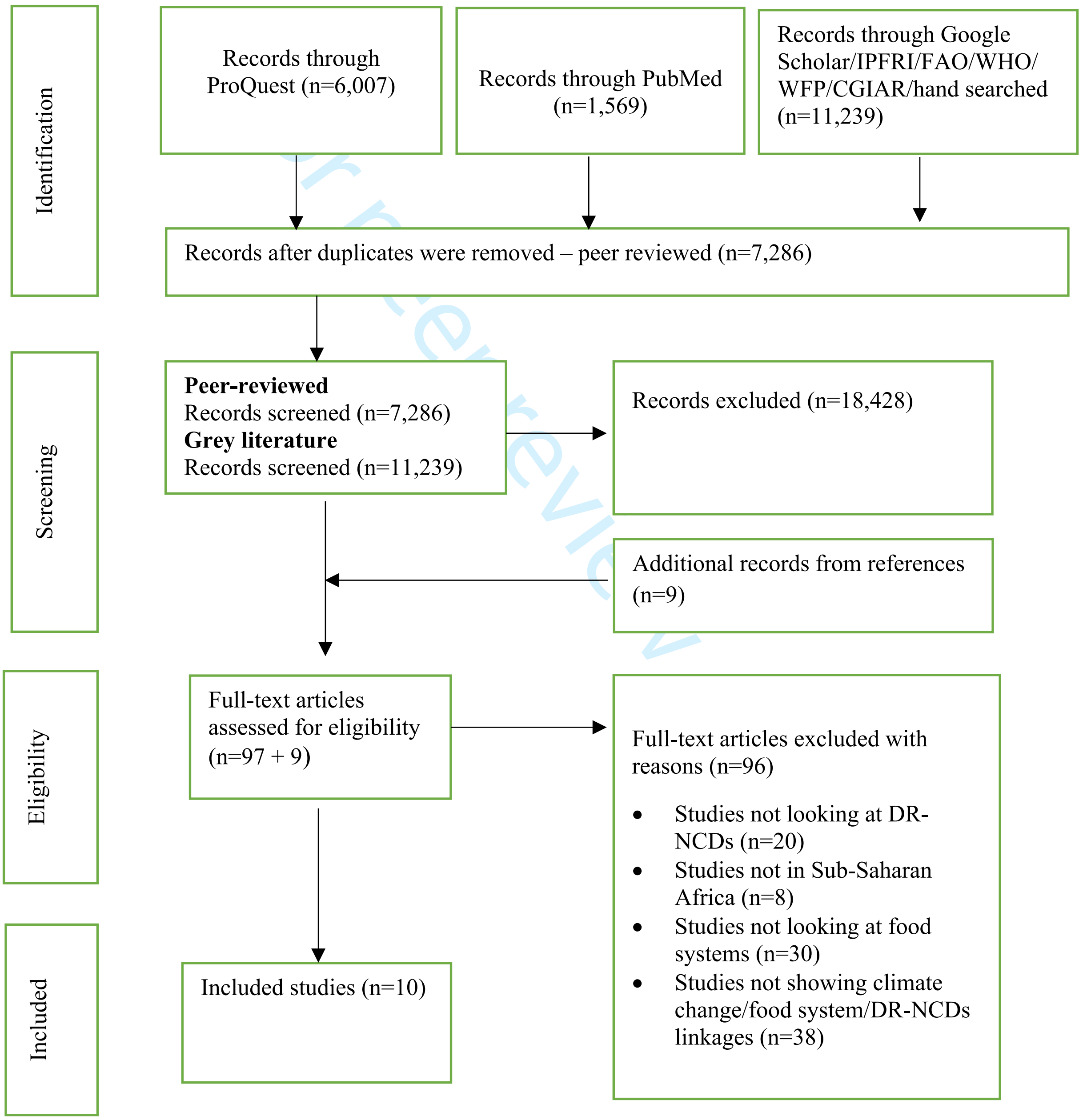

Figure 2: PRISMA framework

For peer review only - http://bmjopen.bmj.com/site/about/guidelines.xhtml

Quality appraisal

No quality appraisal was conducted.

Characteristics of included studies

Ten studies met our inclusion criteria [31–40], including eight quantitative [31,32,34–37,39,40] and two qualitative studies. Seven studies were published in the 2020s [33–39], two in the 2010s [32,40] and one in the 1990s [31]. Three studies were conducted in Kenya [35,38,40], two in Ghana [33,39], with one study each in Cameroon [32], Malawi [34], Sudan [31], Tanzania [37], and Zimbabwe [36], as shown in Table 1.

Table 1: Overview of included studies

Author	Year	Country	Study design	Aspect of climate change	DR-NCDs and risk factors
Omer [31]	1998	Sudan	Cross-sectional survey	Humidity	Liver cancer
Lontchi-Yimagou [32]	2016	Cameroon	Retrospective study	Temperature and rainfall	Diabetes
Sirma [40]	2019	Kenya	Cross-sectional survey	Precipitation, humidity, floods, droughts, temperature	Liver cancer
Odonkor & Sallar [33]	2020	Ghana	Descriptive cross-sectional design	Global warming	Heart disease
Ngwira 2020 [34]	2020	Malawi	Cross-sectional analysis	Rainfall	Overweight
Kasomo [35]	2021	Kenya	Cross-sectional analysis	Temperature and rainfall	Overweight, obesity
Akello [36]	2021	Zimbabwe	Cross-sectional analysis	Humidity	Liver cancer
Kimanya [37]	2021	Tanzania	Cross-sectional survey	Humidity and temperature	Liver cancer
Andersen [38]	2021	Kenya	Qualitative	Climate change	Diabetes, hypertension, heart attack, cancer, and obesity
Kortei [39]	2022	Ghana	Cross-sectional survey	Humidity	Liver cancer

Overall study design

Among the studies included, four [32,36,39,40] studies used primary data collection methods to estimate DR-NCDs and nutrition outcomes. Three studies [34,35,37] drew upon data from national health sources to recruit participants. All three studies [34,35,37], utilising secondary data, relied on original survey data to measure and estimate health outcomes.

Three studies [36,39,40] used AF and FM levels from grain and milk samples to quantify dietary exposure and estimate the risk of liver cancer. Meanwhile, five studies [31,32,34,35,37] used human samples to assess dietary exposure and DR-NCD risk. Notably, two studies [33,38] took an alternate approach, looking at the perceptions of participants to illustrate health risks associated with climate change.

Study types included (n=8) cross-sectional surveys [31,32,34–39], qualitative methods [38] and a descriptive cross-sectional study [33]. The sample size ranged from 28 to 40,300.

Climate change exposure

For peer review only - http://bmjopen.bmj.com/site/about/guidelines.xhtml

The most common climate variable investigated was humidity [31,36,37,39,40], in 5 out of 10 studies (50%). Four studies investigated DR-NCD outcomes following variation in rainfall [32,34,35,40], and temperature [32,35,37,40].

DR-NCD and Nutrition outcomes

Five cross-sectional studies measured [31,37] and estimate [36,39,40] liver cancer rates. Two issues looked at the relationship between climate vents and diabetes [32,38]. Four studies looked at overweight [34,35], obesity [35], heart disease [32,38], and hypertension [38].

Conceptual framework of food systems for DR-NCDs and nutrition with evidence drawn from the local context

This conceptual framework (Figure 3) is based on the findings from our review. The evidence from SSA aligns with three of the five pathways (production, storage, and consumption) highlighted in the framework. The pathways shaded in grey are contextual and were not supported by evidence from this review.

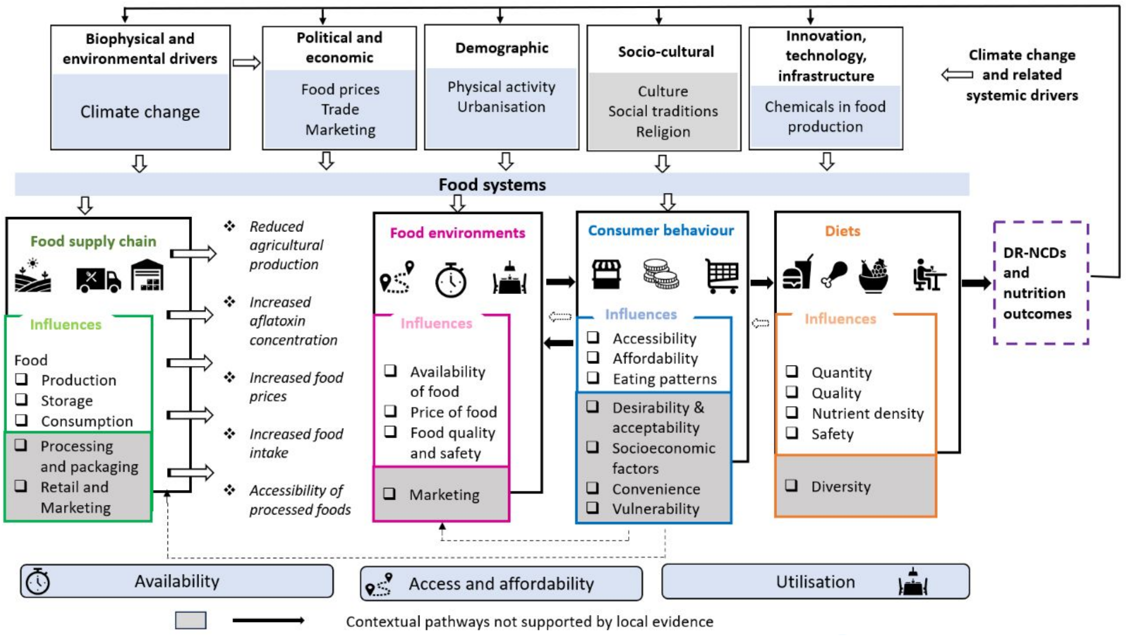

Figure 3: Conceptual framework of food systems for DR-NCDs and nutrition in Sub-Saharan Africa

Food system pathways through which climate change influences nutrition outcomes and DR-NCDs in SSA

Table 2 below shows the preliminary results on how climate change and systemic risks influence DR-NCDs and is organised based on the food system pathways identified. Liver cancer was the most frequently reported DR-NCD.

For peer review only - http://bmjopen.bmj.com/site/about/guidelines.xhtml

Table 2: Description of studies included in the review by food system pathway.

Author/ year	Country	Study design	Method of analysis	Study objectives	Sample size	Climate change component	Outcome	Key findings
**Food production pathway**								
Andersen 2021 [38]	Kenya	Qualitative	Descriptive Statistics	To explore knowledge and perspectives on climate change and health-related issues, with a particular focus on noncommunicable diseases	28	Climate change	Diabetes, hypertension, heart attack, lung disease, cancer, and obesity	Climate change-induced food insecurity and rising food prices emerged as prominent challenges.
Kasomo 2021 [35]	Kenya	Cross-sectional analysis	Inferential Statistics	To quantify spatial variations and estimate the effect of climatic and environmental factors on under- and over-nutrition among women in Kenya	40,300	Temperature and precipitation	Obesity and overweight	Higher precipitation levels were associated with an increased likelihood of women being overweight and obese.
Ngwira 2020 [34]	Malawi	Cross-sectional analysis	Multivariate probit model	To investigate the effects of climate and location using the multivariate model of malnutrition	5,149	Rainfall	Overweight	A positive correlation was observed between rainfall and overweight individuals.
Odonkor & Sallar 2020 [33]	Ghana	Descriptive cross-sectional design	Inferential Statistics	To investigate public knowledge of global warming and its effects on human health	1,130	Global warming	Heart disease	Rising food prices impact the affordability and availability of food in urban areas.
**Food storage pathway**								

For peer review only

Akello 2021 [36]	Zimbabwe	Cross-sectional analysis	Inferential Statistics	To assess frequencies of mycotoxin-producing fungi in maize, sorghum, pearl millet, and finger millet cropped and traded in Zimbabwe	NI[1]	Humidity	Liver cancer	Consumption of aflatoxin-contaminated maize stored for 4-6 months increased liver cancer rates.
Kimanya 2021 [37]	Tanzania	Cross-sectional analysis	Inferential Statistics	To estimate the population risk of aflatoxin-induced liver cancer	NI[1]	Temperature and humidity	Liver cancer	Population risk for aflatoxin-induced liver cancer nationwide was estimated to be 2.95 cases per 100,000 people.
Kortei 2022 [39]	Ghana	Cross-sectional survey	NI	To evaluate AFM_1 levels and cancer risks associated with *burkina* (a millet-based fermented milk product)	150	Temperature and humidity	Liver cancer	Consumption of *burkina* showed varied risk levels of liver cancer.
Omer 1998 [31]	Sudan	Cross-sectional survey	Inferential Statistics	To investigate whether aflatoxin contamination of peanut products may contribute to the incidence of hepatocellular carcinoma (HCC) in Sudan	54	Temperature and humidity	Liver cancer	Aflatoxin contamination of peanuts has a significant association with liver cancer.
Sirma 2019 [40]	Kenya	Cross-sectional survey	Inferential Statistics	To assess the risk of liver cancer posed by AFM_1 in milk, assuming 10-fold lower carcinogenicity than AFB_1	NI[1]	Rainfall, droughts, humidity, temperature, floods	Liver cancer	Aflatoxin exposure from milk contributed a small percentage to liver cancer incidence rates.
**Food consumption pathway**								
Lontchi-Yimagou 2016 [32]	Cameroon	Retrospective study	Inferential Statistics	To investigate the relationship between diabetes hospitalisation rates and climate variations in Yaoundé	818	Temperature and precipitation	Diabetes	Diabetes admissions were higher during rainy seasons due to nutritional variability.

[1] NI-Not indicated

Food storage pathway

Five studies [31,36,37,39,40] examined how humidity and precipitation correlate with liver cancer risks through food storage practices. These investigations showed that the risk of liver cancer increased after exposure to Aflatoxins (AF) from certain foods. AF contamination was reported in maize [36,37], small grains (finger millet, pearl millet, sorghum) [36], and in milk [40]. Additionally, Aflatoxin B_1 (AFB_1) contamination was found in peanuts [31], while Aflatoxin M_1 (AFM_1) contamination was detected in *burkina*, a millet-based fermented milk beverage [39].

In various food sources, AF contamination levels surpassed recommended limits (>5 µg/kg for AFBI or >50 ng/kg for AFM1), observed in maize [36], millet-based fermented milk beverage (*burkina*) [39], commercial animal feeds [40], milk [40], and peanuts [31]. However, pearl millet, finger millet, and sorghum had lower AF levels [36]. While most studies analysed AF levels in food samples, one study examined blood samples from children consuming maize, revealing high AF exposure levels [37].

Risk of AF contamination was notably elevated in drier, hotter regions, while higher fumonisin (FM)- contamination occurred during wetter years [36]. Two studies explored AFM_1 concentration and storage conditions, linking peanut products stored in humid environments, particularly mud buildings, to higher AFM_1 concentrations (above 10 ug/kg)[31]. Another study demonstrated that storing aflatoxin-contaminated maize for 4–6 months increased AFM_1 concentrations [36].

Risk assessment based on AF exposure indicated that the consumption of small grains posed low liver cancer risks [31,36] as did milk consumption [40]. In contrast, consuming maize with AF contamination [36] as well as fermented milk (*burkina*) [39] presented high liver cancer risks, although one study reported low liver cancer risks associated with corn consumption [31].

In Zimbabwe, consuming aflatoxin-contaminated maize stored for 4–6 months increased liver cancer rates from 0.4 to 2.1 cases per year/100,000 population [36]. Another study showed an increased risk of liver cancer among infants, toddlers, children, and adolescents consuming *burkina*, while the adult population faced a lower risk [39]. In the case of peanuts, the results showed an increased risk of liver cancer associated with peanut consumption [31], with higher liver cancer rates among those consuming peanuts from humid storage (mud buildings) than from dry storage conditions [31]. In Tanzania, aflatoxin-induced liver cancer from contaminated maize was reported with the AF in crops attributed to drought [37].

The risk of developing liver cancer from the consumption of small grains and milk was quite low (≤0.02 cases per 100,000 individuals annually [36]). Another study in Kenya revealed that the presence of AFM_1 from milk consumption plays a negligible role in the overall annual incidence rate of liver cancer [40].

Food production pathway

Four studies examined the relationships between precipitation, temperature, and various health outcomes, including obesity [35], overweight [34,35], heart disease [33], and DR-NCDs in general [38].

For peer review only - http://bmjopen.bmj.com/site/about/guidelines.xhtml

The association between precipitation patterns and food production was highlighted in two studies [35,38], indicating a reduction in food production due to variable rainfall. This phenomenon led to water scarcity, destruction of nutritious crops, and, subsequently, increased reliance on non-nutritious foods, contributing to obesity [35]. Another study indicated that harsh weather patterns disrupted agricultural production, resulting in higher costs of food in urban areas due to reliance on imported produce [38]. Additionally, climate change-driven reliance on chemically treated food production raised concerns about food quality [38]

Interestingly, one study suggested that rainfall variability could increase food production, potentially contributing to overweight conditions due to increased food availability fostering overeating behaviours [34].

The association of global warming with food production and heart disease was assessed in Ghana. Respondents' opinions revealed a mix of awareness levels regarding the link between global warming and heart disease [33].

Food consumption pathway

One study looked at how rainfall and temperature influenced food consumption by impacting food quality, resulting in diabetes [32]. This retrospective study revealed that diabetes admissions were higher during rainy seasons compared to dry seasons [32].

For peer review only - http://bmjopen.bmj.com/site/about/guidelines.xhtml

Discussion

The review indicates that climate change-related fluctuations, including fluctuations in rainfall and temperature and extreme weather events such as floods and droughts, can disrupt agricultural productivity. This leads to low crop yields, scarcity of food, and increased food costs. These factors contribute to a shift towards less nutritious and more processed foods associated with the development of DR-NCDs. The findings from this review were aligned with the HLPE framework [4].

Food storage: This pathway focuses on how climate-induced changes in humidity and precipitation affect food harvest and storage conditions, leading to the growth of Aflatoxin B_1 and Aflatoxin M_1 (AFM_1) flat – a type of mycotoxin produced by fungi *Aspergillus flavus* and *Aspergillus parasiticus* – present in grains, maize, nuts, peanuts, flour, and milk [41]. In Ghana, Kenya, Sudan, Tanzania, and Zimbabwe, consumption of aflatoxin-contaminated foods was linked to an increased risk of liver cancer. Overall, liver cancer was the most reported form of DR-NCD. Consuming aflatoxin-contaminated maize stored for 4–6 months was linked to higher lung cancer rates in Zimbabwe. Additionally, local storage conditions, such as using mud buildings in humid regions, were significantly associated with liver cancer in Sudan. The review's findings are consistent with other research conducted in Bangladesh, which reported a significant portion of liver cancer cases attributed to dietary aflatoxin exposure from maize and other crops in warm and humid conditions [42]. Notably, based on the findings of this review, the risk of aflatoxin contamination and its associated liver cancer risk was found to present a lower risk in milk and other grains. This observation is in line with studies by Turna et al. [43] and a study in Bangladesh [42], where aflatoxin exposure in milk and grains such as wheat and rice was not associated with liver cancer. Several factors are identified as influencing aflatoxin contamination, including storage conditions, duration, and type of crop storage structure. Aflatoxins are prone to develop during food production in dry and humid conditions, whereas wet and humid conditions during harvesting and storage create favourable conditions for the growth of aflatoxins [44].

Food production: Evidence from Cameroon, Kenya, and Malawi showed how climate variables correlate with diabetes, heart disease, and obesity. Seasonal changes in rainfall were found to influence physical activity and dietary choices, and in turn diabetes. Moreover, increased precipitation leading to food surplus was associated with overeating and obesity in the region. The review's findings align with other studies that have linked disruptions in agricultural production resulting from extreme weather events with diabetes and obesity [45]. During rainy seasons and extreme weather events, agricultural production activities are disrupted, leading to food shortages [39]. During food shortages, individuals with diabetes may be forced to rely on unhealthy processed foods, worsening their condition [39].

Food consumption: The increased access to and consumption of processed and unhealthy foods may contribute to the burden of DR-NCDs. This pathway is supported by a review [23] highlighting that extreme weather events are likely to become more frequent determinants of food purchase and consumption. These events can have a dual impact, either by restricting consumers' access to food or by shaping their food preferences based on the availability of certain product [23].

For peer review only - http://bmjopen.bmj.com/site/about/guidelines.xhtml

Conceptual framework

Most pathways in the conceptual framework seemed to be supported by evidence in SSA. However, the studies included in the review were too few to distinguish or support all the other pathways.

Strengths

This review exhibits several notable strengths.

Firstly, the review aligns with our framework (Figure 1), providing a solid foundation for assessing the impact of climate change on food systems in SSA. By following this established framework, the review ensures consistency and comparability with existing global knowledge on the subject.

Moreover, the review goes beyond existing frameworks by developing a novel conceptual framework tailored to SSA. This customised framework highlights how climate change and related systemic drivers influence SSA's food systems and contribute to the development of DR-NCDs. This approach enhances the framework's applicability in guiding targeted interventions such as better crop storage, sustainable agriculture, and policies that address the region's specific concerns.

The review also adheres to a rigorous and systematic approach, following JBI guidance for conducting scoping reviews. This systematic process ensures the reliability and validity of the findings.

Limitations

One key limitation of the review is the possibility of publication bias, as the review only focused on international organisations and academic publications. Relevant studies published in country-based databases as well as local languages, might have been missed, potentially impacting the comprehensiveness of the evidence.

Additionally, the nature of the studies included in the review – mainly observational and descriptive – limits the establishment of definitive causal relationships between climate change, food systems, and DR-NCDs. Although they offered insights and generated hypotheses of the potential linkages, they may not provide conclusive evidence on causality. Further research, including longitudinal and time series analysis studies, would be necessary to establish more robust causal links.

Furthermore, this review did not explore the potential effects of foetal famine on DR-NCDs later in life, which represents another area that could be relevant to the overall understanding of climate change impacts on DR-NCDs in SSA. Including such evidence in future research could contribute to a more comprehensive analysis of the factors influencing DR-NCDs in the region.

Conclusion

This comprehensive review underscores the urgent need to address the knowledge gaps surrounding the specific impacts of climate change and related systemic risks on food systems and DR-NCDs in SSA.

For peer review only - http://bmjopen.bmj.com/site/about/guidelines.xhtml

The developed conceptual framework tailored to SSA provides valuable insights into the intricate challenges and dynamics of the region's food systems. Given the significant health consequences and difficulties that SSA faces due to climate-induced shifts in food systems, there is a clear and urgent need for action. Understanding and addressing the multifaceted issues embedded within food systems and their intricate interactions with climate change and other systemic factors is imperative.

Contributions

JT, SH, and MK helped with the conceptualisation of the article. JT and MK conducted the screening of studies. JT did data extraction. JT, SH, and MK contributed to the study methodology. SH, MK, SA, and JP contributed to writing – review and editing. JT contributed to writing – original draft.

Conflict of interest

The authors declare no conflict of interest.

For peer review only

For peer review only - http://bmjopen.bmj.com/site/about/guidelines.xhtml

References

1. World Health Organization. Noncommunicable diseases: Key facts. Published online 2022. Accessed June 9, 2023. https://www.who.int/news-room/fact-sheets/detail/noncommunicable-diseases.

2. Gouda HN, Charlson F, Sorsdahl K, Ahmadzada S, Ferrari AJ, Erskine H, et al. Burden of non-communicable diseases in sub-Saharan Africa, 1990–2017: results from the Global Burden of Disease Study 2017. *Lancet Glob Health*. 2019;**7:**e1375-87.

3. World Health Organization. *Sustainable Healthy Diets – Guiding Principles. Rome*. Food & Agriculture Org 2019.

4. Pingault, N, Caron, P, Kalafatic, C. et al. Nutrition and food systems. A report by the High Level Panel of Experts on Food Security and Nutrition of the Committee on World Food Security. 2017.

5. Fanzo J, Davis C (Science writer). Global food systems, diets, and nutrition : linking science, economics, and policy. *Springer International Publishing* 2021.

6. HLPE. Food security and nutrition: building a global narrative towards 2030. *Report by the High Level Panel of Experts on Food Security and Nutrition of the Committee on World Food Security, Rome*.

7. Turner C, Aggarwal A, Walls H, Herforth A, Drewnowski A, Coates J, et al. Concepts and critical perspectives for food environment research: a global framework with implications for action in low-and middle-income countries. *Glob Food Sec*. 2018;**18**:93-101.

8. Frumkin H, Haines A. Global environmental change and noncommunicable disease risks. *Annu Rev Public Health*. 2019;**40**:261-82.

9. Pachauri RK, Allen MR, Barros VR, Broome J, Cramer W, Christ R, et al. Climate change 2014: synthesis report. Contribution of Working Groups I, II and III to the fifth assessment report of the Intergovernmental Panel on Climate Change. Ipcc; 2014.

10. Fanzo J, Davis C, McLaren R, Choufani J. The effect of climate change across food systems: Implications for nutrition outcomes. *Glob Food Sec*. 2018;**18:**12–9.

11. Stefan DC, Elzawawy AM, Khaled HM, Ntaganda F, Asiimwe A, Addai BW, et al. Developing cancer control plans in Africa: examples from five countries. *Lancet Oncol*. 2013;**14**:e189-e195.

12. Trudinger H, Crimp S, Friedman RS. Food systems in the face of climate change: reviewing the state of research in South Pacific Islands. *Reg Environ Change*. 2023;**23**:45.

13. Hawkes C, Parsons K. Tackling Food Systems Challenges: The Role of Food Policy. London, United Kingdom. 2019.

For peer review only - http://bmjopen.bmj.com/site/about/guidelines.xhtml

14. Hunter-Adams J, Battersby J. Health care providers' perspectives of diet-related non-communicable disease in South Africa. *BMC Public Health*. 2020;**20**; 1-10.

15. World Health Organization. Systems thinking for noncommunicable disease prevention policy: guidance to bring systems approaches into practice. World Health Organization. Regional Office for Europe; 2022.

16. Swinburn BA, Kraak VI, Allender S, Atkins VJ, Baker PI, Bogard JR, et al. The global syndemic of obesity, undernutrition, and climate change: *The lancet*. 2019;**393**:791-846.

17. Springmann M, Mason-D'Croz D, Robinson S, Garnett T, Godfray HCJ, Gollin D, et al. Global and regional health effects of future food production under climate change: a modelling study. *Lancet*. 2016;**387**:1937-46.

18. Fanzo J, Miachon L. Harnessing the connectivity of climate change, food systems and diets: Taking action to improve human and planetary health. *Anthropocene*. 2023;**42**:100381.

19. Heinrich, M. IARC Monographs on the Evaluation of Carcinogenic Risks to Humans: Some Traditional Herbal Medicines, Some Mycotoxins, Naphthalene and Styrene-IARC Working Group on the Evaluation of Carcinogenic Risks to Humans, 2002, Lyon (France), International Agency for Research on Cancer (IARC) IARC Monographs on the Evaluation of Carcinogenic Risks to Humans, Vol. 82, 594 pp., cumulative cross index to IARC Monographs on the Evaluation of Carcinogenic Risks to Humans. *Journal of Ethnopharmacology* 2003;***2:***299-300.

20. Devereux S. The impact of droughts and floods on food security and policy options to alleviate negative effects. *Agricultural Economics* 2007;**37**:47–58.

21. Thornton PK, Ericksen PJ, Herrero M, Challinor AJ. Climate variability and vulnerability to climate change: a review. *Glob Chang Biol*. 2014;**20:**3313-28.

22. Scott-Villiers P, Chisholm N, Wanjiku Kelbert A, Hossain N. Precarious lives: work, food and care after the global food crisis. 2016.

23. Davis GC. The American Rescue Plan Act Is a Great Start but More Increases in Supplemental Nutrition Assistance Program (SNAP) Benefits Are Likely Needed Due to Implicit Hidden Reductions. J Nutr 2021;**151:**2099–104.

24. Davis KF, Downs S, Gephart JA. Towards food supply chain resilience to environmental shocks. *Nat Food* 2021;**2:**54–65.

25. Peters MDJ, Marnie C, Tricco AC, Pollock D, Munn Z, Alexander L, et al. Updated methodological guidance for the conduct of scoping reviews. *JBI Evid Synth*. 2020;**18**;2119-26.

26. Page MJ, McKenzie JE, Bossuyt PM, Boutron I, Hoffmann TC, Mulrow CD, et al. The PRISMA 2020 statement: an updated guideline for reporting systematic reviews. *Syst Rev*. 2021;**10**:1-11.

For peer review only - http://bmjopen.bmj.com/site/about/guidelines.xhtml

27. L. A. G. United Nations Framework Convention on Climate Change. *Environ Health Perspect*,. 2000;**108:**A353-A353.

28. Race to Zero. From 1800 to COP26: A history of key climate conversations [Internet]. 2021 [cited 2023 Jun 2]. Available from: https://climatechampions.unfccc.int/from-1800-to-cop26-a-history-of-key-climate-conversations/

29. Ouzzani M, Hammady H, Fedorowicz Z, Elmagarmid A. Rayyan—a web and mobile app for systematic reviews. Syst Rev. 2016;**5**:1–10.

30. Noyes J, Booth A, Moore G, Flemming K, Tunçalp Ö, Shakibazadeh E. Synthesising quantitative and qualitative evidence to inform guidelines on complex interventions: clarifying the purposes, designs and outlining some methods. *BMJ Glob Health* 2019;**4:**e000893.

31. Omer RE, Bakker MI, van P, A P Hoogenboom RL, G Polman TH, Alink GM, et al. Aflatoxin and liver cancer in Sudan. *Nutr Cancer* 1998;**32:**174-180.

32. Lontchi-Yimagou E, Tsalefac M, Tapinmene LMT, Noubiap JJN, Balti EV, Nguewa JL, et al. Seasonality in diabetes in Yaounde, Cameroon: a relation with precipitation and temperature. *BMC Public Health* 2016;**16**:1-7

33. Odonkor ST, Sallar AM. An Assessment of Public Knowledge and Potential Health Impacts of Global Warming in Ghana. *Biomed Res Int* 2020.

34. Ngwira A. Climate and location as determinants of childhood stunting, wasting, and overweight: an application of semiparametric multivariate probit model. *Nutrition* 2020;**70**:100010..

35. Kasomo JM, Gayawan E. Spatial location, temperature and rainfall diversity affect the double burden of malnutrition among women in Kenya. *SSM Popul Health*. 2021;**16**:100939.

36. Akello J, Ortega-Beltran A, Katati B, Atehnkeng J, Augusto J, Mwila CM, et al. Prevalence of Aflatoxin- and Fumonisin-Producing Fungi Associated with Cereal Crops Grown in Zimbabwe and Their Associated Risks in a Climate Change Scenario. *Foods* 2021;**10**:287.

37. Kimanya ME, Michael NR, Emmanuel M, Chibundu NE, Candida PS, Yun YG. Estimating the risk of aflatoxin-induced liver cancer in Tanzania based on biomarker data. *PLoS One* 2021;**16:**e0247281.

38. Greibe Andersen J, Karekezi C, Ali Z, Yonga G, Kallestrup P, Kraef C. Perspectives of local community leaders, health care workers, volunteers, policy makers and academia on climate change related health risks in Mukuru informal settlement in Nairobi, Kenya—a qualitative study. *Int J Environ Res Public Health* 2021;**18:**12241.

39. Kortei NK, Annan T, Boakye AA, Essuman EK, Tettey CO, Kyei-Baffour V. Aflatoxin M1 exposure in a fermented millet-based milk beverage 'brukina'and its cancer risk characterization in Greater Accra, Ghana. *Sci Rep* 2022;**12:**12562.

For peer review only - http://bmjopen.bmj.com/site/about/guidelines.xhtml

40. Sirma AJ, Makita K, Randolph DG, Senerwa D, Lindahl JF. Aflatoxin Exposure from Milk in Rural Kenya and the Contribution to the Risk of Liver Cancer. *Toxins* 2019;**11:**469.

41. Bbosa GS, Kitya D, Lubega A, Ogwal-Okeng J, Anokbonggo WW, Kyegombe DB. Review of the biological and health effects of aflatoxins on body organs and body systems. *Aflatoxins-recent advances and future prospects* 2013;**12**:239–65.

42. Saha Turna N, Wu F. Risk assessment of aflatoxin-related liver cancer in Bangladesh. *Food Additives & Contaminants: Part A* 2019;**36**:320–6.

43. Saha Turna N, Havelaar A, Adesogan A, Wu F. Aflatoxin M1 in milk does not contribute substantially to global liver cancer incidence. *Am J Clin Nutr* 2022;**115**:1473–80.

44. IARC. Monographs on the evaluation of carcinogenic risks to humans: Overall evaluations of carcinogenicity: An updating of IARC Monographs. Lyon, France: *International Agency for Research on Cancer* 1987;1–403.

45. Zilbermint M. Diabetes and climate change. *J Community Hosp Intern Med Perspect* 2020;**10**:409–12.

For peer review only

For peer review only - http://bmjopen.bmj.com/site/about/guidelines.xhtml

For peer review only

For peer review only - http://bmjopen.bmj.com/site/about/guidelines.xhtml

For peer review only

For peer review only - http://bmjopen.bmj.com/site/about/guidelines.xhtml

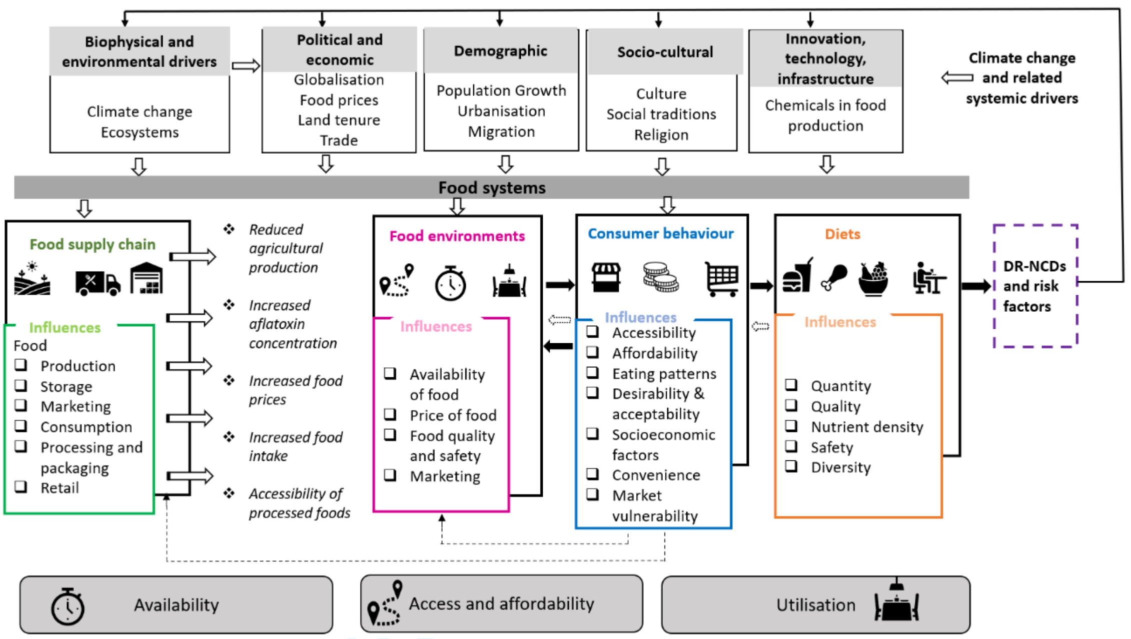

Figure 1: Food Systems Conceptual Framework for Nutrition and DR-NCDs, adapted from the HLPE framework [4]

For peer review only

For peer review only - http://bmjopen.bmj.com/site/about/guidelines.xhtml

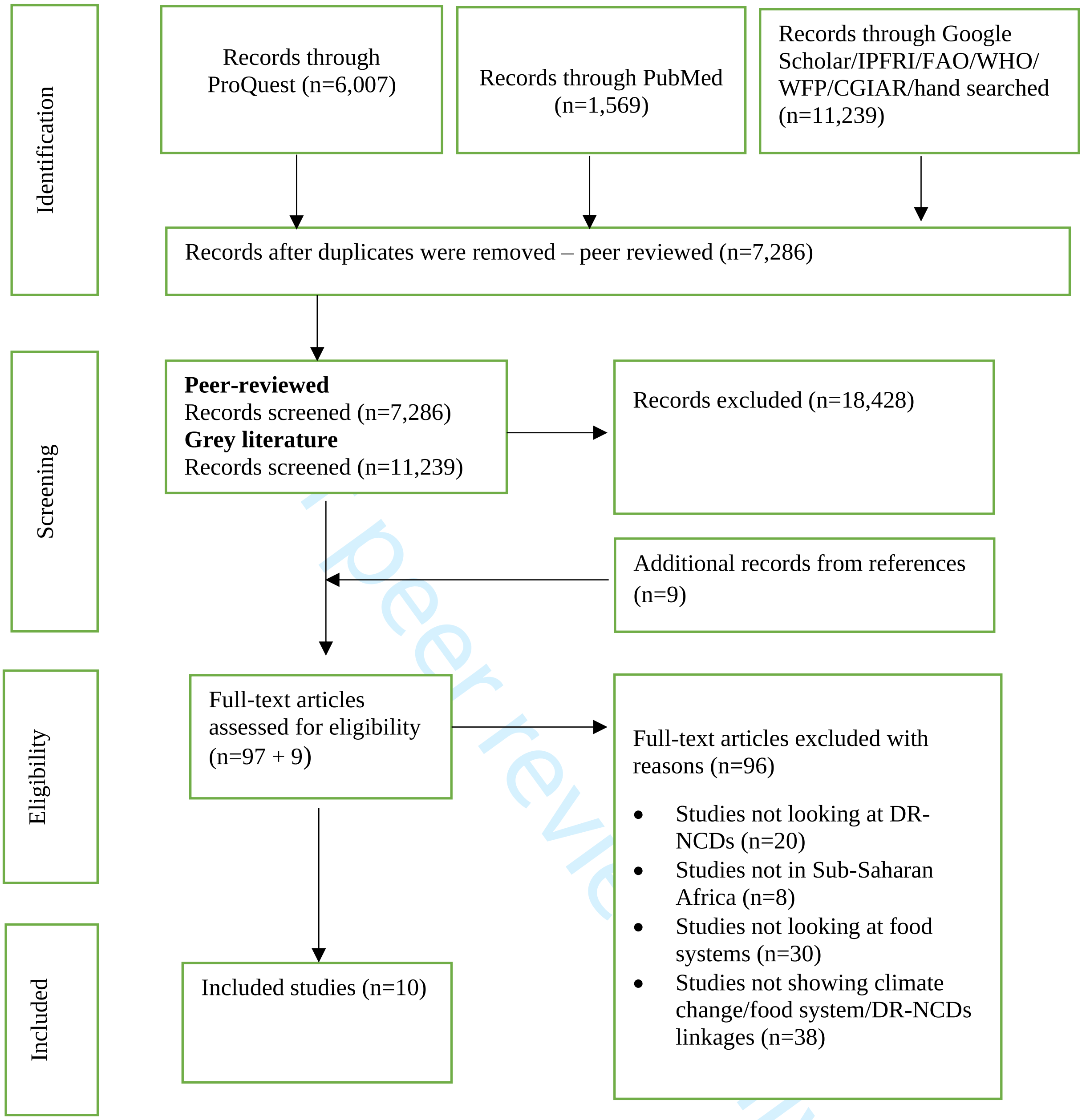

Figure 2: PRISMA framework

For peer review only - http://bmjopen.bmj.com/site/about/guidelines.xhtml

Table 1: Overview of included studies

Author	Year	Country	Study design	Aspect of climate change	DR-NCDs and risk factors
Omer [31]	1998	Sudan	Cross-sectional survey	Humidity	Liver cancer
Lontchi-Yimagou [32]	2016	Cameroon	Retrospective study	Temperature and rainfall	Diabetes
Sirma [40]	2019	Kenya	Cross-sectional survey	Precipitation, humidity, floods, droughts, temperature	Liver cancer
Odonkor & Sallar [33]	2020	Ghana	Descriptive cross-sectional design	Global warming	Heart disease
Ngwira 2020 [34]	2020	Malawi	Cross-sectional analysis	Rainfall	Overweight
Kasomo [35]	2021	Kenya	Cross-sectional analysis	Temperature and rainfall	Overweight, obesity
Akello [36]	2021	Zimbabwe	Cross-sectional analysis	Humidity	Liver cancer
Kimanya [37]	2021	Tanzania	Cross-sectional survey	Humidity and temperature	Liver cancer
Andersen [38]	2021	Kenya	Qualitative	Climate change	Diabetes, hypertension, heart attack, cancer, and obesity
Kortei [39]	2022	Ghana	Cross-sectional survey	Humidity	Liver cancer

For peer review only

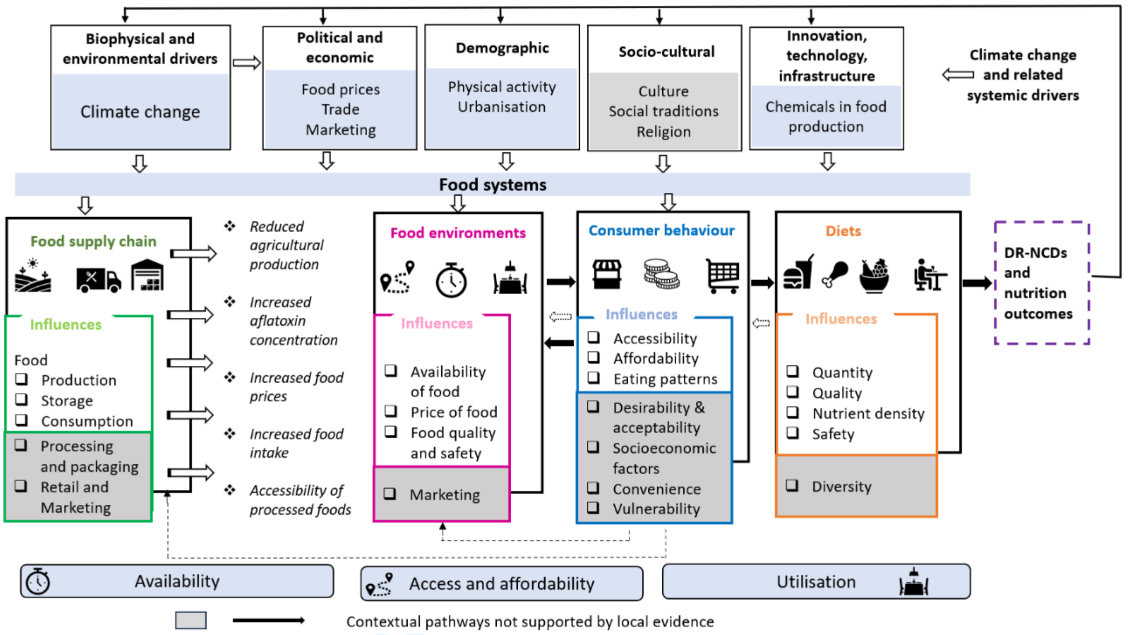

Figure 3: Conceptual framework of food systems for DR-NCDs and nutrition in Sub-Saharan Africa

For peer review only

Table 2: Description of studies included in the review by food system pathway.

Author/ year	Countr y	Study design	Method of analysis	Study objectives	Sample size	Climate change component	Outcome	Key findings
**Food production pathway**								
Andersen 2021 [38]	Kenya	Qualitative	Descriptive Statistics	To explore knowledge and perspectives on climate change and health-related issues, with a particular focus on noncommunicable diseases	28	Climate change	Diabetes, hypertensio n, heart attack, lung disease, cancer, and obesity	Climate change-induced food insecurity and rising food prices emerged as prominent challenges.
Kasomo 2021 [35]	Kenya	Cross-sectional analysis	Inferential Statistics	To quantify spatial variations and estimate the effect of climatic and environmental factors on under- and over-nutrition among women in Kenya	40,300	Temperatur e and precipitatio n	Obesity and overweight	Higher precipitation levels were associated with an increased likelihood of women being overweight and obese.
Ngwira 2020 [34]	Malawi	Cross-sectional analysis	Multivariate probit model	To investigate the effects of climate and location using the multivariate model of malnutrition	5,149	Rainfall	Overweight	A positive correlation was observed between rainfall and overweight individuals.

For peer review only

Odonkor & Sallar 2020 [33]	Ghana	Descriptive cross-sectional design	Inferential Statistics	To investigate public knowledge of global warming and its effects on human health	1,130	Global warming	Heart disease	Rising food prices impact the affordability and availability of food in urban areas.
**Food storage pathway**								
Akello 2021 [36]	Zimbabwe	Cross-sectional analysis	Inferential Statistics	To assess frequencies of mycotoxin-producing fungi in maize, sorghum, pearl millet, and finger millet cropped and traded in Zimbabwe	NI[1]	Humidity	Liver cancer	Consumption of aflatoxin-contaminated maize stored for 4-6 months increased liver cancer rates.
Kimanya 2021 [37]	Tanzania	Cross-sectional analysis	Inferential Statistics	To estimate the population risk of aflatoxin-induced liver cancer	NI[1]	Temperature and humidity	Liver cancer	Population risk for aflatoxin-induced liver cancer nationwide was estimated to be 2.95 cases per 100,000 people.
Kortei 2022 [39]	Ghana	Cross-sectional survey	NI	To evaluate AFM_1 levels and cancer risks associated	150	Temperature and humidity	Liver cancer	Consumption of *burkina* showed varied risk

[1] NI-Not indicated

For peer review only - http://bmjopen.bmj.com/site/about/guidelines.xhtml

				with *burkina* (a millet-based fermented milk product)				levels of liver cancer.
Omer 1998 [31]	Sudan	Cross-sectional survey	Inferential Statistics	To investigate whether aflatoxin contamination of peanut products may contribute to the incidence of hepatocellular carcinoma (HCC) in Sudan	54	Temperature and humidity	Liver cancer	Aflatoxin contamination of peanuts has a significant association with liver cancer.
Sirma 2019 [40]	Kenya	Cross-sectional survey	Inferential Statistics	To assess the risk of liver cancer posed by AFM_1 in milk, assuming 10-fold lower carcinogenicity than AFB_1	NI[1]	Rainfall, droughts, humidity, temperature, floods	Liver cancer	Aflatoxin exposure from milk contributed a small percentage to liver cancer incidence rates.
**Food consumption pathway**								
Lontchi-Yimagou 2016 [32]	Cameroon	Retrospective study	Inferential Statistics	To investigate the relationship between diabetes hospitalisation rates and climate variations in Yaoundé	818	Temperature and precipitation	Diabetes	Diabetes admissions were higher during rainy seasons due to nutritional variability.

For peer review only - http://bmjopen.bmj.com/site/about/guidelines.xhtml

For peer review only

For peer review only - http://bmjopen.bmj.com/site/about/guidelines.xhtml

PRISMA 2020 Checklist

Section and Topic	Item #	Checklist item	Location where item is reported
**TITLE**			
Title	1	Identify the report as a systematic review.	1
**ABSTRACT**			
Abstract	2	See the PRISMA 2020 for Abstracts checklist.	2
**INTRODUCTION**			
Rationale	3	Describe the rationale for the review in the context of existing knowledge.	4-5
Objectives	4	Provide an explicit statement of the objective(s) or question(s) the review addresses.	5
**METHODS**			
Eligibility criteria	5	Specify the inclusion and exclusion criteria for the review and how studies were grouped for the syntheses.	8
Information sources	6	Specify all databases, registers, websites, organisations, reference lists and other sources searched or consulted to identify studies. Specify the date when each source was last searched or consulted.	8
Search strategy	7	Present the full search strategies for all databases, registers and websites, including any filters and limits used.	8 and 24
Selection process	8	Specify the methods used to decide whether a study met the inclusion criteria of the review, including how many reviewers screened each record and each report retrieved, whether they worked independently, and if applicable, details of automation tools used in the process.	9
Data collection process	9	Specify the methods used to collect data from reports, including how many reviewers collected data from each report, whether they worked independently, any processes for obtaining or confirming data from study investigators, and if applicable, details of automation tools used in the process.	6-9
Data items	10a	List and define all outcomes for which data were sought. Specify whether all results that were compatible with each outcome domain in each study were sought (e.g. for all measures, time points, analyses), and if not, the methods used to decide which results to collect.	
	10b	List and define all other variables for which data were sought (e.g. participant and intervention characteristics, funding sources). Describe any assumptions made about any missing or unclear information.	9
Study risk of bias assessment	11	Specify the methods used to assess risk of bias in the included studies, including details of the tool(s) used, how many reviewers assessed each study and whether they worked independently, and if applicable, details of automation tools used in the process.	9
Effect measures	12	Specify for each outcome the effect measure(s) (e.g. risk ratio, mean difference) used in the synthesis or presentation of results.	
Synthesis methods	13a	Describe the processes used to decide which studies were eligible for each synthesis (e.g. tabulating the study intervention characteristics and comparing against the planned groups for each synthesis (item #5)).	9
	13b	Describe any methods required to prepare the data for presentation or synthesis, such as handling of missing summary statistics, or data conversions.	
	13c	Describe any methods used to tabulate or visually display results of individual studies and syntheses.	
	13d	Describe any methods used to synthesize results and provide a rationale for the choice(s). If meta-analysis was performed, describe the model(s), method(s) to identify the presence and extent of statistical heterogeneity, and software package(s) used.	
	13e	Describe any methods used to explore possible causes of heterogeneity among study results (e.g. subgroup analysis, meta-regression).	
	13f	Describe any sensitivity analyses conducted to assess robustness of the synthesized results.	
Reporting bias assessment	14	Describe any methods used to assess risk of bias due to missing results in a synthesis (arising from reporting biases).	10
Certainty	15	Describe any methods used to assess certainty (or confidence) in the body of evidence for an outcome.	

For peer review only - http://bmjopen.bmj.com/site/about/guidelines.xhtml

PRISMA 2020 Checklist

Section and Topic	Item #	Checklist item	Location where item is reported
assessment			
**RESULTS**			
Study selection	16a	Describe the results of the search and selection process, from the number of records identified in the search to the number of studies included in the review, ideally using a flow diagram.	10
	16b	Cite studies that might appear to meet the inclusion criteria, but which were excluded, and explain why they were excluded.	
Study characteristics	17	Cite each included study and present its characteristics.	11
Risk of bias in studies	18	Present assessments of risk of bias for each included study.	
Results of individual studies	19	For all outcomes, present, for each study: (a) summary statistics for each group (where appropriate) and (b) an effect estimate and its precision (e.g. confidence/credible interval), ideally using structured tables or plots.	11-16
Results of syntheses	20a	For each synthesis, briefly summarise the characteristics and risk of bias among contributing studies.	
	20b	Present results of all statistical syntheses conducted. If meta-analysis was done, present for each the summary estimate and its precision (e.g. confidence/credible interval) and measures of statistical heterogeneity. If comparing groups, describe the direction of the effect.	
	20c	Present results of all investigations of possible causes of heterogeneity among study results.	
	20d	Present results of all sensitivity analyses conducted to assess the robustness of the synthesized results.	
Reporting biases	21	Present assessments of risk of bias due to missing results (arising from reporting biases) for each synthesis assessed.	
Certainty of evidence	22	Present assessments of certainty (or confidence) in the body of evidence for each outcome assessed.	
**DISCUSSION**			
Discussion	23a	Provide a general interpretation of the results in the context of other evidence.	17
	23b	Discuss any limitations of the evidence included in the review.	18
	23c	Discuss any limitations of the review processes used.	18
	23d	Discuss implications of the results for practice, policy, and future research.	3
**OTHER INFORMATION**			
Registration and protocol	24a	Provide registration information for the review, including register name and registration number, or state that the review was not registered.	7
	24b	Indicate where the review protocol can be accessed, or state that a protocol was not prepared.	7
	24c	Describe and explain any amendments to information provided at registration or in the protocol.	
Support	25	Describe sources of financial or non-financial support for the review, and the role of the funders or sponsors in the review.	9
Competing interests	26	Declare any competing interests of review authors.	19
Availability of data, code and other materials	27	Report which of the following are publicly available and where they can be found: template data collection forms; data extracted from included studies; data used for all analyses; analytic code; any other materials used in the review.	

For peer review only - http://bmjopen.bmj.com/site/about/guidelines.xhtml

From: Page MJ, McKenzie JE, Bossuyt PM, Boutron I, Hoffmann TC, Mulrow CD, et al. The PRISMA 2020 statement: an updated guideline for reporting systematic reviews. BMJ 2021;372:n71. doi:

PRISMA 2020 Checklist

10.1136/bmj.n71

For more information, visit: http://www.prisma-statement.org/

For peer review only

Climate Change-induced Shifts in Food Systems and Diet-related Noncommunicable Diseases in Sub-Saharan Africa: A Scoping Review and a Conceptual Framework

Journal:	*BMJ Open*
Manuscript ID	bmjopen-2023-080241.R1
Article Type:	Original research
Date Submitted by the Author:	17-Mar-2024

Complete List of Authors:	Tapkigen, Janet; Tampere University, Department of Health Sciences, Faculty of Social Sciences<br>Harding, Seeromanie; King's College London, Department of Population Health Sciences, School of Life Course & Population Sciences<br>Jutta, Pulkki; Tampere University, Department of Health Sciences, Faculty of Social Sciences<br>Atkins, Salla; Tampere University, Department of Health Sciences, Faculty of Social Sciences; Karolinska Institute, Department of Global Public Health<br>Koivusalo, Meri; Tampere University, Department of Health Sciences, Faculty of Social Sciences

**Primary Subject Heading**:	Public health
Secondary Subject Heading:	Global health
Keywords:	EPIDEMIOLOGY, PUBLIC HEALTH, Climate Change

SCHOLARONE™ Manuscripts

I, the Submitting Author has the right to grant and does grant on behalf of all authors of the Work (as defined in the below author licence), an exclusive licence and/or a non-exclusive licence for contributions from authors who are: i) UK Crown employees; ii) where BMJ has agreed a CC-BY licence shall apply, and/or iii) in accordance with the terms applicable for US Federal Government officers or employees acting as part of their official duties; on a worldwide, perpetual, irrevocable, royalty-free basis to BMJ Publishing Group Ltd ("BMJ") its licensees and where the relevant Journal is co-owned by BMJ to the co-owners of the Journal, to publish the Work in this journal and any other BMJ products and to exploit all rights, as set out in our licence.

The Submitting Author accepts and understands that any supply made under these terms is made by BMJ to the Submitting Author unless you are acting as an employee on behalf of your employer or a postgraduate student of an affiliated institution which is paying any applicable article publishing charge ("APC") for Open Access articles. Where the Submitting Author wishes to make the Work available on an Open Access basis (and intends to pay the relevant APC), the terms of reuse of such Open Access shall be governed by a Creative Commons licence – details of these licences and which Creative Commons licence will apply to this Work are set out in our licence referred to above.

Other than as permitted in any relevant BMJ Author's Self Archiving Policies, I confirm this Work has not been accepted for publication elsewhere, is not being considered for publication elsewhere and does not duplicate material already published. I confirm all authors consent to publication of this Work and authorise the granting of this licence.

For peer review only

Title: Climate Change-induced Shifts in Food Systems and Diet-related Noncommunicable Diseases in Sub-Saharan Africa: A Scoping Review and a Conceptual Framework

Authors

Tapkigen, Janet [1]; Harding, Seeromanie [2]; Pulkki, Jutta [1]; Atkins, Salla [1,3]; Koivusalo, Meri [1,4]

Affiliation

[1]Department of Health Sciences, Faculty of Social Sciences, Tampere University, Tampere, Finland.

[2]Department of Population Health Sciences, School of Life Course & Population Sciences, King's College London, London, United Kingdom.

[3]Department of Global Public Health, Karolinska Institutet, Stockholm, Sweden.

[4]Centre for Health in All Policies and Social Determinants of Health, Tampere University, Finland.

Corresponding Author

Janet Tapkigen, MSc.,
Tampere University,
Faculty of Social Sciences,
Arvo Ylpön Katu 34, 33520, Tampere, Finland.
Email: janet.tapkigen@tuni.fi
Tel : +358 (0) 451378262

Keywords: Climate change, Noncommunicable Disease, One health

Word count: 6208

For peer review only

For peer review only - http://bmjopen.bmj.com/site/about/guidelines.xhtml

Abstract

Objectives: To determine the relationship between climate change, food systems, and diet-related noncommunicable diseases (DR-NCDs) in Sub-Saharan Africa (SSA) and propose a conceptual framework for food systems in SSA.

Design: A scoping review

Eligibility criteria: Studies included investigated the relationship between climate change and related systemic risks, food systems, DR-NCDs and its risk factors in SSA. Studies focusing on the association between climate change and DR-NCDs unrelated to food systems, such as social inequalities, were excluded.

Sources of evidence: A comprehensive search was conducted in ProQuest (9 databases), Google Scholar and PubMed in December 2022.

Charting methods: Data extracted from studies included: author, study type, country of study, climate change component, DR-NCD outcomes and risk factors, and impacts of climate change on DR-NCDs. A narrative approach was used to analyse the data. Based on the evidence gathered from SSA, we modified an existing food system conceptual framework.

Results: The search retrieved 19,125 studies, ten of which were included in the review. Most studies used a cross-sectional design (n=8). Four explored the influence of temperature on liver cancer through food storage while four explored the influence of temperature and rainfall on diabetes and obesity through food production. Cross-sectional evidence suggested that temperature is associated with liver cancer and rainfall with diabetes.

Conclusion: The review highlights the vulnerability of SSA's food systems to climate change-induced fluctuations, which in turn affect dietary patterns and DR-NCD outcomes. The evidence is scarce and concentrates mostly on the health effects of temperature through food storage. It proposes a conceptual framework to guide future research addressing climate change and DR-NCDs in SSA.

For peer review only - http://bmjopen.bmj.com/site/about/guidelines.xhtml

Strengths and limitations of this study

- This scoping review followed the Joanna Briggs Institute methodology for scoping reviews.
- The methodology and search terms involved an extensive search of literature.
- Existing food system framework conceptually guided the methodology and development of hypotheses.
- Despite efforts to search for grey literature, country-specific documents may have been missed; this study is based on published data and, therefore, is subject to publication bias.
- The studies that met the inclusion criterion were primarily observational and descriptive; thus, while these provided valuable insights for hypotheses, their contribution to evidence regarding causality is limited.

For peer review only

Introduction

Noncommunicable diseases (NCDs) account for 74% (41 million) of all deaths globally every year (1). The primary causes of NCD-related deaths are cardiovascular diseases (accounting for 17.9 million deaths annually), cancer (9.3 million), chronic respiratory diseases (4.1 million), and diabetes (2.0 million) (1) . Collectively, these diseases contribute to 80% of all NCD-related deaths (1). In Sub-Saharan Africa (SSA), cardiovascular diseases are the leading cause of NCD-related deaths, accounting for 15.1% of the NCD burden. Diabetes and liver cancer also make significant contributions to the NCD burden in the region, accounting for 4.2% and 0.8% of NCD-related deaths, respectively (2). These three diseases – cardiovascular diseases, liver cancer, and diabetes – are known as diet-related NCDs (DR-NCDs), as they are primarily caused by poor dietary habits, characterised by highly processed foods high in saturated fats, sugars, and salt (3).

To effectively address the burden of DR-NCDs in SSA, it is essential to understand how the food systems function, what their drivers are, and their role in shaping diets. Food systems can be described as interconnected networks comprising the environment, people, processes, infrastructure, and institutions involved in various aspects of food production, processing, transportation, storage, distribution, retailing, and consumption (4). The outcomes of these activities and their governance collectively define the functioning of the food system (4). The interconnected components of food systems, namely the food supply chain, food environments, and consumer behaviour, are illustrated in Figure 1.

The food supply chains encompass all stages from production to waste disposal (5), and they depend on ecological, human, energy, and economic systems to produce and distribute foods (6). On the other hand, food environments encompass the settings where consumers make food-related choices (5), therefore connecting food supply and demand (6). Food environments are divided into personal environments (accessibility, affordability, convenience, desirability) and external environments (food vendors, prices, income levels, knowledge, sociocultural norms, marketing practices, regulations) (7). For certain individuals, these environments are local (involving self-produced or locally sourced foods), while for others, they are global (encompassing foods from regional and international sources) (5).

Food environments influence consumer behaviour (6), which refers to the decisions and actions individuals take when acquiring and consuming food (4). These decisions shape diets in terms of quantity, quality, diversity, safety, and adequacy of food (6). Consequently, these diets exert influence on interconnected systems, including nutrition outcomes that directly impact healthcare systems and the climate impacts of diets that affect ecosystems (6).

SSA is currently facing a food systems challenge related to climate change, poor agricultural practices, food insecurity, and the consequences on DR-NCD risk. Climate change threatens the region's ecosystems, agricultural productivity, and food security, exacerbating existing vulnerabilities. Climate change, driven by human activities, has been widely acknowledged as a significant contributor to the development and exacerbation of DR-NCDs (8). The rising global temperatures, erratic rainfall patterns, and changing patterns of extreme weather events

For peer review only - http://bmjopen.bmj.com/site/about/guidelines.xhtml

significantly influence agricultural and food systems (9). These climatic shifts impact food quality, availability, and affordability (9, 10), potentially altering food access and choices, with implications for nutrition and dietary habits (10). As food systems are undergoing profound transformations driven by population growth, globalisation, technology, urbanisation, trade liberalisation, migration, policies, competition for essential resources (water, energy, land), and shifts in income distribution, the impacts of climate change compound the effects of these drivers (4). As such, climate change can be seen as amplifying the underlying drivers associated with DR-NCDs (8, 9).

To effectively address the burden of DR-NCDs in SSA amid these climate-induced changes, a food systems approach is necessary (11). Unlike disease-oriented approaches, food systems approach considers the entire food system — from production to consumption (12). Existing interventions for NCDs in SSA have not focused on multisectoral pathways on determinants of health (13, 14). Health promotion activities to prevent NCDs have focused on individual responsibility and influencing purchase patterns more than a systems approach where the type and availability of food itself is considered. Therefore, shifting towards a food systems approach allows for a holistic and comprehensive understanding of the entire food system, recognising the intricate interplay and feedback loops between different risk factors for NCDs (15).

In the context of addressing DR-NCDs, adopting a systems thinking approach acknowledges that one system alone, for example, the healthcare system, cannot solely determine health outcomes (16). Instead, it recognises the significant role interconnected systems (such as the environment, energy, and government sectors) play in shaping environments that impact DR-NCDs (16). However, implementing a food systems approach in SSA comes with challenges resulting from underreporting underlying determinants of DR-NCDs (17) and limited evidence on the specific pathways in which climate change and related systemic risks influence food systems and, in turn, on DR-NCDs in the region.

Existing food system frameworks have explored the broad-scale interactions and pathways in which climate change influences the risks associated with nutrition outcomes. For instance, the High-level Panel of Experts (HLPE) (18) developed a food systems framework in 2017, illustrating the interplay between diverse drivers within food systems, including climate change, and their effects on health and nutrition outcomes. The HLPE, formed in 2009, is a United Nations body responsible for assessing food security and nutrition research (19). HLPE is governed by 15 world-renowned scientists who are experts in food security and nutrition, and the aim is to assess the current state of food security and nutrition and its underlying causes (19).

While the HLPE framework (18) and previous evidence (8, 20) have provided valuable broad-scale perspectives, there is an urgent need to compile, synthesise and contextualise more localised and region-specific research and to develop contextualised frameworks that can guide future studies. We, therefore, used the original HLPE framework (Figure 1) and existing

For peer review only - http://bmjopen.bmj.com/site/about/guidelines.xhtml

literature to hypothesise and synthesise the causal pathways in which climate change can impact DR-NCDs in SSA.

Aims of the review

This review aims to explore and synthesise research originating from SSA on climate change impacts on food systems and DR-NCDs and to examine if we need to complement and amend the HLPE framework to the SSA context.

For peer review only

For peer review only - http://bmjopen.bmj.com/site/about/guidelines.xhtml

Methods

Conceptual framework development

This study used the original HLPE food systems conceptual framework (18) to hypothesise pathways through which climate change affects food systems and, in turn, DR-NCDs in SSA. The framework is presented in Figure 1 and includes four food supply chain domains: production systems, storage and distribution, processing and packaging, and retail and markets. These supply chains are henceforth referred to as pathways in this review.

In this review, climate change-related systemic risks include any impact of climate change events on other sectors or systems that subsequently influence food systems.

Based on the HLPE framework (18) and knowledge drawn from scientific literature (21-26), we hypothesised pathways in which climate change may affect DR-NCDs and its risk factors (i.e., obesity, overweight) in SSA. These pathways include:

1) The food production pathway. Regarding the *food production pathway,* we hypothesise that the effects of climate change could reduce crop production and yields, consequently reducing food availability. Reduction in food availability could increase food prices, influencing food affordability and, in turn, impact health outcomes (22).

2) The food storage pathway. Under the *food storage pathway,* we propose that reliance on stored crops vulnerable to the impacts of extreme rainfall events can have adverse effects on both the quantity and quality of food. Wet conditions can create a conducive environment for the growth of infectious pathogens. These pathogens, affecting crops during harvesting and storage, could lead to the production of carcinogenic aflatoxins produced by certain moulds, which have been associated with hepatocellular carcinoma, a primary contributor to liver cancer (26).

3) The food distribution and market pathway, and **4) the food processing and packaging pathway**. Within the contexts of *food distribution and market pathways* and *food processing and packaging pathways*, we hypothesise that extreme weather events, such as floods, might disrupt distribution networks, infrastructure, and trade channels, resulting in food losses – especially perishable goods (21, 25). These disruptions can impact the affordability and diversity of food items, impacting food and nutrition security. When comparing fresh perishable foods to processed alternatives, processed foods tend to withstand environmental impacts more effectively, making them a preferable choice within climate variability, albeit with potential trade-offs concerning nutrition value and health implications (21, 25). Moreover, decreased food availability can increase food prices, potentially creating a scenario where imported goods become more easily accessible. Often, these imported products lean towards cost-effectiveness and a high degree of processing.

5) The food retail pathway. In the context of the *food retail pathway*, we hypothesise that extreme weather patterns could reduce crop production and disrupt supply chains for food retailers. Reduced food production and disruptions in the supply chains could lead to shortages of certain food items, affecting consumer choices and potentially changing dietary patterns.

For peer review only - http://bmjopen.bmj.com/site/about/guidelines.xhtml

For instance, if fresh produce becomes scarce due to climate-related disruptions in production and distribution, consumers may opt for less healthy processed alternatives.

6) The food consumption pathway. In the *food consumption pathway*, we posit that climate variability may influence consumption patterns by affecting availability, affordability, and acceptability (27). These shifts could influence food quantity, quality, and prices and reduce income (27). These combined effects can have multifaceted implications for nutritional access, availability, and affordability. This can lead to shifts in dietary preferences, often towards more economical yet highly processed food options (23). The health impact of this pathway is likely to be more important in the light of the focus on processed foods in practice.

Methods

Study design

To augment the HLPE framework with the evidence from the region and to build a context-relevant framework (HLPE-SSA), we conducted a scoping review following the methodological guidance by Joanna Briggs Institute (JBI) (28). The method includes: 1) identifying the research question; 2) inclusion criteria; 3) concept; 4) context; 5) types of evidence sources; 6) search strategy; 7) evidence screening and selection; 8) data extraction; 9) data analysis; and 10) presentation of results.

Scoping reviews are helpful when the literature is complex and serve as a tool to provide policymakers with a comprehensive understanding of the nature of the issue and how that concept has been studied within the existing body of literature (28). This review was reported per the Preferred Reporting Items for Systematic Reviews and Meta-analysis (PRISMA) reporting guidelines (29). This study was based on a registered protocol (https://osf.io/9unbd).

Research question

What kind of research-based evidence exists on climate change, food systems and DR-NCDs relationship from SSA?

Inclusion criteria

We included studies that:

(1) Investigated the relationship between climate change and related systemic risks, food systems, and DR-NCDs and their risk factors;

(2) Were available as full texts;

(3) Were written in any language;

(4) Were related to/conducted in SSA;

Studies were excluded if they:

(1) Examined the association between climate change and DR-NCDs through pathways that do not include food systems, such as social inequalities;

(2) Were not available as full texts;

For peer review only - http://bmjopen.bmj.com/site/about/guidelines.xhtml

(3) Were not conducted in or related to SSA;

(4) Were editorials, letters to the editor, or theoretical discussions of potential impacts or planned programmes that have not yet been implemented, theses, newspapers, magazines, conference abstracts, podcasts, radio programmes, and blogs.

Concept

This review primarily focused on investigating the relationship between climate change and interconnected systemic factors (e.g., urbanisation, globalisation, migration, and food prices), on food systems and subsequently on DR-NCDs and its risk factors.

Context

The scope of this review was centred on examining studies carried out specifically in SSA. These countries are listed in Supplementary Table I. Supplementary Table 1 includes the names of SSA countries, population, GDP per capita, and income category, all retrieved from the World Bank website (30).

Search strategy

The pilot search of PubMed enabled the identification of key terms for the search strategy. The search terms were developed guided by JBI's 'Population, Concept and Context' (PCC) framework and tested in an iterative process to ensure relevance. It included three components: (i) climate change*, (ii) SSA countries, (iii) DR-NCDs*, and nutrition outcomes*. The detailed search strategy is available in Appendix 1. The identified search terms were adapted for other databases. No language restriction was applied in the search. The search started from 1995, when the Global Framework for Climate Change was instituted to address the challenges posed by global warming (31, 32). We included studies published in peer-reviewed journals between 1995 and 2022.

Grey literature search strategy: The search strategy for grey literature was adapted for each data source. The search terms are presented in Appendix 2. Grey literature published from 1995 were considered.

Types of evidence sources

Searches were conducted in ProQuest-9 databases (ProQuest Central, Health and Medical Collection, Environment Science Database, Public Health Collection, Research Library: Health and Medicine, Middle East and Africa Database, Social Science Database, Social Science Premium Collection, and Agriculture Science Database) and PubMed in December 2022. Reference list of relevant citations was further manually searched to identify any articles of interest.

Grey literature data sources: The peer-reviewed searches were supplemented by Google Scholar search in July 2023 involving the first 20 pages. Subsequently, in February 2024, another search was conducted in Google Scholar expanding the search to the first 80 pages as suggested by Haddaway et al. (33) to gain further reach for grey literature. Published and unpublished reports, policy documents and relevant materials from government departments,

For peer review only - http://bmjopen.bmj.com/site/about/guidelines.xhtml

agencies and other organisations were also searched for in google searches, and targeted websites including International Food Policy Research Institute (IFPRI), Consultative Group on International Agricultural Research (CGIAR), World Health Organization (WHO), Food and Agriculture Organization (FAO), World Food Programme (WFP) and World Bank in July 2023. Additionally, a sample of seven government websites in SSA were searched for in February 2024. These seven countries (Kenya, Malawi, Nigeria, Zambia, Mauritius, South Africa, and Botswana) were chosen based on recently reported climate change related disasters (34) and to gain coverage from each income group: lower middle-income, low-income, upper middle-income and high income (35). We searched the publications tab of the Ministry of Health website, Ministry of Agriculture and Environment website, Medical Research Council website, and the national research website.

Evidence screening and selection

Following the search, all identified citations were collated and uploaded into Mendeley, where duplicates were removed. The studies were then uploaded to Rayyan.ai (36) for the title and abstract screening. Two authors, JT and MK independently screened the titles and abstracts. Full texts of relevant studies were assessed for inclusion independently by two reviewers (JT and MK). Disagreements were resolved through discussion.

The search process identified 19,125 studies after removing 1,221 duplicates. Among these, 7,286 studies were peer-reviewed articles, and 11,239 were grey literature. We excluded 18,428 records based on irrelevant titles and abstracts, leaving us with 106 studies for which we retrieved the full texts for detailed assessment. Of these, ten studies met the eligibility criteria and were included in this review. No studies from the grey literature met our inclusion criteria. The search process is depicted in Figure 2.

Data extraction

JT extracted quantitative and qualitative data in Excel using an extraction form developed for the present study (Appendix 3). The data included the following characteristics: author, study type, country of study, climate change component discussed, DR-NCD outcomes and risk factors, and impacts of climate change on DR-NCDs.

Data analysis

A narrative synthesis (37) approach was applied in this review and presented in tables where appropriate. This involved integrating quantitative and qualitative studies through collation, analysis, synthesis, and presentation (37).

Patient or public involvement

None.

For peer review only - http://bmjopen.bmj.com/site/about/guidelines.xhtml

Results

The results are presented under three main sections: 1) characteristics of included studies; 2) results on the influence of climate change on DR-NCDs; and 3) a conceptual framework for food systems and DR-NCDs in SSA. The results are presented according to the identified food system pathways.

Characteristics of included studies

Ten studies met our inclusion criteria (38-47), including eight quantitative (38, 40-44, 46, 47) and two qualitative studies (39) (45). The majority of studies utilised cross-sectional design (n=8) (38, 40-44, 46, 47). The sample size ranged from 28 to 40,300.

Seven studies were published in the 2020s (38-42, 44, 45), two in the 2010s (43, 47) and one in the 1990s (46). Three studies were conducted in Kenya (39, 40, 47), two in Ghana (42, 45), with one study each in Cameroon (43), Malawi (44), Sudan (46), Tanzania (41), and Zimbabwe (38), as shown in Table 1.

Among the studies included, four studies (38, 42, 43, 47) used primary data collection methods to estimate DR-NCD outcomes and its risk factors. Three studies (40, 41, 44) drew upon data from national health sources to recruit participants. All three studies (40, 41, 44), utilising secondary data, relied on original survey data to measure and estimate health outcomes.

Three studies (38, 42, 47) used AF and FM levels from grain and milk samples to quantify dietary exposure and estimate the risk of liver cancer. Meanwhile, five studies (40, 41, 43, 44, 46) used human samples to assess dietary exposure and DR-NCD risk. Notably, two studies (39, 45) took an alternate approach, looking at participants' perception to illustrate health risks associated with climate change.

Table 1: Overview of included studies

Author	Year	Country	Study design	Aspect of climate change	DR-NCDs and risk factors
Omer (46)	1998	Sudan	Cross-sectional survey	Temperature (related to increased humidity)	Liver cancer
Lontchi-Yimagou (43)	2016	Cameroon	Retrospective study	Temperature and rainfall	Diabetes
Sirma (47)	2019	Kenya	Cross-sectional survey	Rainfall, droughts, temperature	Liver cancer
Odonkor & Sallar (45)	2020	Ghana	Descriptive qualitative	Global warming	Heart disease
Ngwira (44)	2020	Malawi	Cross-sectional analysis	Rainfall	Overweight
Kasomo (40)	2021	Kenya	Cross-sectional analysis	Temperature and rainfall	Overweight, obesity
Akello (38)	2021	Zimbabwe	Cross-sectional analysis	Drought, long dry spells (related to humidity)	Liver cancer
Kimanya (41)	2021	Tanzania	Cross-sectional survey	Temperature (related to increased humidity)	Liver cancer
Andersen (39)	2021	Kenya	Qualitative	Climate change	Diabetes, hypertension, heart attack, and obesity
Kortei (42)	2022	Ghana	Cross-sectional survey	Temperature (related to increased humidity)	Liver cancer

The most common climate variable investigated was temperature (related to increased humidity), reported in 5 out of 10 studies (38, 41, 42, 46, 47). In this review, if a study examined the association between temperature and subsequent rise in humidity levels (above 14%

For peer review only - http://bmjopen.bmj.com/site/about/guidelines.xhtml

For peer review only

moisture content), we classified the climate variable as temperature (related to increased humidity). Four studies investigated DR-NCD outcomes related to variation in rainfall (40, 43, 44, 47), and temperature (40, 41, 43, 47).

Five cross-sectional studies measured (41, 46) and estimated (38, 42, 47) liver cancer rates. Two studies looked at the relationship between climate events and diabetes (39, 43). Three studies examined overweight (40, 44) or obesity (40), two heart disease (39, 43), and one hypertension (39).

Food system pathways through which climate change influences nutrition outcomes and DR-NCDs in SSA

Table 2 shows the results on how climate change influences DR-NCDs. The table is organised based on the food system pathways identified from the theoretical literature and presented below. From the six pathways hypothesised, we found evidence to four.

Food production and retail pathway

Related to food production and retail pathways, four studies examined the relationships between precipitation, temperature, and various health outcomes, including obesity (40), overweight (40, 44), heart disease (45), and DR-NCDs in general (39).

Diabetes, hypertension, heart attack, cancer, overweight, and obesity risk: Two studies (39, 40), indicated a reduction in food production due to variable rainfall and temperature. Findings showed that as annual precipitation and temperature increase due to climate change, so does the likelihood of women being overweight and obese. This was attributed to global warming, which potentially led to water scarcity in Kenya, reduced availability of nutritious crops, and increased reliance on non-nutritious foods (40). In addition, due to the rising temperatures, rainfall patterns have been unpredictable, causing droughts and famines and thus prompting food insecurity and changes in dietary patterns (40). Another study attributed the occurrence of diabetes, hypertension, heart attack, cancer, and obesity to harsh weather patterns, which disrupted agricultural production, resulting in higher costs of food in urban areas due to reliance on imported produce (39). Additionally, climate change-driven reliance on chemically treated food production raised concerns about food quality (39).

Overweight risk: Contrary to other studies, one study suggested that rainfall variability could increase food production, potentially contributing to overweight. The author argued that high rainfall is often associated with better yields fostering overeating behaviours, which may enhance the risk of overweight (44).

Heart disease risk: The association of global warming with food production and heart disease was assessed in Ghana. Respondents perceived that heart disease was a result of global warming (45). As this was based on opinions, the link between climate change and heart diseases was unclear. While climate change implications to heart diseases could be mediated, for example, by heatwaves (48), our focus was on those impacts mediated via the wider food systems to DR-NCDs in SSA and we could not find sufficient evidence to include influence on heart disease to the HLPE-SSA adapted framework.

For peer review only - http://bmjopen.bmj.com/site/about/guidelines.xhtml

Food storage pathway

Related to food storage pathway, five studies (38, 41, 42, 46, 47) examined how temperature (related to increased humidity) and rainfall correlate with liver cancer risks through food storage practices. Aflatoxin (AF) contamination was reported in maize (38, 41), small grains (finger millet, pearl millet, sorghum) (38), milk (47), peanuts (46), and *burkina* — a millet-based fermented milk beverage (42).

Liver cancer risk: In Zimbabwe, mycotoxins as a result of increased rainfall, heat and drought were associated with liver cancer (38). The high temperatures were related to humid conditions in West Sudan, which favoured aflatoxin growth in peanuts, in a region reported with high liver cancer incidences (46). A study conducted in four agroecological zones in Kenya found that aflatoxin exposure from milk was associated with minimal incidence of liver cancer [47]. Consumption of small grains (38, 41, 46) and milk consumption posed low liver cancer risks (47), while maize (38) and *burkina* (42) consumption presented high risks. Peanuts also showed an increased risk of liver cancer, particularly in humid conditions (46).

Food consumption pathway

Diabetes risk: One study investigated the impact of rainfall and temperature on food consumption and its correlation with diabetes (43). This study revealed that the highest precipitation level coincided with the peak number of newly diagnosed diabetes cases. At the same time, the hospital admission rates for diabetic patients were slightly higher during the rainy season. The authors attributed this phenomenon to nutritional variability. During the long rainy season, physical activity reduces, and people tend to consume high-fat and high-calorie food while awaiting the harvest. Consequently, increased caloric intake could lead to hyperglycemia and the onset of diabetes symptoms (43).

Grey literature findings

Majority of the reports from the grey literature lacked specificity of region and focus. Among the few reports that explored the relationship between climate change, food systems and DR-NCDs, they did not provide country-specific results (18) or did not explicitly describe which health outcome (49) was being addressed. Although most reports mentioned how climate change impacted food security and malnutrition, the studies did not mention which type of malnutrition (undernutrition or DR-NCDs) it was focusing on (49). Thus, they were not included in this review. We explored a sample of governments but could not find relevant studies to include in this review.

For peer review only - http://bmjopen.bmj.com/site/about/guidelines.xhtml

Table 2: Description of studies included in the review by food system pathway.

Author/ year	Country	Study design	Method of analysis	Study objectives	Sample size	Climate change component	Outcome	Key findings
**Food production pathway**								
Andersen 2021 (39)	Kenya	Qualitative	Descriptive Statistics	To explore knowledge and perspectives on climate change and health-related issues, with a particular focus on noncommunicable diseases	28	Climate change	Diabetes, hypertension, heart attack, cancer, and obesity	Climate change-induced food insecurity and rising food prices were considered as prominent challenges.
Kasomo 2021 (40)	Kenya	Cross-sectional analysis	Inferential Statistics	To quantify spatial variations and estimate the effect of climatic and environmental factors on under- and over-nutrition among women in Kenya	40,300	Temperature and precipitation	Obesity and overweight	Higher precipitation levels were associated with an increased likelihood of women being overweight and obese.
Ngwira 2020 (44)	Malawi	Cross-sectional analysis	Multivariate probit model	To investigate the effects of climate and location using the multivariate model of malnutrition	5,149	Rainfall	Overweight	A positive correlation was observed between rainfall and overweight individuals.
Odonkor & Sallar 2020 (45)	Ghana	Descriptive cross-sectional design	Inferential Statistics	To investigate public knowledge of global warming and its effects on human health	1,130	Global warming	Heart disease	Rising food prices impact the affordability and availability of food in urban areas.
**Food storage pathway**								

For peer review only

For peer review only - http://bmjopen.bmj.com/site/about/guidelines.xhtml

Akello 2021 (38)	Zimbabwe	Cross-sectional analysis	Inferential Statistics	To assess frequencies of mycotoxin-producing fungi in maize, sorghum, pearl millet, and finger millet cropped and traded in Zimbabwe	NI[1]	Drought, long dry spells,	Liver cancer	Consumption of aflatoxin-contaminated maize stored for 4-6 months increased liver cancer rates.
Kimanya 2021 (41)	Tanzania	Cross-sectional analysis	Inferential Statistics	To estimate the population risk of aflatoxin-induced liver cancer	NI[1]	Temperature	Liver cancer	Population risk for aflatoxin-induced liver cancer nationwide was estimated to be 2.95 cases per 100,000 people.
Kortei 2022 (42)	Ghana	Cross-sectional survey	NI	To evaluate AFM_1 levels and cancer risks associated with *burkina* (a millet-based fermented milk product)	150	Temperature	Liver cancer	Consumption of *burkina* showed varied risk levels of liver cancer.
Omer 1998 (46)	Sudan	Cross-sectional survey	Inferential Statistics	To investigate whether aflatoxin contamination of peanut products may contribute to the incidence of hepatocellular carcinoma (HCC) in Sudan	54	Temperature	Liver cancer	Aflatoxin contamination of peanuts has a significant association with liver cancer.
Sirma 2019 (47)	Kenya	Cross-sectional survey	Inferential Statistics	To assess the risk of liver cancer posed by AFM_1 in milk, assuming 10-fold lower carcinogenicity than AFB_1	NI[1]	Rainfall, droughts, temperature	Liver cancer	Aflatoxin exposure from milk contributed a small percentage to liver cancer incidence rates.
**Food consumption pathway**								
Lontchi-Yimagou 2016 (43)	Cameroon	Retrospective study	Inferential Statistics	To investigate the relationship between diabetes hospitalisation rates and climate variations in Yaoundé	818	Temperature and precipitation	Diabetes	Diabetes admissions were higher during rainy seasons due to nutritional variability.

[1] NI-Not indicated

For peer review only - http://bmjopen.bmj.com/site/about/guidelines.xhtml

Conceptual framework of food systems for DR-NCDs and nutrition with evidence drawn from the local context

Figure 3 presents an amended food systems conceptual framework, named here HLPE-SSA, for understanding the pathways and mechanisms in which climate change impacts on food systems and DR-NCDs in SSA. HLPE-SSA is amended from the HLPE framework and is based on the evidence and results discussed above. While both the HLPE and HLPE-SSA show the pathways in which climate change is linked to food systems and DR-NCD outcomes, HLPE-SSA differs slightly from HLPE. *First* is that HLPE-SSA provides a region-specific analysis of how climate change impacts food systems and DR-NCDs. Due to limited evidence from SSA, we expect the HLPE-SSA framework to change with further data from other areas and new research, for example, with respect to heart disease. *Second*, while HLPE shows more generalised linkages between climate change and nutrition outcomes, HLPE-SSA provides a more in-depth synthesis, specifying the mechanisms by which climate change affects food systems and nutrition. For instance, HLPE-SSA shows how temperature can disrupt agricultural production, reducing food availability and subsequent impacts on the risk of diabetes. *Third*, HLPE-SSA proposes a potential pathway—the food consumption pathway—which is not present in the HLPE framework. This pathway acknowledges that diets and unhealthy consumption practices could also be mediated by other factors beyond food production, such as mobility, marketing, retail, and trade policies.

The evidence from HLPE-SSA aligns with four (production, storage, retail, and consumption) of the six hypothesised pathways.

Regarding the ***food production pathway***, HLPE-SSA confirms our hypothesis. Variations in temperature and precipitation influence food production. Consequently, these changes impact on the quality and quantity of food, which were associated with the risk of diabetes, hypertension, heart attack, obesity, and overweight.

For the ***food storage pathway***, HLPE-SSA confirms our hypothesis regarding the effect of long dry spells, drought and fluctuations in rainfall and temperature on food storage and, in turn, liver cancer in SSA. The impact was more pronounced in *poor storage and harvesting conditions* of grains, whereas unfavourable weather conditions, such as high temperatures and rainfall, increased the risk. These weather conditions impacted humidity levels (above 14% moisture content) increasing aflatoxin growth in grains and milk. Aflatoxin exposure in these grains and milk was linked to the risk of liver cancer.

Regarding the ***food retail pathway***, HLPE-SSA suggests that variations in temperature and rainfall patterns affected trade and markets, disrupting transportation, and increasing food prices. This, in turn, increased the cost of food, thereby impacting its affordability and convenience. These changes in the food systems were associated with an increased risk of diabetes, hypertension, heart attack, obesity, and overweight.

Regarding the ***food consumption pathway***, HLPE-SSA supported our hypothesis that temperature and rainfall patterns alter eating patterns and physical activity during the long rains. This resulted in reduced food quality and nutrient density, which was associated with high diabetes risk.

For peer review only - http://bmjopen.bmj.com/site/about/guidelines.xhtml

437 Lastly, HLPE-SSA framework highlights research gaps related to the *food distribution, market* and *food processing and packaging pathways*. Although we hypothesised that extreme weather events might disrupt the distribution and processing of food in SSA —and consequently on DR-NCDs. To the best of our knowledge, no studies investigated how climatic impacts can influence DR-NCDs through these two pathways.

For peer review only

For peer review only - http://bmjopen.bmj.com/site/about/guidelines.xhtml

Discussion

This scoping review focused on studies on the SSA context to better understand the relevance of local context to food systems and DR-NCDs. It indicates very limited research focus and calls for further research on food systems and DR-NCDs in SSA. Despite this, findings from our review supported our hypotheses that climate change influences food systems through food production, food storage, food retail, and food consumption pathways. These changes in the food chains compromise food quality, safety, quantity, and nutrient density. These factors contribute to a shift towards less nutritious and more processed foods associated with developing DR-NCDs.

Food storage pathway focuses on how climate-induced changes in rainfall and precipitation affect food harvest and storage conditions, leading to the growth of Aflatoxin B_1 and Aflatoxin M_1 (AFM_1) – a type of mycotoxin produced by fungi *Aspergillus flavus* and *Aspergillus parasiticus* – present in grains, maize, nuts, peanuts, flour, and milk (50). The review's findings are consistent with other research conducted in Bangladesh, which reported a significant proportion of liver cancer cases attributed to dietary aflatoxin exposure from maize and other crops in warm and humid conditions (51). Notably, based on the findings of this review, the risk of aflatoxin contamination and its associated liver cancer risk was found to present a lower risk in milk and other grains. This observation is in line with studies by Saha Turna et al. (52) conducted in Bangladesh and globally (51), where aflatoxin exposure in milk and grains such as wheat and rice were not associated with liver cancer. Several factors are identified as influencing aflatoxin contamination, including storage conditions, duration, and type of crop storage structure. Aflatoxins are prone to develop during food production in dry and humid conditions, whereas wet and humid conditions during harvesting and storage create conducive conditions for the growth of aflatoxins (53).

Related to *food production pathway,* evidence from Cameroon, Kenya, and Malawi showed how climate variables correlate with diabetes, heart disease, and obesity. Seasonal changes in rainfall were found to influence physical activity, eating habits and dietary choices, and in turn diabetes and obesity in the region. The review's findings align with other studies that have linked disruptions in agricultural production resulting from extreme weather events with diabetes and obesity (54). During rainy seasons and extreme weather events, agricultural production activities are disrupted, leading to food shortages (42). During food shortages, individuals with diabetes may not consume sufficient fruit and vegetables, and compensate with increased energy-dense, high-fat/salt foods, worsening their condition (42). Another plausible reason could be that people would be more likely to seek care during the rainy season as they are not busy with farm activities.

The increased access to and consumption of processed and unhealthy foods may contribute to the burden of DR-NCDs. *Food consumption pathway* is supported by a review (27) highlighting that extreme weather events are likely to become more frequent determinants of food purchase and consumption. These events can have a dual impact, either by restricting consumers' access to food or by shaping their food preferences based on the availability of certain product (27).

For peer review only - http://bmjopen.bmj.com/site/about/guidelines.xhtml

Evidence from SSA supported four of the hypothesised pathways. Studies in this review did not support the *food distribution, market* and *processing and packaging* pathways. This could be attributed to the limited evidence and studies included in the review and hence could not support all the pathways.

In summary, the HLPE-SSA framework is based on very limited research, but it provides a structured approach to understanding the complex interactions between climate change and related systemic risk, food systems, and DR-NCDs in SSA. It is important that our amendments to the HLPE framework are considered as the first exploratory findings calling for further research from SSA in the area for more comprehensive context-based understanding.

Strengths

This review exhibits several notable strengths.

First, the HLPE framework has been extensively used by other researchers (55, 56), which strengthens the methodology of this review.

Second, this review used an established food systems framework to guide the methodology and research process. This provided a clear structure to clarify pathways on the causal relationship between climate change, food systems, and DR-NCDs.

Third, the review goes beyond existing frameworks by proposing a context-specific framework tailored to SSA. Similar frameworks for food systems exist, but they are extensive in scope. HLPE-SSA, offers an in-depth and specific synthesis of how climate change and certain systemic drivers influence SSA's food systems and contribute to the development of DR-NCDs. This approach enhances the framework's applicability in guiding targeted interventions such as better crop storage, sustainable agriculture, and policies that address the region's specific concerns.

Lastly, the review adhered to a systematic approach, following JBI guidance for conducting scoping reviews. This systematic process ensures the reliability and comprehensiveness of the research process.

Limitations

One key limitation of the review is the possibility of publication bias, as the review focused on international organisations and academic publications. Relevant studies published in country-based databases as well as local languages, might have been missed. However, our search of a sample of government websites did not indicate that we missed substantial body of literature. In addition, even though we did not restrict our search to English, most articles retrieved from our search were in English and thus this might have biased the results.

Additionally, the nature of the studies included in the review – mainly observational and descriptive – limits the establishment of definitive causal relationships between climate change, food systems, and DR-NCDs. Although they offered insights and generated hypotheses of the potential linkages, they may not provide conclusive evidence on causality. Further research, including natural experiments, longitudinal, and time series analysis studies, would be necessary to establish more causal links. This research would also need to focus on the

For peer review only

For peer review only - http://bmjopen.bmj.com/site/about/guidelines.xhtml

proportional role of different pathways of food systems in relation to DR-NCDs in changing societies, dietary, and consumption practices in SSA.

Some studies, mainly qualitative studies, looked at the linkages between climate change and DR-NCDs without showing how they influence each other. Therefore, we had to assemble the scattered evidence from these studies to form a comprehendible picture of these linkages. However, these linkages were not included in the HLPE-SSA framework.

Furthermore, this review did not include studies exploring the potential effects of floods and famine on DR-NCDs. Floods can impact food security by destroying crops and sometimes leading to famine. Floods also destroy the socioeconomic activities linked to food production, which could impact food accessibility, affordability, and utilization. On the other hand, empirical studies have shown that famine exposure in the first 1000 days has been associated with higher risk of diabetes in adulthood (57, 58) (foetal famine), which represents another area that could be relevant to the overall understanding of climate change impacts on DR-NCDs in SSA. Including such evidence in future research could contribute to a more comprehensive analysis of the factors influencing DR-NCDs in the region.

Conclusion

In conclusion, this review suggests climate change can significantly affect food systems, nutrition, and DR-NCDs in SSA. It highlights the association between aflatoxin-contaminated food and liver cancer in multiple countries. It also underscores how seasonal variations in rainfall can influence physical activity and dietary choices, potentially exacerbating diabetes. Furthermore, extreme weather events are identified as influential factors shaping food purchase and consumption patterns, potentially limiting access to nutritious foods and influencing food preferences.

Our work on the HLPE-SSA suggests that while wider conceptual frameworks such as HLPE work as a general reference, it is important for research and practice to case light also to regional adjustments of more global frameworks. This review further highlights the key areas for further work such as the mechanism in which climate change influence heart diseases and the gaps related the *food distribution, market* and *processing and packaging* pathways.

Ethical approval

Ethics approval is not required as this is a scoping review, and data included in this review are from publicly available documents.

For peer review only - http://bmjopen.bmj.com/site/about/guidelines.xhtml

References

1. World Health Organization. Noncommunicable diseases: Key facts. 2022.
2. Gouda HN, Charlson F, Sorsdahl K, Ahmadzada S, Ferrari AJ, Erskine H, et al. Burden of non-communicable diseases in sub-Saharan Africa, 1990–2017: results from the Global Burden of Disease Study 2017. The Lancet Global Health. 2019;7(10):e1375-e87.
3. World Health Organization. Sustainable Healthy Diets – Guiding Principles. Rome: World Health Organization; 2019 [
4. Pingault N, Caron P, Kalafatic C, Allahoury A, Fresco LO, Kennedy E, et al. Nutrition and food systems. A report by the High Level Panel of Experts on Food Security and Nutrition of the Committee on World Food Security. 2017.
5. Fanzo J, Davis C. Global food systems, diets, and nutrition : linking science, economics, and policy. Springer Nature. 2021.
6. HLPE. Food security and nutrition: building a global narrative towards 2030. Report by the High Level Panel of Experts on Food Security and Nutrition of the Committee on World Food Security, Rome. 2020.
7. Turner C, Aggarwal A, Walls H, Herforth A, Drewnowski A, Coates J, et al. Concepts and critical perspectives for food environment research: a global framework with implications for action in low-and middle-income countries. Global food security. 2018;18:93-101.
8. Frumkin H, Haines A. Global Environmental Change and Noncommunicable Disease Risks. Annual Review of Public Health. 2019;40:261-82.
9. Pachauri RK, Allen MR, Barros VR, Broome J, Cramer W, Christ R, et al. Climate change 2014: synthesis report. Contribution of Working Groups I, II and III to the fifth assessment report of the Intergovernmental Panel on Climate Change: Ipcc; 2014.
10. Fanzo J, Davis C, McLaren R, Choufani J. The effect of climate change across food systems: Implications for nutrition outcomes. Global Food Security. 2018;18:12-9.
11. Trudinger H, Crimp S, Friedman RS. Food systems in the face of climate change: reviewing the state of research in South Pacific Islands. Regional Environmental Change. 2023;23(1):45-.
12. Stefan DC, Elzawawy AM, Khaled HM, Ntaganda F, Asiimwe A, Addai BW, et al. Developing cancer control plans in Africa: examples from five countries. The lancet oncology. 2013;14(4):e189-e95.
13. Hunter-Adams J, Battersby J. Health care providers' perspectives of diet-related non-communicable disease in South Africa. BMC Public Health. 2020;20(1).
14. Kassa MD, Grace JM. Noncommunicable diseases prevention policies and their implementation in Africa: a systematic review. Public Health Reviews. 2022;42:1604310.
15. Hawkes C, Parsons K. Tackling Food Systems Challenges: The Role of Food Policy. London, United Kingdom. https://doi. org/10.2307/j. ctvr695rp; 2019.
16. World Health Organization. Systems thinking for noncommunicable disease prevention policy: guidance to bring systems approaches into practice. World Health Organization. Regional Office for Europe; 2022.
17. Dalal S, Beunza JJ, Volmink J, Adebamowo C, Bajunirwe F, Njelekela M, et al. Non-communicable diseases in sub-Saharan Africa: what we know now. International journal of epidemiology. 2011;40(4):885-901.
18. HLPE. Nutrition and food systems. A report by the High Level Panel of Experts on Food Security and Nutrition of the Committee on World Food Security, Rome. 2017.
19. High Level Panel of Experts. About the HLPE-FSN [Available from: https://www.fao.org/cfs/cfs-hlpe/about/mission/en.

For peer review only - http://bmjopen.bmj.com/site/about/guidelines.xhtml

20. Swinburn BA, Kraak VI, Allender S, Atkins VJ, Baker PI, Bogard JR, et al. The global syndemic of obesity, undernutrition, and climate change: the Lancet Commission report. The lancet. 2019;393(10173):791-846.

21. Devereux S. The impact of droughts and floods on food security and policy options to alleviate negative effects. Agricultural Economics. 2007;37:47-58.

22. Fanzo J, Miachon L. Harnessing the connectivity of climate change, food systems and diets: Taking action to improve human and planetary health. Anthropocene. 2023;42:100381-.

23. Scott-Villiers P, Chisholm N, Wanjiku Kelbert A, Hossain N. Precarious lives: work, food and care after the global food crisis. 2016.

24. Springmann M, Mason-D'Croz D, Robinson S, Garnett T, Godfray HCJ, Gollin D, et al. Global and regional health effects of future food production under climate change: a modelling study. The Lancet. 2016;387(10031):1937-46.

25. Thornton PK, Ericksen PJ, Herrero M, Challinor AJ. Climate variability and vulnerability to climate change: a review. Global change biology. 2014;20(11):3313-28.

26. World Health Organization (International Agency for Research on cancer). IARC Monographs on the evaluation of carcinogenic risks to Humans. Some Traditional Herbal Medicines, Some Mycotoxins, Naphthalene and Styrene IARC Monographs on the Evaluation of Carcinogenic Risks to Humans2002.

27. Davis KF, Downs S, Gephart JA. Towards food supply chain resilience to environmental shocks. Nature Food. 2021;2(1):54-65.

28. Peters MDJ, Marnie C, Tricco AC, Pollock D, Munn Z, Alexander L, et al. Updated methodological guidance for the conduct of scoping reviews. JBI Evidence Synthesis. 2020;18(10).

29. Page MJ, McKenzie JE, Bossuyt PM, Boutron I, Hoffmann TC, Mulrow CD, et al. The PRISMA 2020 statement: an updated guideline for reporting systematic reviews. Systematic Reviews. 2021;10(1):1-11.

30. World Bank. Data [Available from: https://data.worldbank.org/region/sub-saharan-africa.

31. L. A G. United Nations Framework Convention on Climate Change. Environ Health Perspect. 2000;108:A353-A.

32. Race to Zero. From 1800 to COP26: A history of key climate conversations. 2021.

33. Haddaway NR, Collins AM, Coughlin D, Kirk S. The Role of Google Scholar in Evidence Reviews and Its Applicability to Grey Literature Searching. PLOS ONE. 2015;10(9):e0138237.

34. Reliefweb. All disasters. Alert/Ongoing [Available from: https://reliefweb.int/disasters?view=ongoing.

35. World Bank. World Bank Country and Lending Groups 2024 [Available from: https://datahelpdesk.worldbank.org/knowledgebase/articles/906519-world-bank-country-and-lending-groups#:~:text=For%20the%20current%202024%20fiscal,those%20with%20a%20GNI%20per.

36. Ouzzani M, Hammady H, Fedorowicz Z, Elmagarmid A. Rayyan—a web and mobile app for systematic reviews. Systematic reviews. 2016;5:1-10.

37. Noyes J, Booth A, Moore G, Flemming K, Tunçalp Ö, Shakibazadeh E. Synthesising quantitative and qualitative evidence to inform guidelines on complex interventions: clarifying the purposes, designs and outlining some methods. BMJ global health. 2019;4(Suppl 1):e000893-e.

For peer review only - http://bmjopen.bmj.com/site/about/guidelines.xhtml

38. Akello J, Ortega-Beltran A, Katati B, Atehnkeng J, Augusto J, Mwila CM, et al. Prevalence of Aflatoxin- and Fumonisin-Producing Fungi Associated with Cereal Crops Grown in Zimbabwe and Their Associated Risks in a Climate Change Scenario. Foods 2021, Vol 10, Page 287. 2021;10(2):287-.

39. Greibe Andersen J, Karekezi C, Ali Z, Yonga G, Kallestrup P, Kraef C. Perspectives of local community leaders, health care workers, volunteers, policy makers and academia on climate change related health risks in Mukuru informal settlement in Nairobi, Kenya—a qualitative study. International Journal of Environmental Research and Public Health. 2021;18(22):12241-.

40. Kasomo JM, Gayawan E. Spatial location, temperature and rainfall diversity affect the double burden of malnutrition among women in Kenya. SSM - Population Health. 2021;16:100939-.

41. Kimanya ME, Michael NR, Emmanuel M, Chibundu NE, Candida PS, Yun YG. Estimating the risk of aflatoxin-induced liver cancer in Tanzania based on biomarker data. PLoS ONE. 2021;16(3).

42. Kortei NK, Annan T, Boakye AA, Essuman EK, Tettey CO, Kyei-Baffour V. Aflatoxin M1 exposure in a fermented millet-based milk beverage 'brukina'and its cancer risk characterization in Greater Accra, Ghana. Scientific Reports. 2022;12(1):12562-.

43. Lontchi-Yimagou E, Tsalefac M, Tapinmene LMT, Noubiap JJN, Balti EV, Nguewa JL, et al. Seasonality in diabetes in Yaounde, Cameroon: a relation with precipitation and temperature. BMC public health. 2016;16(1).

44. Ngwira A. Climate and location as determinants of childhood stunting, wasting, and overweight: an application of semiparametric multivariate probit model. Nutrition. 2020;70:100010-.

45. Odonkor ST, Sallar AM. An Assessment of Public Knowledge and Potential Health Impacts of Global Warming in Ghana. BioMed Research International. 2020;2020.

46. Omer RE, Bakker MI, van P, A P Hoogenboom RL, G Polman TH, Alink GM, et al. Aflatoxin and liver cancer in Sudan. NUTRITION AND CANCER. 1998;32(3):174-80.

47. Sirma AJ, Makita K, Randolph DG, Senerwa D, Lindahl JF. Aflatoxin Exposure from Milk in Rural Kenya and the Contribution to the Risk of Liver Cancer. Toxins 2019, Vol 11, Page 469. 2019;11(8):469-.

48. Liu J, Varghese BM, Hansen A, Zhang Y, Driscoll T, Morgan G, et al. Heat exposure and cardiovascular health outcomes: a systematic review and meta-analysis. The Lancet Planetary Health. 2022;6(6):e484-e95.

49. AfDB. Economic Brief - Health in Africa Over the Next 50 Years. 2013.

50. Bbosa GS, Kitya D, Lubega A, Ogwal-Okeng J, Anokbonggo WW, Kyegombe DB. Review of the biological and health effects of aflatoxins on body organs and body systems. Aflatoxins-recent advances and future prospects. 2013;12:239-65.

51. Saha Turna N, Wu F. Risk assessment of aflatoxin-related liver cancer in Bangladesh. Food Additives & Contaminants: Part A. 2019;36(2):320-6.

52. Saha Turna N, Havelaar A, Adesogan A, Wu F. Aflatoxin M1 in milk does not contribute substantially to global liver cancer incidence. The American Journal of Clinical Nutrition. 2022;115(6):1473-80.

53. IARC. Monographs on the evaluation of carcinogenic risks to humans: Overall evaluations of carcinogenicity: An updating of IARC Monographs. Lyon, France. International Agency for Research on Cancer. 1987:1-403.

54. Zilbermint M. Diabetes and climate change. Journal of Community Hospital Internal Medicine Perspectives. 2020;10(5):409-12.

For peer review only - http://bmjopen.bmj.com/site/about/guidelines.xhtml

55. Harris J, Tan W, Raneri JE, Schreinemachers P, Herforth A. Vegetables for healthy diets in low-and middle-income countries: a scoping review of the food systems literature. Food and Nutrition Bulletin. 2022;43(2):232-48.
56. Storhaug IG, Lane C, Moore N, Engelbert M, Sparling TM, Franich A, et al. Making the most of existing research: an evidence gap map of the effects of food systems interventions in low-income and middle-income countries. BMJ open. 2022;12(6):e055062.
57. Kraemer K, Cordaro J, Fanzo J, Gibney M, Kennedy E, Labrique A, et al. Diet and non-communicable diseases: an urgent need for new paradigms. Good nutrition: Perspectives for the 21st century: Karger Publishers; 2016. p. 105-18.
58. Nyirenda MJ. Non-communicable diseases in sub-Saharan Africa: understanding the drivers of the epidemic to inform intervention strategies. International health. 2016;8(3):157-8.

For peer review only

For peer review only - http://bmjopen.bmj.com/site/about/guidelines.xhtml

Funding statement

JT was supported by the EDUFI Fellowship (Finnish National Agency for Education). SH was funded by the Medical Research Council grant number MR/S009035/1. MK, JP, and SA have not declared grant from any funding agency in the public, commercial or not-for-profit sectors.

Risk and quality assessment

As this is a scoping review, no risk or quality assessment was conducted.

Data availability statement

There are not any more data available. All data relevant to this review, have been included in the article or uploaded online supplemental information.

Contributions

JT, SH, JP, and MK conceptualised the article. JT and MK conducted the screening of studies. JT did data extraction. JT, SA, JP, SH, and MK contributed to the study methodology. JT, SH, MK, SA, and JP contributed to writing – review and editing. JT contributed to writing – original draft.

Conflict of interest

The authors declare no conflict of interest.

Figures

Figure 1: "HLPE. 2017. Nutrition and food systems. A report by the High Level Panel of Experts on Food Security and Nutrition of the Committee on World Food Security, Rome." This is the original HLPE framework. The food supply chains in this framework are henceforth referred to as referred to as pathways in this review.

Figure 2: PRISMA Flow Diagram: This diagram shows the systematic process we followed to include articles by our search framework.

Figure 3: Food systems conceptual framework for Sub-Saharan Africa. HLPE-SSA. Figure adapted from Figure 1 in HLPE. 2017. Nutrition and food systems. A report by the High Level Panel of Experts on Food Security and Nutrition of the Committee on World Food Security, Rome." Arrows show the expected causal effect.

For peer review only

For peer review only - http://bmjopen.bmj.com/site/about/guidelines.xhtml

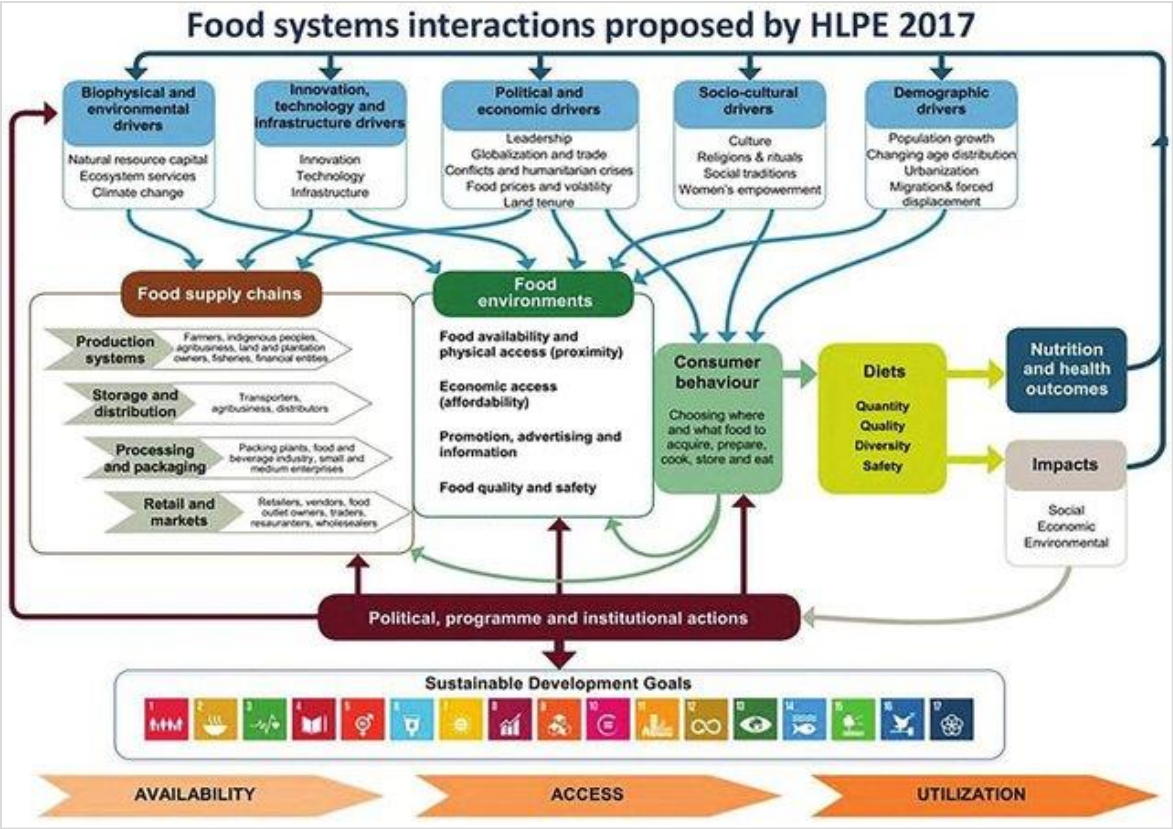
Food systems interactions proposed by HLPE 2017
Biophysical and environmental drivers
Natural resource capital
Ecosystem services
Climate change
Innovation, technology and infrastructure drivers
Innovation
Technology
Infrastructure
Political and economic drivers
Leadership
Globalization and trade
Conflicts and humanitarian crises
Food prices and volatility
Land tenure
Socio-cultural drivers
Culture
Religions & rituals
Social traditions
Women's empowerment
Demographic drivers
Population growth
Changing age distribution
Urbanization
Migration& forced displacement
Food supply chains
Production systems
Storage and distribution
Processing and packaging
Retail and markets
Food environments
Food availability and physical access (proximity)
Economic access (affordability)
Promotion, advertising and information
Food quality and safety
Consumer behaviour
Choosing where and what food to acquire, prepare, cook, store and eat
Diets
Quantity
Quality
Diversity
Safety
Nutrition and health outcomes
Impacts
Social
Economic
Environmental
Political, programme and institutional actions
Sustainable Development Goals
AVAILABILITY
ACCESS
UTILIZATION

review only

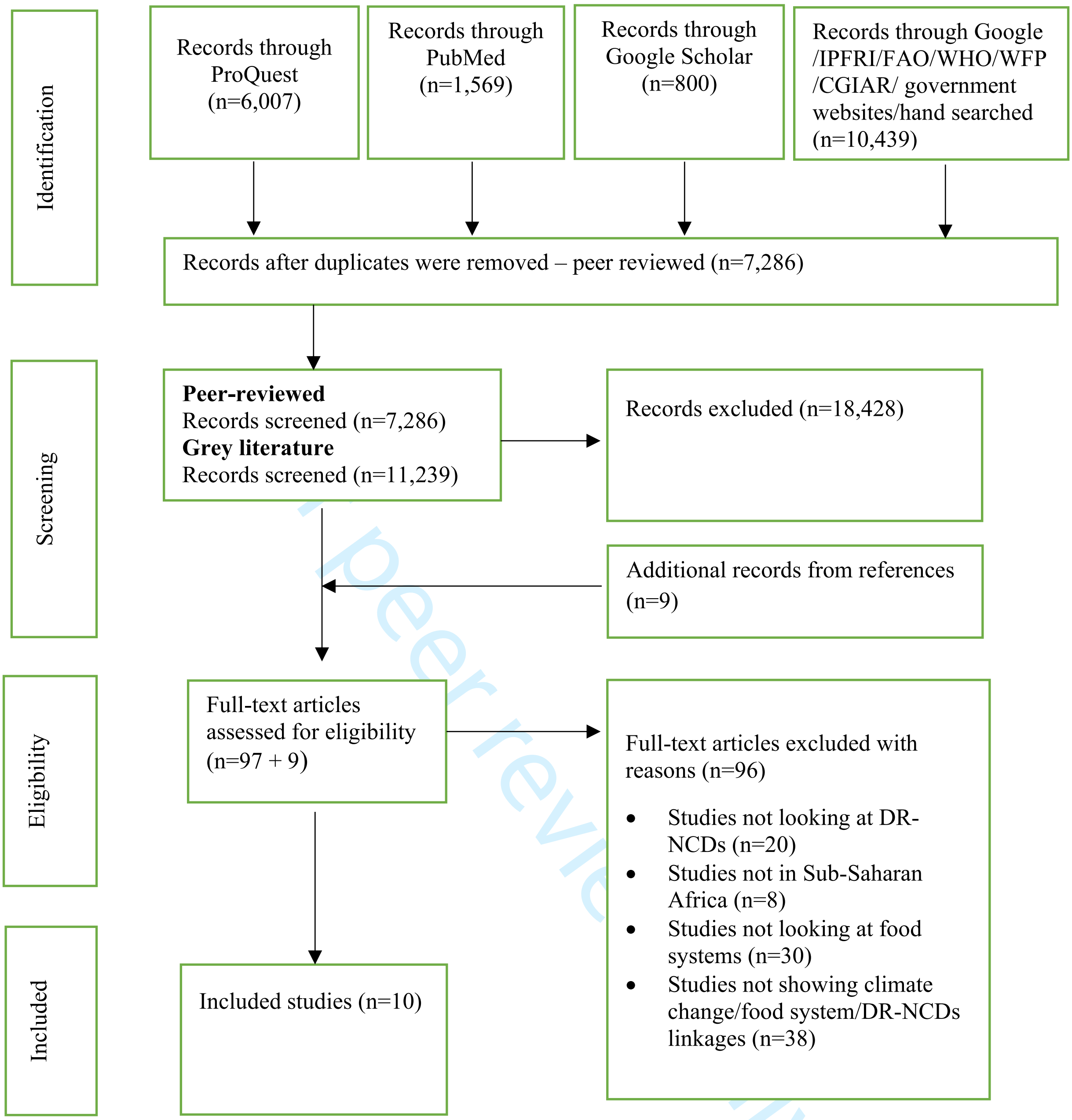
Identification
Records through ProQuest (n=6,007)
Records through PubMed (n=1,569)
Records through Google Scholar (n=800)
Records through Google /IPFRI/FAO/WHO/WFP /CGIAR/ government websites/hand searched (n=10,439)
Records after duplicates were removed – peer reviewed (n=7,286)
Screening
Peer-reviewed
Records screened (n=7,286)
Grey literature
Records screened (n=11,239)
Records excluded (n=18,428)
Additional records from references (n=9)
Eligibility
Full-text articles assessed for eligibility (n=97 + 9)
Full-text articles excluded with reasons (n=96)
Studies not looking at DR-NCDs (n=20)
Studies not in Sub-Saharan Africa (n=8)
Studies not looking at food systems (n=30)
Studies not showing climate change/food system/DR-NCDs linkages (n=38)
Included
Included studies (n=10)

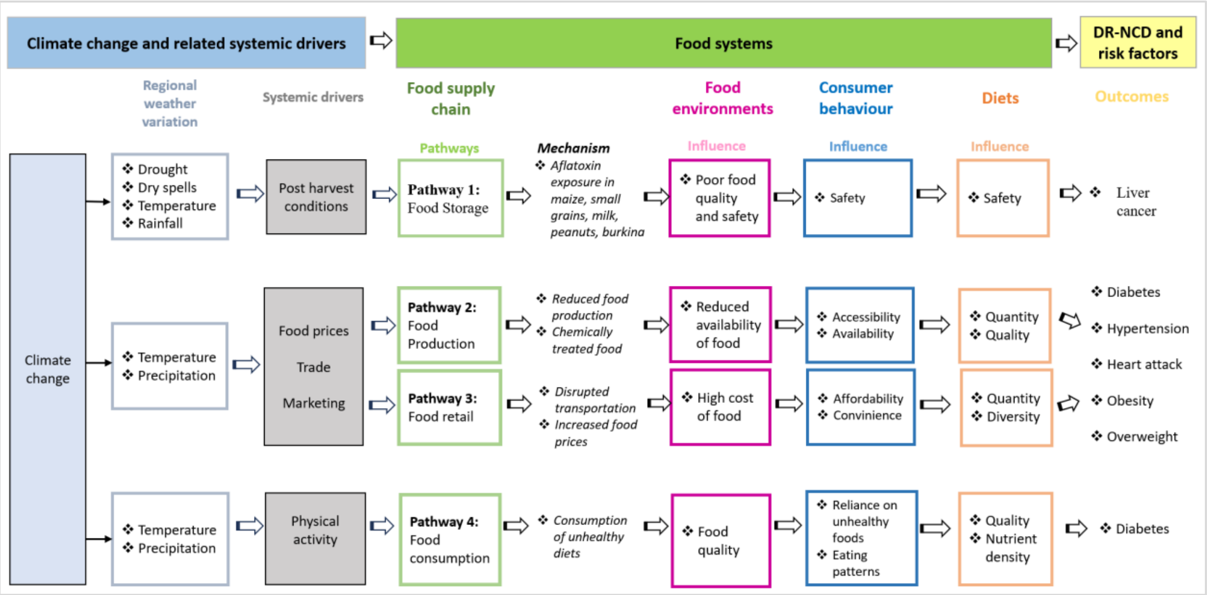

For peer review only

Appendix 1

Search terms-Academic databases

"Sub-Saharan Africa" OR Angola OR Benin OR Botswana OR Burkina Faso OR Burundi OR "Cabo Verde" OR Cameroon OR "Central African Republic" OR Chad OR Comoros OR "Democratic Republic of Congo" OR "Republic of Congo" OR "Cote d'Ivoire" OR "Equatorial Guinea" OR Eritrea OR Eswatini OR Swaziland OR Ethiopia OR Gabon OR Gambia OR Ghana OR Guinea OR Guinea-Bissau OR Kenya OR Lesotho OR Liberia OR Madagascar OR Malawi OR Mali OR Mauritania OR Mauritius OR Mozambique OR Namibia OR Niger OR Nigeria OR Rwanda OR "Sao Tome and Principe" OR Senegal OR Seychelles OR "Sierra Leone" OR Somalia OR "South Africa" OR "South Sudan" OR Sudan OR Tanzania OR Togo OR Uganda OR Zambia OR Zimbabwe AND "Noncommunicable Diseases"[Mesh] OR "Noncommunicable Disease*" [tw] OR "Non-communicable Diseases" [tw] OR "Non communicable Diseases" [tw] OR "Non-communicable Chronic Diseases" [tw] OR "Non-communicable Chronic Disease*" [tw] OR "Non-infectious Diseases" [tw] OR "Chronic diseases" [tw] OR diabetes*[tw] OR DM* OR T2DM* OR overweight OR Hypertension*[tw] OR "high blood pressure"[tw] OR obesity*[tw] OR cholestrol* [tw] OR cancer*[tw] OR "Chronic respiratory diseases" AND "Climate Change"[Mesh] OR "Climate Changes*" [tw] OR "Climate change*" [tw] OR "climate impacts" OR "greenhouse gas" [tw] OR floods*[tw] OR drought*[tw] OR "heat waves*" [tw] OR greenhouse*[tw] OR "global warming"[tw] OR famine*[tw] OR temperature[tw] OR "extreme weather*"[tw] OR humidity OR precipitation OR "water-related"

For peer review only

Appendix 2

Search terms-Grey literature

Google Scholar and Google

Sub-Saharan Africa AND Noncommunicable Diseases OR Noncommunicable Disease OR Non-communicable Diseases OR Non communicable Diseases OR Non-communicable Chronic Diseases OR Non-communicable Chronic Disease OR Non-infectious Diseases OR Chronic diseases OR diabetes OR DM OR T2DM OR overweight OR Hypertension OR high blood pressure OR obesity OR cholesterol OR cancer OR Chronic respiratory diseases AND Climate Change OR Climate Changes OR Climate change OR climate impacts OR greenhouse gas OR floods OR drought OR heat waves OR greenhouse OR global warming OR famine OR temperature OR extreme weather OR humidity OR precipitation OR water-related

FAO

Climate change OR heat OR famine OR floods AND diabetes OR NCDs OR noncommunicable diseases OR Obesity OR cancer OR cardiovascular diseases AND Sub-Saharan Africa OR Republic of Congo OR Cote d'Ivoire OR Equatorial Guinea OR Eritrea OR Eswatini OR Swaziland OR Ethiopia OR Gabon OR Gambia OR Ghana OR Guinea OR Guinea-Bissau OR Kenya OR Lesotho OR Liberia OR Madagascar OR Malawi OR Mali OR Mauritania OR Mauritius OR Mozambique OR Namibia OR Sub-Saharan Africa OR Angola OR Benin OR Botswana OR Burkina Faso OR Burundi OR Cabo Verde OR Cameroon OR Central African Republic OR Chad OR Comoros OR Democratic Republic of Congo OR Niger OR Nigeria OR Rwanda OR Sao Tome and Principe OR Senegal OR Seychelles OR Sierra Leone OR Somalia OR South Africa OR South Sudan" OR Sudan OR Tanzania OR Togo OR Uganda OR Zambia OR Zimbabwe

WHO, WFP and World Bank

Climate change OR heat OR famine OR floods AND diabetes OR noncommunicable diseases OR NCDs OR Obesity OR cancer OR cardiovascular diseases AND Sub-Saharan

IPFRI, CGIAR

Search terms were not used in IPFRI and CGIAR websites, but hand searches were used to search studies under the publications and tools tab. This is because the search terms were bringing null results.

Government websites

Climate change OR heat OR famine OR floods AND diabetes OR NCDs OR noncommunicable diseases OR obesity OR cancer OR cardiovascular diseases AND Sub-Saharan Africa

No search limits used.

For peer review only

Appendix 3: Extraction sheet

Author	Year	Study type	Aim of the study	Country of study	Climate change components discussed	Food systems pathway	Outcomes	Results on the effects of climate change on the outcomes	Causal or hypothetical association

For peer review only

Supplementary Table 1
List of Sub-Saharan Africa countries

No	Country	Population (World Bank 2022)	GDP per capita (PPP) ($) (World Bank 2022)	World Bank 2023 Income Classification
1	Angola	35,588,987	6,976.0	Lower middle income
2	Benin	13,352.86	4,057.5	Lower middle income
3	Botswana	2,384,246	18,329.8	Upper middle income
4	Burkina Faso	22,673,762	2,549.9	Low income
5	Burundi	12,889,576	836.5	Low income
6	Cabo Verde	593,149	8,716.0	Lower middle income
7	Cameroon	27,914,536	4,398.0	Lower middle income
8	Central African Republic	5,579,144	973.2	Low income
9	Chad	17,723,315	1,668.6	Low income
10	Comoros	836,774	3,833.7	Lower middle income
11	Congo, Democratic Republic	99,010,212	1,337.8	Low income
12	Congo Republic	5,970,424	4,335.	Lower middle income
13	Côte d'ivoire	28,160,542	6,540.5	Lower middle income
14	Equatorial Guinea	1,674,908	17,620.7	Upper middle income
15	Eritrea	3,684,032	1,628.8	Low income
16	Eswatini	1,201,670	10,699.5	Lower middle income
17	Ethiopia	123,379,924	2,812.5	Low income
18	Gabon	2,388,992	16,465.0	Upper middle income
19	The Gambia	2,705,992	2,496.5	Low income
20	Ghana	33,475,870	6,473.1	Lower middle income
21	Guinea	13,859,341	3,188.1	Lower middle income
22	Guinea-Bissau	2,105,566	2,191.2	Low income
23	Kenya	54,027,487	5,765.8	Lower middle income
24	Lesotho	2,305,825	2,646.2	Lower middle income
25	Liberia	5,302,681	1,570.5	Low income
26	Madagascar	29,611,714	1,774.7	Low income
27	Malawi	20,405,317	1,732.6	Low income
28	Mali	22,593,590	2,518.9	Low income
29	Mauritania	4,736,139	6,295.8	Lower middle income
30	Mauritius	1,262,523	26,979.1	Upper middle income
31	Mozambique	32,969,518	1,477.3	Low income
32	Namibia	2,567,012	11,531.3	Upper middle income
33	Niger	26,207,977	1,505.5	Low income
34	Nigeria	218,541,212	5,862.2	Lower middle income
35	Rwanda	13,776,698	2,793.2	Low income
36	Sao Tome and Principe	227,38	4,061.8	Lower middle income
37	Senegal	17,316,449	4,210.4	Lower middle income
38	Seychelles	119,878	29,771.7	High income

For peer review only

39	Sierra Leone	8,605,718	1,930.9	Low income
40	Somalia	17,597,511	1,711.0	Low income
41	South Africa	59,893,885	15,920.4	Upper middle income
42	South Sudan	10,913,164	1,181.9	Low income
43	Sudan	46,874,204	4,217.4	Low income
44	Tanzania	65,497,748	3,099.2	Lower middle income
45	Togo	8,848,699	2,601.8	Low income
46	Uganda	47,249,585	2,693.1	Low income
47	Zambia	20,017,675	3,975.6	Lower middle income
48	Zimbabwe	16,320,537	2,607.9	Lower middle income

Data retrieved from the World Bank website.

For peer review only

Preferred Reporting Items for Systematic reviews and Meta-Analyses extension for Scoping Reviews (PRISMA-ScR) Checklist

SECTION	ITEM	PRISMA-ScR CHECKLIST ITEM	REPORTED ON PAGE #
**TITLE**			
Title	1	Identify the report as a scoping review.	1
**ABSTRACT**			
Structured summary	2	Provide a structured summary that includes (as applicable): background, objectives, eligibility criteria, sources of evidence, charting methods, results, and conclusions that relate to the review questions and objectives.	2
**INTRODUCTION**			
Rationale	3	Describe the rationale for the review in the context of what is already known. Explain why the review questions/objectives lend themselves to a scoping review approach.	4-6
Objectives	4	Provide an explicit statement of the questions and objectives being addressed with reference to their key elements (e.g., population or participants, concepts, and context) or other relevant key elements used to conceptualize the review questions and/or objectives.	6
**METHODS**			
Protocol and registration	5	Indicate whether a review protocol exists; state if and where it can be accessed (e.g., a Web address); and if available, provide registration information, including the registration number.	NA
Eligibility criteria	6	Specify characteristics of the sources of evidence used as eligibility criteria (e.g., years considered, language, and publication status), and provide a rationale.	8-9
Information sources*	7	Describe all information sources in the search (e.g., databases with dates of coverage and contact with authors to identify additional sources), as well as the date the most recent search was executed.	9
Search	8	Present the full electronic search strategy for at least 1 database, including any limits used, such that it could be repeated.	9
Selection of sources of evidence†	9	State the process for selecting sources of evidence (i.e., screening and eligibility) included in the scoping review.	10
Data charting process‡	10	Describe the methods of charting data from the included sources of evidence (e.g., calibrated forms or forms that have been tested by the team before their use, and whether data charting was done independently or in duplicate) and any processes for obtaining and confirming data from investigators.	10
Data items	11	List and define all variables for which data were sought and any assumptions and simplifications made.	9
Critical appraisal of individual sources of evidence§	12	If done, provide a rationale for conducting a critical appraisal of included sources of evidence; describe the methods used and how this information was used in any data synthesis (if appropriate).	NA
Synthesis of results	13	Describe the methods of handling and summarizing the data that were charted.	10

For peer review only - http://bmjopen.bmj.com/site/about/guidelines.xhtml

SECTION	ITEM	PRISMA-ScR CHECKLIST ITEM	REPORTED ON PAGE #
**RESULTS**			
Selection of sources of evidence	14	Give numbers of sources of evidence screened, assessed for eligibility, and included in the review, with reasons for exclusions at each stage, ideally using a flow diagram.	10
Characteristics of sources of evidence	15	For each source of evidence, present characteristics for which data were charted and provide the citations.	11
Critical appraisal within sources of evidence	16	If done, present data on critical appraisal of included sources of evidence (see item 12).	NA
Results of individual sources of evidence	17	For each included source of evidence, present the relevant data that were charted that relate to the review questions and objectives.	10
Synthesis of results	18	Summarize and/or present the charting results as they relate to the review questions and objectives.	11-17
**DISCUSSION**			
Summary of evidence	19	Summarize the main results (including an overview of concepts, themes, and types of evidence available), link to the review questions and objectives, and consider the relevance to key groups.	18
Limitations	20	Discuss the limitations of the scoping review process.	19
Conclusions	21	Provide a general interpretation of the results with respect to the review questions and objectives, as well as potential implications and/or next steps.	20
**FUNDING**			
Funding	22	Describe sources of funding for the included sources of evidence, as well as sources of funding for the scoping review. Describe the role of the funders of the scoping review.	25

JBI = Joanna Briggs Institute; PRISMA-ScR = Preferred Reporting Items for Systematic reviews and Meta-Analyses extension for Scoping Reviews.

* Where *sources of evidence* (see second footnote) are compiled from, such as bibliographic databases, social media platforms, and Web sites.

† A more inclusive/heterogeneous term used to account for the different types of evidence or data sources (e.g., quantitative and/or qualitative research, expert opinion, and policy documents) that may be eligible in a scoping review as opposed to only studies. This is not to be confused with *information sources* (see first footnote).

‡ The frameworks by Arksey and O'Malley (6) and Levac and colleagues (7) and the JBI guidance (4, 5) refer to the process of data extraction in a scoping review as data charting.

§ The process of systematically examining research evidence to assess its validity, results, and relevance before using it to inform a decision. This term is used for items 12 and 19 instead of "risk of bias" (which is more applicable to systematic reviews of interventions) to include and acknowledge the various sources of evidence that may be used in a scoping review (e.g., quantitative and/or qualitative research, expert opinion, and policy document).

From: Tricco AC, Lillie E, Zarin W, O'Brien KK, Colquhoun H, Levac D, et al. PRISMA Extension for Scoping Reviews (PRISMAScR): Checklist and Explanation. Ann Intern Med. 2018;169:467–473. doi: 10.7326/M18-0850.

For peer review only - http://bmjopen.bmj.com/site/about/guidelines.xhtml

BMJ Open

Climate Change-induced Shifts in Food Systems and Diet-related Noncommunicable Diseases in Sub-Saharan Africa: A Scoping Review and a Conceptual Framework

Journal:	*BMJ Open*
Manuscript ID	bmjopen-2023-080241.R2
Article Type:	Original research
Date Submitted by the Author:	10-May-2024
Complete List of Authors:	Tapkigen, Janet; Tampere University, Department of Health Sciences, Faculty of Social Sciences<br>Harding, Seeromanie; King's College London, Department of Population Health Sciences, School of Life Course & Population Sciences<br>Jutta, Pulkki; Tampere University, Department of Health Sciences, Faculty of Social Sciences<br>Atkins, Salla; Tampere University, Department of Health Sciences, Faculty of Social Sciences; Karolinska Institute, Department of Global Public Health<br>Koivusalo, Meri; Tampere University, WHO collaborating Centre on Health in All Policies and the Social Determinants of Health, Health Sciences, Faculty of Social Sciences
**Primary Subject Heading**:	Public health
Secondary Subject Heading:	Global health
Keywords:	EPIDEMIOLOGY, PUBLIC HEALTH, Climate Change

SCHOLARONE™ Manuscripts

I, the Submitting Author has the right to grant and does grant on behalf of all authors of the Work (as defined in the below author licence), an exclusive licence and/or a non-exclusive licence for contributions from authors who are: i) UK Crown employees; ii) where BMJ has agreed a CC-BY licence shall apply, and/or iii) in accordance with the terms applicable for US Federal Government officers or employees acting as part of their official duties; on a worldwide, perpetual, irrevocable, royalty-free basis to BMJ Publishing Group Ltd ("BMJ") its licensees and where the relevant Journal is co-owned by BMJ to the co-owners of the Journal, to publish the Work in this journal and any other BMJ products and to exploit all rights, as set out in our licence.

The Submitting Author accepts and understands that any supply made under these terms is made by BMJ to the Submitting Author unless you are acting as an employee on behalf of your employer or a postgraduate student of an affiliated institution which is paying any applicable article publishing charge ("APC") for Open Access articles. Where the Submitting Author wishes to make the Work available on an Open Access basis (and intends to pay the relevant APC), the terms of reuse of such Open Access shall be governed by a Creative Commons licence – details of these licences and which Creative Commons licence will apply to this Work are set out in our licence referred to above.

Other than as permitted in any relevant BMJ Author's Self Archiving Policies, I confirm this Work has not been accepted for publication elsewhere, is not being considered for publication elsewhere and does not duplicate material already published. I confirm all authors consent to publication of this Work and authorise the granting of this licence.

For peer review only

Title: Climate Change-induced Shifts in Food Systems and Diet-related Noncommunicable Diseases in Sub-Saharan Africa: A Scoping Review and a Conceptual Framework

Authors

Tapkigen, Janet [1]; Harding, Seeromanie [2]; Pulkki, Jutta [1]; Atkins, Salla [1,3]; Koivusalo, Meri [1,4]

Affiliation

[1]Department of Health Sciences, Faculty of Social Sciences, Tampere University, Tampere, Finland.

[2]Department of Population Health Sciences, School of Life Course & Population Sciences, King's College London, London, United Kingdom.

[3]Department of Global Public Health, Karolinska Institutet, Stockholm, Sweden.

[4]Centre for Health in All Policies and Social Determinants of Health, Tampere University, Finland.

Corresponding Author

Janet Tapkigen, MSc.,
Tampere University,
Faculty of Social Sciences,
Arvo Ylpön Katu 34, 33520, Tampere, Finland.
Email: janet.tapkigen@tuni.fi
Tel : +358 (0) 451378262

Keywords: Climate change, Noncommunicable Disease, One health
Word count: 5953

For peer review only

Abstract

Objectives: To determine the relationship between climate change, food systems, and diet-related noncommunicable diseases (DR-NCDs) in Sub-Saharan Africa (SSA) and propose a conceptual framework for food systems in SSA.

Design: A scoping review

Eligibility criteria: Studies included investigated the relationship between climate change and related systemic risks, food systems, DR-NCDs and its risk factors in SSA. Studies focusing on the association between climate change and DR-NCDs unrelated to food systems, such as social inequalities, were excluded.

Sources of evidence: A comprehensive search was conducted in ProQuest (9 databases), Google Scholar and PubMed in December 2022.

Charting methods: Data extracted from studies included: author, study type, country of study, climate change component, DR-NCD outcomes and risk factors, and impacts of climate change on DR-NCDs. A narrative approach was used to analyse the data. Based on the evidence gathered from SSA, we modified an existing food system conceptual framework.

Results: The search retrieved 19,125 studies, ten of which were included in the review. Most studies used a cross-sectional design (n=8). Four explored the influence of temperature on liver cancer through food storage while four explored the influence of temperature and rainfall on diabetes and obesity through food production. Cross-sectional evidence suggested that temperature is associated with liver cancer and rainfall with diabetes.

Conclusion: The review highlights the vulnerability of SSA's food systems to climate change-induced fluctuations, which in turn affect dietary patterns and DR-NCD outcomes. The evidence is scarce and concentrates mostly on the health effects of temperature through food storage. It proposes a conceptual framework to guide future research addressing climate change and DR-NCDs in SSA.

For peer review only

Strengths and limitations of this study

- This scoping review followed the Joanna Briggs Institute methodology for scoping reviews.
- The methodology and search terms involved an extensive search of literature.
- Existing food system framework conceptually guided the methodology and development of hypotheses.
- Despite efforts to search for grey literature, country-specific documents may have been missed; this study is based on published data and, therefore, is subject to publication bias.
- The studies that met the inclusion criterion were primarily observational and descriptive; thus, while these provided valuable insights for hypotheses, their contribution to evidence regarding causality is limited.

For peer review only

For peer review only - http://bmjopen.bmj.com/site/about/guidelines.xhtml

Introduction

Noncommunicable diseases (NCDs) account for 74% (41 million) of all deaths globally every year (1). The primary causes of NCD-related deaths are cardiovascular diseases (accounting for 17.9 million deaths annually), cancer (9.3 million), chronic respiratory diseases (4.1 million), and diabetes (2.0 million) (1) . Collectively, these diseases contribute to 80% of all NCD-related deaths (1). In Sub-Saharan Africa (SSA), cardiovascular diseases are the leading level 2 causes of NCD-related deaths, accounting for 15.1% of the NCD burden. Diabetes and liver cancer also make significant contributions to the NCD burden in the region, accounting for 4.2% and 0.8% of NCD-related deaths, respectively (2). These three diseases – cardiovascular diseases, liver cancer, and diabetes – are known as diet-related NCDs (DR-NCDs), as they are primarily caused by poor dietary habits, characterised by highly processed foods high in saturated fats, sugars, and salt (3).

To effectively address the burden of DR-NCDs in SSA, it is essential to understand how the food systems function, what their drivers are, and their role in shaping diets. Food systems can be described as interconnected networks comprising the environment, people, processes, infrastructure, and institutions involved in various aspects of food production, processing, transportation, storage, distribution, retailing, and consumption (4). The outcomes of these activities and their governance collectively define the functioning of the food system (4). The interconnected components of food systems, namely the food supply chain, food environments, and consumer behaviour, are illustrated in Figure 1.

The food supply chains encompass all stages from production to waste disposal (5), and they depend on ecological, human, energy, and economic systems to produce and distribute foods (6). On the other hand, food environments encompass the settings where consumers make food-related choices (5), therefore connecting food supply and demand (6). Food environments are divided into personal environments (accessibility, affordability, convenience, desirability) and external environments (food vendors, prices, income levels, knowledge, sociocultural norms, marketing practices, regulations) (7). For certain individuals, these environments are local (involving self-produced or locally sourced foods), while for others, they are global (encompassing foods from regional and international sources) (5).

Food environments influence consumer behaviour (6), which refers to the decisions and actions individuals take when acquiring and consuming food (4). These decisions shape diets in terms of quantity, quality, diversity, safety, and adequacy of food (6). Consequently, these diets exert influence on interconnected systems, including nutrition outcomes that directly impact healthcare systems and the climate impacts of diets that affect ecosystems (6).

SSA is currently facing a food systems challenge related to climate change, poor agricultural practices, food insecurity, and the consequences on DR-NCD risk. Climate change threatens the region's ecosystems, agricultural productivity, and food security, exacerbating existing vulnerabilities. Climate change, driven by human activities, has been widely acknowledged as a significant contributor to the development and exacerbation of DR-NCDs (8). The rising global temperatures, erratic rainfall patterns, and changing patterns of extreme weather events

For peer review only

For peer review only - http://bmjopen.bmj.com/site/about/guidelines.xhtml

significantly influence agricultural and food systems (9). These climatic shifts impact food quality, availability, and affordability (9, 10), potentially altering food access and choices, with implications for nutrition and dietary habits (10). As food systems are undergoing profound transformations driven by population growth, globalisation, technology, urbanisation, trade liberalisation, migration, policies, competition for essential resources (water, energy, land), and shifts in income distribution, the impacts of climate change compound the effects of these drivers (4). As such, climate change can be seen as amplifying the underlying drivers associated with DR-NCDs (8, 9).

To effectively address the burden of DR-NCDs in SSA amid these climate-induced changes, a food systems approach is necessary (11). Unlike disease-oriented approaches, food systems approach considers the entire food system — from production to consumption (12). Existing interventions for NCDs in SSA have not focused on multisectoral pathways on determinants of health (13, 14). Health promotion activities to prevent NCDs have focused on individual responsibility and influencing purchase patterns more than a systems approach where the type and availability of food itself is considered. Therefore, shifting towards a food systems approach allows for a holistic and comprehensive understanding of the entire food system, recognising the intricate interplay and feedback loops between different risk factors for NCDs (15).

In the context of addressing DR-NCDs, adopting a systems thinking approach acknowledges that one system alone, for example, the healthcare system, cannot solely determine health outcomes (16). Instead, it recognises the significant role interconnected systems (such as the environment, energy, and government sectors) play in shaping environments that impact DR-NCDs (16). However, implementing a food systems approach in SSA comes with challenges resulting from underreporting underlying determinants of DR-NCDs (17) and limited evidence on the specific pathways in which climate change and related systemic risks influence food systems and, in turn, on DR-NCDs in the region.

Existing food system frameworks have explored the broad-scale interactions and pathways in which climate change influences the risks associated with nutrition outcomes. For instance, the High-level Panel of Experts (HLPE) (18) developed a food systems framework in 2017, illustrating the interplay between diverse drivers within food systems, including climate change, and their effects on health and nutrition outcomes. The HLPE, formed in 2009, is a United Nations body responsible for assessing food security and nutrition research (19). HLPE is governed by 15 world-renowned scientists who are experts in food security and nutrition, and the aim is to assess the current state of food security and nutrition and its underlying causes (19).

While the HLPE framework (18) and previous evidence (8, 20) have provided valuable broad-scale perspectives, there is an urgent need to compile, synthesise and contextualise more localised and region-specific research and to develop contextualised frameworks that can guide future studies. We, therefore, used the original HLPE framework (Figure 1) and existing

For peer review only - http://bmjopen.bmj.com/site/about/guidelines.xhtml

literature to hypothesise and synthesise the causal pathways in which climate change can impact DR-NCDs in SSA.

Aims of the review

This review aims to explore and synthesise research originating from SSA on climate change impacts on food systems and DR-NCDs and to examine if we need to complement and amend the HLPE framework to the SSA context.

For peer review only

For peer review only - http://bmjopen.bmj.com/site/about/guidelines.xhtml

Methods

Conceptual framework development

This study used the original HLPE food systems conceptual framework (18) to hypothesise pathways through which climate change affects food systems and, in turn, DR-NCDs in SSA. The framework is presented in Figure 1 and includes four food supply chain domains: production systems, storage and distribution, processing and packaging, and retail and markets. These supply chains are henceforth referred to as pathways in this review.

In this review, climate change-related systemic risks include any impact of climate change events on other sectors or systems that subsequently influence food systems.

Based on the HLPE framework (18) and knowledge drawn from scientific literature (21-26), we hypothesised pathways in which climate change may affect DR-NCDs and its risk factors (i.e., obesity, overweight) in SSA. These pathways include:

1) The food production pathway. Regarding the *food production pathway,* we hypothesise that the effects of climate change could reduce crop production and yields, consequently reducing food availability. Reduction in food availability could increase food prices, influencing food affordability and, in turn, impact health outcomes (22).

2) The food storage pathway. Under the *food storage pathway,* we propose that reliance on stored crops vulnerable to the impacts of extreme rainfall events can have adverse effects on both the quantity and quality of food. Wet conditions can create a conducive environment for the growth of infectious pathogens. These pathogens, affecting crops during harvesting and storage, could lead to the production of carcinogenic aflatoxins produced by certain moulds, which have been associated with hepatocellular carcinoma, a primary contributor to liver cancer (26).

3) The food distribution and market pathway, and **4) the food processing and packaging pathway**. Within the contexts of *food distribution and market pathways* and *food processing and packaging pathways*, we hypothesise that extreme weather events, such as floods, might disrupt distribution networks, infrastructure, and trade channels, resulting in food losses – especially perishable goods (21, 25). These disruptions can impact the affordability and diversity of food items, impacting food and nutrition security. When comparing fresh perishable foods to processed alternatives, processed foods tend to withstand environmental impacts more effectively, making them a preferable choice within climate variability, albeit with potential trade-offs concerning nutrition value and health implications (21, 25). Moreover, decreased food availability can increase food prices, potentially creating a scenario where imported goods become more easily accessible. Often, these imported products lean towards cost-effectiveness and a high degree of processing.

5) The food retail pathway. In the context of the *food retail pathway*, we hypothesise that extreme weather patterns could reduce crop production and disrupt supply chains for food retailers. Reduced food production and disruptions in the supply chains could lead to shortages of certain food items, affecting consumer choices and potentially changing dietary patterns.

For peer review only - http://bmjopen.bmj.com/site/about/guidelines.xhtml

For instance, if fresh produce becomes scarce due to climate-related disruptions in production and distribution, consumers may opt for less healthy processed alternatives.

6) The food consumption pathway. In the *food consumption pathway*, we posit that climate variability may influence consumption patterns by affecting availability, affordability, and acceptability (27). These shifts could influence food quantity, quality, and prices and reduce income (27). These combined effects can have multifaceted implications for nutritional access, availability, and affordability. This can lead to shifts in dietary preferences, often towards more economical yet highly processed food options (23). The health impact of this pathway is likely to be more important in the light of the focus on processed foods in practice.

Methods

Study design

To augment the HLPE framework with the evidence from the region and to build a context-relevant framework (HLPE-SSA), we conducted a scoping review following the methodological guidance by Joanna Briggs Institute (JBI) (28). The method includes: 1) identifying the research question; 2) inclusion criteria; 3) concept; 4) context; 5) types of evidence sources; 6) search strategy; 7) evidence screening and selection; 8) data extraction; 9) data analysis; and 10) presentation of results.

Scoping reviews are helpful when the literature is complex and serve as a tool to provide policymakers with a comprehensive understanding of the nature of the issue and how that concept has been studied within the existing body of literature (28). This review was reported per the Preferred Reporting Items for Systematic Reviews and Meta-analysis (PRISMA) reporting guidelines (29). This study was based on a registered protocol (https://osf.io/9unbd).

Research question

What kind of research-based evidence exists on climate change, food systems and DR-NCDs relationship from SSA?

Inclusion criteria

We included studies that:

(1) Investigated the relationship between climate change and related systemic risks, food systems, and DR-NCDs and their risk factors;

(2) Were available as full texts;

(3) Had an English abstract or title;

(4) Were related to/conducted in SSA;

Studies were excluded if they:

(1) Examined the association between climate change and DR-NCDs through pathways that do not include food systems, such as social inequalities;

(2) Were not available as full texts;

For peer review only - http://bmjopen.bmj.com/site/about/guidelines.xhtml

For peer review only

(3) Were not conducted in or related to SSA;

(4) Were editorials, letters to the editor, or theoretical discussions of potential impacts or planned programmes that have not yet been implemented, theses, newspapers, magazines, conference abstracts, podcasts, radio programmes, and blogs.

Concept

This review primarily focused on investigating the relationship between climate change and interconnected systemic factors (e.g., urbanisation, globalisation, migration, and food prices), on food systems and subsequently on DR-NCDs and its risk factors.

Context

The scope of this review was centred on examining studies carried out specifically in SSA. These countries are listed in Supplementary Table I. Supplementary Table 1 includes the names of SSA countries, population, GDP per capita, and income category, all retrieved from the World Bank website (30).

Search strategy

The pilot search of PubMed enabled the identification of key terms for the search strategy. The search terms were developed guided by JBI's 'Population, Concept and Context' (PCC) framework and tested in an iterative process to ensure relevance. It included three components: (i) climate change*, (ii) SSA countries, (iii) DR-NCDs*, and nutrition outcomes*. The detailed search strategy is available in Appendix 1. The identified search terms were adapted for other databases. No language restriction was applied in the search. The search started from 1995, when the Global Framework for Climate Change was instituted to address the challenges posed by global warming (31, 32). We included studies published in peer-reviewed journals between 1995 and 2022.

Grey literature search strategy: The search strategy for grey literature was adapted for each data source. The search terms are presented in Appendix 2. Grey literature published from 1995 were considered.

Types of evidence sources

Searches were conducted in ProQuest-9 databases (ProQuest Central, Health and Medical Collection, Environment Science Database, Public Health Collection, Research Library: Health and Medicine, Middle East and Africa Database, Social Science Database, Social Science Premium Collection, and Agriculture Science Database) and PubMed in December 2022. Reference list of relevant citations was further manually searched to identify any articles of interest.

Grey literature data sources: The peer-reviewed searches were supplemented by Google Scholar search in July 2023 involving the first 20 pages. Subsequently, in February 2024, another search was conducted in Google Scholar expanding the search to the first 80 pages as suggested by Haddaway et al. (33) to gain further reach for grey literature. Published and unpublished reports, policy documents and relevant materials from government departments,

For peer review only - http://bmjopen.bmj.com/site/about/guidelines.xhtml

259 agencies and other organisations were also searched for in google searches, and targeted websites including International Food Policy Research Institute (IFPRI), Consultative Group on International Agricultural Research (CGIAR), World Health Organization (WHO), Food and Agriculture Organization (FAO), World Food Programme (WFP) and World Bank in July 2023. Additionally, a sample of seven government websites in SSA were searched for in February 2024. These seven countries (Kenya, Malawi, Nigeria, Zambia, Mauritius, South Africa, and Botswana) were chosen based on recently reported climate change related disasters (34) and to gain coverage from each income group: lower middle-income, low-income, upper middle-income and high income (35). We searched the publications tab of the Ministry of Health website, Ministry of Agriculture and Environment website, Medical Research Council website, and the national research website.

270 **Evidence screening and selection**

271 Following the search, all identified citations were collated and uploaded into Mendeley, where duplicates were removed. The studies were then uploaded to Rayyan.ai (36) for the title and abstract screening. Two authors, JT and MK independently screened the titles and abstracts. Full texts of relevant studies were assessed for inclusion independently by two reviewers (JT and MK). Disagreements were resolved through discussion.

276 The search process identified 19,125 studies after removing 1,221 duplicates. Among these, 7,286 studies were peer-reviewed articles, and 11,239 were grey literature. We excluded 18,428 records based on irrelevant titles and abstracts, leaving us with 106 studies for which we retrieved the full texts for detailed assessment. Of these, ten studies met the eligibility criteria and were included in this review. No studies from the grey literature met our inclusion criteria. The search process is depicted in Figure 2.

282 **Data extraction**

283 JT extracted quantitative and qualitative data in Excel using an extraction form developed for the present study (Appendix 3). The data included the following characteristics: author, study type, country of study, climate change component discussed, DR-NCD outcomes and risk factors, and impacts of climate change on DR-NCDs.

287 **Data analysis**

288 A narrative synthesis (37) approach was applied in this review and presented in tables where appropriate. This involved integrating quantitative and qualitative studies through collation, analysis, synthesis, and presentation (37).

291 **Patient or public involvement**

292 None.

For peer review only - http://bmjopen.bmj.com/site/about/guidelines.xhtml

Results

The results are presented under three main sections: 1) characteristics of included studies; 2) results on the influence of climate change on DR-NCDs; and 3) a conceptual framework for food systems and DR-NCDs in SSA. The results are presented according to the identified food system pathways.

Characteristics of included studies

Ten studies met our inclusion criteria (38-47), including eight quantitative (38, 40-44, 46, 47) and two qualitative studies (39) (45). The majority of studies utilised cross-sectional design (n=8) (38, 40-44, 46, 47). The sample size ranged from 28 to 40,300.

Seven studies were published in the 2020s (38-42, 44, 45), two in the 2010s (43, 47) and one in the 1990s (46). Three studies were conducted in Kenya (39, 40, 47), two in Ghana (42, 45), with one study each in Cameroon (43), Malawi (44), Sudan (46), Tanzania (41), and Zimbabwe (38), as shown in Table 1.

Among the studies included, four studies (38, 42, 43, 47) used primary data collection methods to estimate DR-NCD outcomes and its risk factors. Three studies (40, 41, 44) drew upon data from national health sources to recruit participants. All three studies (40, 41, 44), utilising secondary data, relied on original survey data to measure and estimate health outcomes.

Three studies (38, 42, 47) used AF and FM levels from grain and milk samples to quantify dietary exposure and estimate the risk of liver cancer. Meanwhile, five studies (40, 41, 43, 44, 46) used human samples to assess dietary exposure and DR-NCD risk. Notably, two studies (39, 45) took an alternate approach, looking at participants' perception to illustrate health risks associated with climate change.

Table 1: Overview of included studies

Author	Year	Country	Study design	Aspect of climate change	DR-NCDs and risk factors
Omer (46)	1998	Sudan	Cross-sectional survey	Temperature (related to increased humidity)	Liver cancer
Lontchi-Yimagou (43)	2016	Cameroon	Retrospective study	Temperature and rainfall	Diabetes
Sirma (47)	2019	Kenya	Cross-sectional survey	Rainfall, droughts, temperature	Liver cancer
Odonkor & Sallar (45)	2020	Ghana	Descriptive qualitative	Global warming	Heart disease
Ngwira (44)	2020	Malawi	Cross-sectional analysis	Rainfall	Overweight
Kasomo (40)	2021	Kenya	Cross-sectional analysis	Temperature and rainfall	Overweight, obesity
Akello (38)	2021	Zimbabwe	Cross-sectional analysis	Drought, long dry spells (related to humidity)	Liver cancer
Kimanya (41)	2021	Tanzania	Cross-sectional survey	Temperature (related to increased humidity)	Liver cancer
Andersen (39)	2021	Kenya	Qualitative	Climate change	Diabetes, hypertension, heart attack, and obesity
Kortei (42)	2022	Ghana	Cross-sectional survey	Temperature (related to increased humidity)	Liver cancer

The most common climate variable investigated was temperature (related to increased humidity), reported in 5 out of 10 studies (38, 41, 42, 46, 47). In this review, if a study examined the association between temperature and subsequent rise in humidity levels (above 14%

For peer review only - http://bmjopen.bmj.com/site/about/guidelines.xhtml

moisture content), we classified the climate variable as temperature (related to increased humidity). Four studies investigated DR-NCD outcomes related to variation in rainfall (40, 43, 44, 47), and temperature (40, 41, 43, 47).

Five cross-sectional studies measured (41, 46) and estimated (38, 42, 47) liver cancer rates. Two studies looked at the relationship between climate events and diabetes (39, 43). Three studies examined overweight (40, 44) or obesity (40), two heart disease (39, 43), and one hypertension (39).

Food system pathways through which climate change influences nutrition outcomes and DR-NCDs in SSA

Table 2 shows the results on how climate change influences DR-NCDs. The table is organised based on the food system pathways identified from the theoretical literature and presented below. From the six pathways hypothesised, we found evidence to four.

Food production and retail pathway

Related to food production and retail pathways, four studies examined the relationships between precipitation, temperature, and various health outcomes, including obesity (40), overweight (40, 44), heart disease (45), and DR-NCDs in general (39).

Diabetes, hypertension, heart attack, cancer, overweight, and obesity risk: Two studies (39, 40), indicated a reduction in food production due to variable rainfall and temperature. Findings showed that as annual precipitation and temperature increase due to climate change, so does the likelihood of women being overweight and obese. This was attributed to global warming, which potentially led to water scarcity in Kenya, reduced availability of nutritious crops, and increased reliance on non-nutritious foods (40). In addition, due to the rising temperatures, rainfall patterns have been unpredictable, causing droughts and famines and thus prompting food insecurity and changes in dietary patterns (40). Another study attributed the occurrence of diabetes, hypertension, heart attack, cancer, and obesity to harsh weather patterns, which disrupted agricultural production, resulting in higher costs of food in urban areas due to reliance on imported produce (39). Additionally, climate change-driven reliance on chemically treated food production raised concerns about food quality (39).

Overweight risk: Contrary to other studies, one study suggested that rainfall variability could increase food production, potentially contributing to overweight. The author argued that high rainfall is often associated with better yields fostering overeating behaviours, which may enhance the risk of overweight (44).

Heart disease risk: The association of global warming with food production and heart disease was assessed in Ghana. Respondents perceived that heart disease was a result of global warming (45). As this was based on opinions, the link between climate change and heart diseases was unclear. While climate change implications to heart diseases could be mediated, for example, by heatwaves (48), our focus was on those impacts mediated via the wider food systems to DR-NCDs in SSA and we could not find sufficient evidence to include influence on heart disease to the HLPE-SSA adapted framework.

Food storage pathway

For peer review only - http://bmjopen.bmj.com/site/about/guidelines.xhtml

Related to food storage pathway, five studies (38, 41, 42, 46, 47) examined how temperature (related to increased humidity) and rainfall correlate with liver cancer risks through food storage practices. Aflatoxin (AF) contamination was reported in maize (38, 41), small grains (finger millet, pearl millet, sorghum) (38), milk (47), peanuts (46), and *burkina* — a millet-based fermented milk beverage (42).

Liver cancer risk: In Zimbabwe, mycotoxins as a result of increased rainfall, heat and drought were associated with liver cancer (38). The high temperatures were related to humid conditions in West Sudan, which favoured aflatoxin growth in peanuts, in a region reported with high liver cancer incidences (46). A study conducted in four agroecological zones in Kenya found that aflatoxin exposure from milk was associated with minimal incidence of liver cancer [(47)]. Consumption of small grains (38, 41, 46) and milk consumption posed low liver cancer risks (47), while maize (38) and *burkina* (42) consumption presented high risks. Peanuts also showed an increased risk of liver cancer, particularly in humid conditions (46).

Food consumption pathway

Diabetes risk: One study investigated the impact of rainfall and temperature on food consumption and its correlation with diabetes (43). This study revealed that the highest precipitation level coincided with the peak number of newly diagnosed diabetes cases. At the same time, the hospital admission rates for diabetic patients were slightly higher during the rainy season. The authors attributed this phenomenon to nutritional variability. During the long rainy season, physical activity reduces, and people tend to consume high-fat and high-calorie food while awaiting the harvest. Consequently, increased caloric intake could lead to hyperglycemia and the onset of diabetes symptoms (43).

Grey literature findings

Majority of the reports from the grey literature lacked specificity of region and focus. Among the few reports that explored the relationship between climate change, food systems and DR-NCDs, they did not provide country-specific results (18) or did not explicitly describe which health outcome (49) was being addressed. Although most reports mentioned how climate change impacted food security and malnutrition, the studies did not mention which type of malnutrition (undernutrition or DR-NCDs) it was focusing on (49). Thus, they were not included in this review. We explored a sample of governments but could not find relevant studies to include in this review.

For peer review only - http://bmjopen.bmj.com/site/about/guidelines.xhtml

391 **Table 2: Description of studies included in the review by food system pathway.**

Author/ year	Country	Study design	Method of analysis	Study objectives	Sample size	Climate change component	Outcome	Key findings
**Food production pathway**								
Andersen 2021 (39)	Kenya	Qualitative	Descriptive Statistics	To explore knowledge and perspectives on climate change and health-related issues, with a particular focus on noncommunicable diseases	28	Climate change	Diabetes, hypertension, heart attack, cancer, and obesity	Climate change-induced food insecurity and rising food prices were considered as prominent challenges.
Kasomo 2021 (40)	Kenya	Cross-sectional analysis	Inferential Statistics	To quantify spatial variations and estimate the effect of climatic and environmental factors on under- and over-nutrition among women in Kenya	40,300	Temperature and precipitation	Obesity and overweight	Higher precipitation levels were associated with an increased likelihood of women being overweight and obese.
Ngwira 2020 (44)	Malawi	Cross-sectional analysis	Multivariate probit model	To investigate the effects of climate and location using the multivariate model of malnutrition	5,149	Rainfall	Overweight	A positive correlation was observed between rainfall and overweight individuals.
Odonkor & Sallar 2020 (45)	Ghana	Descriptive cross-sectional design	Inferential Statistics	To investigate public knowledge of global warming and its effects on human health	1,130	Global warming	Heart disease	Rising food prices impact the affordability and availability of food in urban areas.
**Food storage pathway**								
Akello 2021 (38)	Zimbabwe	Cross-sectional analysis	Inferential Statistics	To assess frequencies of mycotoxin-producing fungi in maize, sorghum, pearl millet, and finger millet cropped and traded in Zimbabwe	NI[1]	Drought, long dry spells,	Liver cancer	Consumption of aflatoxin-contaminated maize stored for 4-6 months increased liver cancer rates.

[1] NI-Not indicated

For peer review only - http://bmjopen.bmj.com/site/about/guidelines.xhtml

Kimanya 2021 (41)	Tanzania	Cross-sectional analysis	Inferential Statistics	To estimate the population risk of aflatoxin-induced liver cancer	NI[1]	Temperature	Liver cancer	Population risk for aflatoxin-induced liver cancer nationwide was estimated to be 2.95 cases per 100,000 people.
Kortei 2022 (42)	Ghana	Cross-sectional survey	NI	To evaluate AFM_1 levels and cancer risks associated with *burkina* (a millet-based fermented milk product)	150	Temperature	Liver cancer	Consumption of *burkina* showed varied risk levels of liver cancer.
Omer 1998 (46)	Sudan	Cross-sectional survey	Inferential Statistics	To investigate whether aflatoxin contamination of peanut products may contribute to the incidence of hepatocellular carcinoma (HCC) in Sudan	54	Temperature	Liver cancer	Aflatoxin contamination of peanuts has a significant association with liver cancer.
Sirma 2019 (47)	Kenya	Cross-sectional survey	Inferential Statistics	To assess the risk of liver cancer posed by AFM_1 in milk, assuming 10-fold lower carcinogenicity than AFB_1	NI[1]	Rainfall, droughts, temperature	Liver cancer	Aflatoxin exposure from milk contributed a small percentage to liver cancer incidence rates.
**Food consumption pathway**								
Lontchi-Yimagou 2016 (43)	Cameroon	Retrospective study	Inferential Statistics	To investigate the relationship between diabetes hospitalisation rates and climate variations in Yaoundé	818	Temperature and precipitation	Diabetes	Diabetes admissions were higher during rainy seasons due to nutritional variability.

For peer review only - http://bmjopen.bmj.com/site/about/guidelines.xhtml

Conceptual framework of food systems for DR-NCDs and nutrition with evidence drawn from the local context

Figure 3 presents an amended food systems conceptual framework, named here HLPE-SSA, for understanding the pathways and mechanisms in which climate change impacts on food systems and DR-NCDs in SSA. HLPE-SSA is amended from the HLPE framework and is based on the evidence and results discussed above. While both the HLPE and HLPE-SSA show the pathways in which climate change is linked to food systems and DR-NCD outcomes, HLPE-SSA differs slightly from HLPE. *First* is that HLPE-SSA provides a region-specific analysis of how climate change impacts food systems and DR-NCDs. Due to limited evidence from SSA, we expect the HLPE-SSA framework to change with further data from other areas and new research, for example, with respect to heart disease. *Second*, while HLPE shows more generalised linkages between climate change and nutrition outcomes, HLPE-SSA provides a more in-depth synthesis, specifying the mechanisms by which climate change affects food systems and nutrition. For instance, HLPE-SSA shows how temperature can disrupt agricultural production, reducing food availability and subsequent impacts on the risk of diabetes. *Third*, HLPE-SSA proposes a potential pathway—the food consumption pathway—which is not present in the HLPE framework. This pathway acknowledges that diets and unhealthy consumption practices could also be mediated by other factors beyond food production, such as mobility, marketing, retail, and trade policies.

The evidence from HLPE-SSA aligns with four (production, storage, retail, and consumption) of the six hypothesised pathways.

Regarding the ***food production pathway***, HLPE-SSA confirms our hypothesis. Variations in temperature and precipitation influence food production. Consequently, these changes impact on the quality and quantity of food, which were associated with the risk of diabetes, hypertension, heart attack, obesity, and overweight.

For the ***food storage pathway***, HLPE-SSA confirms our hypothesis regarding the effect of long dry spells, drought and fluctuations in rainfall and temperature on food storage and, in turn, liver cancer in SSA. The impact was more pronounced in *poor storage and harvesting conditions* of grains, whereas unfavourable weather conditions, such as high temperatures and rainfall, increased the risk. These weather conditions impacted humidity levels (above 14% moisture content) increasing aflatoxin growth in grains and milk. Aflatoxin exposure in these grains and milk was linked to the risk of liver cancer.

Regarding the ***food retail pathway***, HLPE-SSA suggests that variations in temperature and rainfall patterns affected trade and markets, disrupting transportation, and increasing food prices. This, in turn, increased the cost of food, thereby impacting its affordability and convenience. These changes in the food systems were associated with an increased risk of diabetes, hypertension, heart attack, obesity, and overweight.

Regarding the ***food consumption pathway***, HLPE-SSA supported our hypothesis that temperature and rainfall patterns alter eating patterns and physical activity during the long rains. This resulted in reduced food quality and nutrient density, which was associated with high diabetes risk.

For peer review only - http://bmjopen.bmj.com/site/about/guidelines.xhtml

434 Lastly, HLPE-SSA framework highlights research gaps related to the *food distribution, market* and *food processing and packaging pathways*. Although we hypothesised that extreme weather events might disrupt the distribution and processing of food in SSA —and consequently on DR-NCDs. To the best of our knowledge, no studies investigated how climatic impacts can influence DR-NCDs through these two pathways.

For peer review only

For peer review only - http://bmjopen.bmj.com/site/about/guidelines.xhtml

Discussion

This scoping review focused on studies on the SSA context to better understand the relevance of local context to food systems and DR-NCDs. It indicates very limited research focus and calls for further research on food systems and DR-NCDs in SSA. Despite this, findings from our review supported our hypotheses that climate change influences food systems through food production, food storage, food retail, and food consumption pathways. These changes in the food chains compromise food quality, safety, quantity, and nutrient density. These factors contribute to a shift towards less nutritious and more processed foods associated with developing DR-NCDs.

Food storage pathway focuses on how climate-induced changes in rainfall and precipitation affect food harvest and storage conditions, leading to the growth of Aflatoxin B_1 and Aflatoxin M_1 (AFM_1) – a type of mycotoxin produced by fungi *Aspergillus flavus* and *Aspergillus parasiticus* – present in grains, maize, nuts, peanuts, flour, and milk (50). The review's findings are consistent with other research conducted in Bangladesh, which reported a significant proportion of liver cancer cases attributed to dietary aflatoxin exposure from maize and other crops in warm and humid conditions (51). Notably, based on the findings of this review, the risk of aflatoxin contamination and its associated liver cancer risk was found to present a lower risk in milk and other grains. This observation is in line with studies by Saha Turna et al. conducted in Bangladesh (52) and globally (51), where aflatoxin exposure in milk and grains such as wheat and rice were not associated with liver cancer. Several factors are identified as influencing aflatoxin contamination, including storage conditions, duration, and type of crop storage structure. Aflatoxins are prone to develop during food production in dry and humid conditions, whereas wet and humid conditions during harvesting and storage create conducive conditions for the growth of aflatoxins (53).

Related to *food production pathway,* evidence from Cameroon, Kenya, and Malawi showed how climate variables correlate with diabetes, heart disease, and obesity. Seasonal changes in rainfall were found to influence physical activity, eating habits and dietary choices, and in turn diabetes and obesity in the region. The review's findings align with other studies that have linked disruptions in agricultural production resulting from extreme weather events with diabetes and obesity (54). During rainy seasons and extreme weather events, agricultural production activities are disrupted, leading to food shortages (42). During food shortages, individuals with diabetes may not consume sufficient fruit and vegetables, and compensate with increased energy-dense, high-fat/salt foods, worsening their condition (42). Another plausible reason could be that people would be more likely to seek care during the rainy season as they are not busy with farm activities.

The increased access to and consumption of processed and unhealthy foods may contribute to the burden of DR-NCDs. *Food consumption pathway* is supported by a review (27) highlighting that extreme weather events are likely to become more frequent determinants of food purchase and consumption. These events can have a dual impact, either by restricting consumers' access to food or by shaping their food preferences based on the availability of certain product (27).

For peer review only - http://bmjopen.bmj.com/site/about/guidelines.xhtml

Evidence from SSA supported four of the hypothesised pathways. Studies in this review did not support the *food distribution, market* and *processing and packaging* pathways. This could be attributed to the limited evidence and studies included in the review and hence could not support all the pathways.

In summary, the HLPE-SSA framework is based on very limited research, but it provides a structured approach to understanding the complex interactions between climate change and related systemic risk, food systems, and DR-NCDs in SSA. It is important that our amendments to the HLPE framework are considered as the first exploratory findings calling for further research from SSA in the area for more comprehensive context-based understanding.

Strengths

This review exhibits several notable strengths.

First, the HLPE framework has been extensively used by other researchers (55, 56), which strengthens the methodology of this review.

Second, this review used an established food systems framework to guide the methodology and research process. This provided a clear structure to clarify pathways on the causal relationship between climate change, food systems, and DR-NCDs.

Third, the review goes beyond existing frameworks by proposing a context-specific framework tailored to SSA. Similar frameworks for food systems exist, but they are extensive in scope. HLPE-SSA, offers an in-depth and specific synthesis of how climate change and certain systemic drivers influence SSA's food systems and contribute to the development of DR-NCDs. This approach enhances the framework's applicability in guiding targeted interventions such as better crop storage, sustainable agriculture, and policies that address the region's specific concerns.

Lastly, the review adhered to a systematic approach, following JBI guidance for conducting scoping reviews. This systematic process ensures the reliability and comprehensiveness of the research process.

Limitations

One key limitation of the review is the possibility of publication bias, as the review focused on international organisations and academic publications. Relevant studies published in country-based databases as well as local languages, might have been missed. However, our search of a sample of government websites did not indicate that we missed substantial body of literature. In addition, even though we did not restrict our search to English, most articles retrieved from our search were in English and thus this might have biased the results.

Additionally, the nature of the studies included in the review – mainly observational and descriptive – limits the establishment of definitive causal relationships between climate change, food systems, and DR-NCDs. Although they offered insights and generated hypotheses of the potential linkages, they may not provide conclusive evidence on causality. Further research, including natural experiments, longitudinal, and time series analysis studies, would be necessary to establish more causal links. This research would also need to focus on the

For peer review only - http://bmjopen.bmj.com/site/about/guidelines.xhtml

proportional role of different pathways of food systems in relation to DR-NCDs in changing societies, dietary, and consumption practices in SSA.

Some studies, mainly qualitative studies, looked at the linkages between climate change and DR-NCDs without showing how they influence each other. Therefore, we had to assemble the scattered evidence from these studies to form a comprehendible picture of these linkages. However, these linkages were not included in the HLPE-SSA framework.

Furthermore, this review did not include studies exploring the potential effects of floods and famine on DR-NCDs. Floods can impact food security by destroying crops and sometimes leading to famine. Floods also destroy the socioeconomic activities linked to food production, which could impact food accessibility, affordability, and utilization. On the other hand, empirical studies have shown that famine exposure in the first 1000 days has been associated with higher risk of diabetes in adulthood (57, 58) (foetal famine), which represents another area that could be relevant to the overall understanding of climate change impacts on DR-NCDs in SSA. Including such evidence in future research could contribute to a more comprehensive analysis of the factors influencing DR-NCDs in the region.

Conclusion

In conclusion, this review suggests climate change can significantly affect food systems, nutrition, and DR-NCDs in SSA. It highlights the association between aflatoxin-contaminated food and liver cancer in multiple countries. It also underscores how seasonal variations in rainfall can influence physical activity and dietary choices, potentially exacerbating diabetes. Furthermore, extreme weather events are identified as influential factors shaping food purchase and consumption patterns, potentially limiting access to nutritious foods and influencing food preferences.

Our work on the HLPE-SSA suggests that while wider conceptual frameworks such as HLPE work as a general reference, it is important for research and practice to case light also to regional adjustments of more global frameworks. This review further highlights the key areas for further work such as the mechanism in which climate change influence heart diseases and the gaps related the *food distribution, market* and *processing and packaging* pathways.

Ethics approval

Ethics approval was not required as the study used publicly available documents.

For peer review only - http://bmjopen.bmj.com/site/about/guidelines.xhtml

References

1. World Health Organization. Noncommunicable diseases: Key facts. 2022.

2. Gouda HN, Charlson F, Sorsdahl K, Ahmadzada S, Ferrari AJ, Erskine H, et al. Burden of non-communicable diseases in sub-Saharan Africa, 1990–2017: results from the Global Burden of Disease Study 2017. The Lancet Global Health. 2019;7(10):e1375-e87.

3. World Health Organization. Sustainable Healthy Diets – Guiding Principles. Rome: World Health Organization; 2019

4. Pingault N, Caron P, Kalafatic C, Allahoury A, Fresco LO, Kennedy E, et al. Nutrition and food systems. A report by the High Level Panel of Experts on Food Security and Nutrition of the Committee on World Food Security. 2017.

5. Fanzo J, Davis C. Global food systems, diets, and nutrition : linking science, economics, and policy. Springer Nature. 2021.

6. HLPE. Food security and nutrition: building a global narrative towards 2030. Report by the High Level Panel of Experts on Food Security and Nutrition of the Committee on World Food Security, Rome. 2020.

7. Turner C, Aggarwal A, Walls H, Herforth A, Drewnowski A, Coates J, et al. Concepts and critical perspectives for food environment research: a global framework with implications for action in low-and middle-income countries. Global food security. 2018;18:93-101.

8. Frumkin H, Haines A. Global Environmental Change and Noncommunicable Disease Risks. Annual Review of Public Health. 2019;40:261-82.

9. Pachauri RK, Allen MR, Barros VR, Broome J, Cramer W, Christ R, et al. Climate change 2014: synthesis report. Contribution of Working Groups I, II and III to the fifth assessment report of the Intergovernmental Panel on Climate Change: Ipcc; 2014.

10. Fanzo J, Davis C, McLaren R, Choufani J. The effect of climate change across food systems: Implications for nutrition outcomes. Global Food Security. 2018;18:12-9.

11. Trudinger H, Crimp S, Friedman RS. Food systems in the face of climate change: reviewing the state of research in South Pacific Islands. Regional Environmental Change. 2023;23(1):45-.

12. Stefan DC, Elzawawy AM, Khaled HM, Ntaganda F, Asiimwe A, Addai BW, et al. Developing cancer control plans in Africa: examples from five countries. The lancet oncology. 2013;14(4):e189-e95.

13. Hunter-Adams J, Battersby J. Health care providers' perspectives of diet-related non-communicable disease in South Africa. BMC Public Health. 2020;20(1).

14. Kassa MD, Grace JM. Noncommunicable diseases prevention policies and their implementation in Africa: a systematic review. Public Health Reviews. 2022;42:1604310.

15. Hawkes C, Parsons K. Tackling Food Systems Challenges: The Role of Food Policy. London, United Kingdom. https://doi. org/10.2307/j. ctvr695rp; 2019.

16. World Health Organization. Systems thinking for noncommunicable disease prevention policy: guidance to bring systems approaches into practice. World Health Organization. Regional Office for Europe; 2022.

17. Dalal S, Beunza JJ, Volmink J, Adebamowo C, Bajunirwe F, Njelekela M, et al. Non-communicable diseases in sub-Saharan Africa: what we know now. International journal of epidemiology. 2011;40(4):885-901.

18. HLPE. Nutrition and food systems. A report by the High Level Panel of Experts on Food Security and Nutrition of the Committee on World Food Security, Rome. 2017.

19. High Level Panel of Experts. About the HLPE-FSN [Available from: https://www.fao.org/cfs/cfs-hlpe/about/mission/en.

For peer review only - http://bmjopen.bmj.com/site/about/guidelines.xhtml

20. Swinburn BA, Kraak VI, Allender S, Atkins VJ, Baker PI, Bogard JR, et al. The global syndemic of obesity, undernutrition, and climate change: the Lancet Commission report. The lancet. 2019;393(10173):791-846.

21. Devereux S. The impact of droughts and floods on food security and policy options to alleviate negative effects. Agricultural Economics. 2007;37:47-58.

22. Fanzo J, Miachon L. Harnessing the connectivity of climate change, food systems and diets: Taking action to improve human and planetary health. Anthropocene. 2023;42:100381-.

23. Scott-Villiers P, Chisholm N, Wanjiku Kelbert A, Hossain N. Precarious lives: work, food and care after the global food crisis. 2016.

24. Springmann M, Mason-D'Croz D, Robinson S, Garnett T, Godfray HCJ, Gollin D, et al. Global and regional health effects of future food production under climate change: a modelling study. The Lancet. 2016;387(10031):1937-46.

25. Thornton PK, Ericksen PJ, Herrero M, Challinor AJ. Climate variability and vulnerability to climate change: a review. Global change biology. 2014;20(11):3313-28.

26. World Health Organization (International Agency for Research on cancer). IARC Monographs on the evaluation of carcinogenic risks to Humans. Some Traditional Herbal Medicines, Some Mycotoxins, Naphthalene and Styrene IARC Monographs on the Evaluation of Carcinogenic Risks to Humans2002.

27. Davis KF, Downs S, Gephart JA. Towards food supply chain resilience to environmental shocks. Nature Food. 2021;2(1):54-65.

28. Peters MDJ, Marnie C, Tricco AC, Pollock D, Munn Z, Alexander L, et al. Updated methodological guidance for the conduct of scoping reviews. JBI Evidence Synthesis. 2020;18(10).

29. Page MJ, McKenzie JE, Bossuyt PM, Boutron I, Hoffmann TC, Mulrow CD, et al. The PRISMA 2020 statement: an updated guideline for reporting systematic reviews. Systematic Reviews. 2021;10(1):1-11.

30. World Bank. Data [Available from: https://data.worldbank.org/region/sub-saharan-africa.

31. L. A G. United Nations Framework Convention on Climate Change. Environ Health Perspect. 2000;108:A353-A.

32. Race to Zero. From 1800 to COP26: A history of key climate conversations. 2021.

33. Haddaway NR, Collins AM, Coughlin D, Kirk S. The Role of Google Scholar in Evidence Reviews and Its Applicability to Grey Literature Searching. PLOS ONE. 2015;10(9):e0138237.

34. Reliefweb. All disasters. Alert/Ongoing [Available from: https://reliefweb.int/disasters?view=ongoing.

35. World Bank. World Bank Country and Lending Groups 2024 [Available from: https://datahelpdesk.worldbank.org/knowledgebase/articles/906519-world-bank-country-and-lending-groups#:~:text=For%20the%20current%202024%20fiscal,those%20with%20a%20GNI%20per.

36. Ouzzani M, Hammady H, Fedorowicz Z, Elmagarmid A. Rayyan—a web and mobile app for systematic reviews. Systematic reviews. 2016;5:1-10.

37. Noyes J, Booth A, Moore G, Flemming K, Tunçalp Ö, Shakibazadeh E. Synthesising quantitative and qualitative evidence to inform guidelines on complex interventions: clarifying the purposes, designs and outlining some methods. BMJ global health. 2019;4(Suppl 1):e000893-e.

For peer review only - http://bmjopen.bmj.com/site/about/guidelines.xhtml

38. Akello J, Ortega-Beltran A, Katati B, Atehnkeng J, Augusto J, Mwila CM, et al. Prevalence of Aflatoxin- and Fumonisin-Producing Fungi Associated with Cereal Crops Grown in Zimbabwe and Their Associated Risks in a Climate Change Scenario. Foods 2021, Vol 10, Page 287. 2021;10(2):287-.

39. Greibe Andersen J, Karekezi C, Ali Z, Yonga G, Kallestrup P, Kraef C. Perspectives of local community leaders, health care workers, volunteers, policy makers and academia on climate change related health risks in Mukuru informal settlement in Nairobi, Kenya—a qualitative study. International Journal of Environmental Research and Public Health. 2021;18(22):12241-.

40. Kasomo JM, Gayawan E. Spatial location, temperature and rainfall diversity affect the double burden of malnutrition among women in Kenya. SSM - Population Health. 2021;16:100939-.

41. Kimanya ME, Michael NR, Emmanuel M, Chibundu NE, Candida PS, Yun YG. Estimating the risk of aflatoxin-induced liver cancer in Tanzania based on biomarker data. PLoS ONE. 2021;16(3).

42. Kortei NK, Annan T, Boakye AA, Essuman EK, Tettey CO, Kyei-Baffour V. Aflatoxin M1 exposure in a fermented millet-based milk beverage 'brukina'and its cancer risk characterization in Greater Accra, Ghana. Scientific Reports. 2022;12(1):12562-.

43. Lontchi-Yimagou E, Tsalefac M, Tapinmene LMT, Noubiap JJN, Balti EV, Nguewa JL, et al. Seasonality in diabetes in Yaounde, Cameroon: a relation with precipitation and temperature. BMC public health. 2016;16(1).

44. Ngwira A. Climate and location as determinants of childhood stunting, wasting, and overweight: an application of semiparametric multivariate probit model. Nutrition. 2020;70:100010-.

45. Odonkor ST, Sallar AM. An Assessment of Public Knowledge and Potential Health Impacts of Global Warming in Ghana. BioMed Research International. 2020;2020.

46. Omer RE, Bakker MI, van P, A P Hoogenboom RL, G Polman TH, Alink GM, et al. Aflatoxin and liver cancer in Sudan. NUTRITION AND CANCER. 1998;32(3):174-80.

47. Sirma AJ, Makita K, Randolph DG, Senerwa D, Lindahl JF. Aflatoxin Exposure from Milk in Rural Kenya and the Contribution to the Risk of Liver Cancer. Toxins 2019, Vol 11, Page 469. 2019;11(8):469-.

48. Liu J, Varghese BM, Hansen A, Zhang Y, Driscoll T, Morgan G, et al. Heat exposure and cardiovascular health outcomes: a systematic review and meta-analysis. The Lancet Planetary Health. 2022;6(6):e484-e95.

49. AfDB. Economic Brief - Health in Africa Over the Next 50 Years. 2013.

50. Bbosa GS, Kitya D, Lubega A, Ogwal-Okeng J, Anokbonggo WW, Kyegombe DB. Review of the biological and health effects of aflatoxins on body organs and body systems. Aflatoxins-recent advances and future prospects. 2013;12:239-65.

51. Saha Turna N, Wu F. Risk assessment of aflatoxin-related liver cancer in Bangladesh. Food Additives & Contaminants: Part A. 2019;36(2):320-6.

52. Saha Turna N, Havelaar A, Adesogan A, Wu F. Aflatoxin M1 in milk does not contribute substantially to global liver cancer incidence. The American Journal of Clinical Nutrition. 2022;115(6):1473-80.

53. IARC. Monographs on the evaluation of carcinogenic risks to humans: Overall evaluations of carcinogenicity: An updating of IARC Monographs. Lyon, France. International Agency for Research on Cancer. 1987:1-403.

54. Zilbermint M. Diabetes and climate change. Journal of Community Hospital Internal Medicine Perspectives. 2020;10(5):409-12.

For peer review only - http://bmjopen.bmj.com/site/about/guidelines.xhtml

55. Harris J, Tan W, Raneri JE, Schreinemachers P, Herforth A. Vegetables for healthy diets in low-and middle-income countries: a scoping review of the food systems literature. Food and Nutrition Bulletin. 2022;43(2):232-48.
56. Storhaug IG, Lane C, Moore N, Engelbert M, Sparling TM, Franich A, et al. Making the most of existing research: an evidence gap map of the effects of food systems interventions in low-income and middle-income countries. BMJ open. 2022;12(6):e055062.
57. Kraemer K, Cordaro J, Fanzo J, Gibney M, Kennedy E, Labrique A, et al. Diet and non-communicable diseases: an urgent need for new paradigms. Good nutrition: Perspectives for the 21st century: Karger Publishers; 2016. p. 105-18.
58. Nyirenda MJ. Non-communicable diseases in sub-Saharan Africa: understanding the drivers of the epidemic to inform intervention strategies. International health. 2016;8(3):157-8.

For peer review only

Funding statement

JT was supported by the EDUFI Fellowship (Finnish National Agency for Education). SH was funded by the Medical Research Council grant number MR/S009035/1, MR/N015959/1, MR/S003444/1, MR/Y009983/1, MR/X009777/ and MR/X003078/1. MK, JP, and SA have not declared grant from any funding agency in the public, commercial or not-for-profit sectors.

Risk and quality assessment

As this is a scoping review, no risk or quality assessment was conducted.

Data availability statement

There are not any more data available. All data relevant to this review, have been included in the article or uploaded online supplemental information.

Contributions

JT, SH, JP, and MK conceptualised the article. JT and MK conducted the screening of studies. JT did data extraction. JT, SA, JP, SH, and MK contributed to the study methodology. JT, SH, MK, SA, and JP contributed to writing – review and editing. JT contributed to writing – original draft.

Conflict of interest

The authors declare no conflict of interest.

Figures

Figure 1: "HLPE. 2017. Nutrition and food systems. A report by the High Level Panel of Experts on Food Security and Nutrition of the Committee on World Food Security, Rome." This is the original HLPE framework. The food supply chains in this framework are henceforth referred to as referred to as pathways in this review.

Figure 2: PRISMA Flow Diagram: This diagram shows the systematic process we followed to include articles by our search framework.

Figure 3: Food systems conceptual framework for Sub-Saharan Africa. HLPE-SSA. Figure adapted from HLPE. 2017. Nutrition and food systems. A report by the High Level Panel of Experts on Food Security and Nutrition of the Committee on World Food Security, Rome." Arrows show the expected causal effect.

For peer review only - http://bmjopen.bmj.com/site/about/guidelines.xhtml

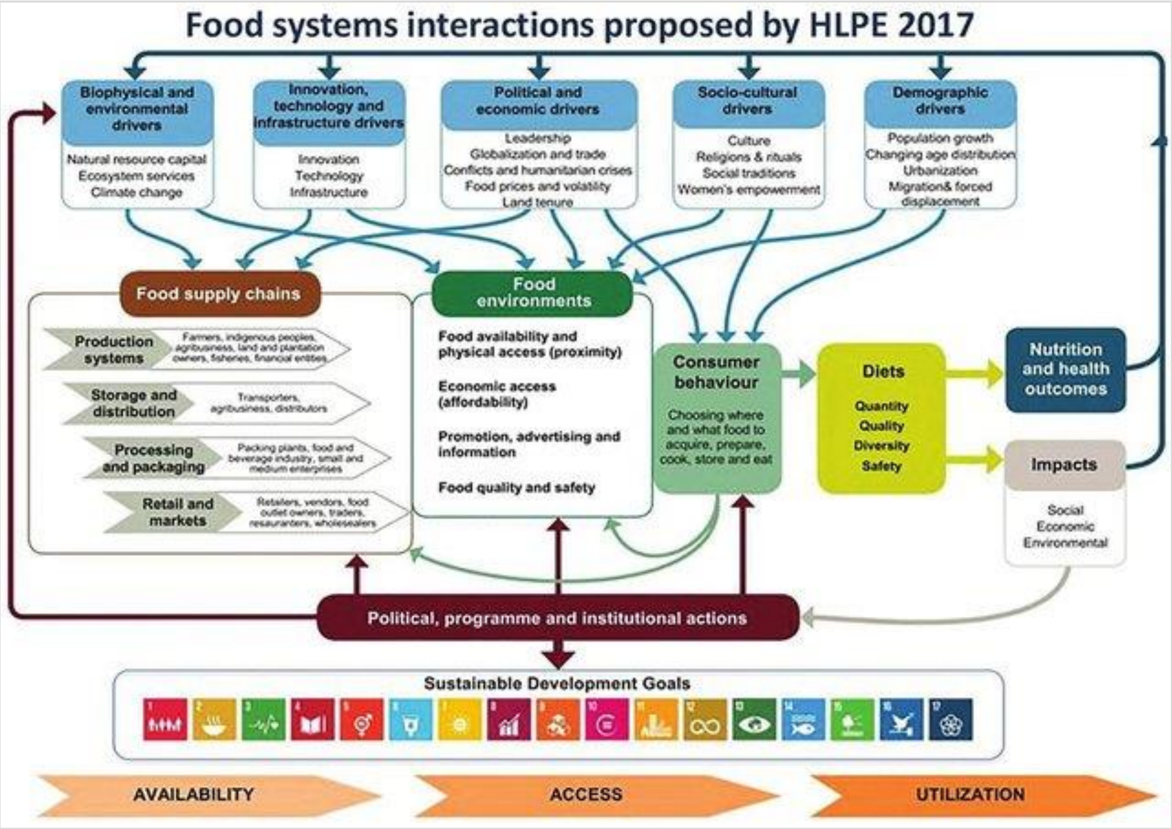
Food systems interactions proposed by HLPE 2017
Biophysical and environmental drivers
Natural resource capital
Ecosystem services
Climate change
Innovation, technology and infrastructure drivers
Innovation
Technology
Infrastructure
Political and economic drivers
Leadership
Globalization and trade
Conflicts and humanitarian crises
Food prices and volatility
Land tenure
Socio-cultural drivers
Culture
Religions & rituals
Social traditions
Women's empowerment
Demographic drivers
Population growth
Changing age distribution
Urbanization
Migration& forced displacement
Food supply chains
Production systems
Farmers, indigenous peoples, agribusiness, land and plantation owners, fisheries, financial entities
Storage and distribution
Transporters, agribusiness, distributors
Processing and packaging
Packing plants, food and beverage industry, small and medium enterprises
Retail and markets
Retailers, vendors, food outlet owners, traders, resauranters, wholesealers
Food environments
Food availability and physical access (proximity)
Economic access (affordability)
Promotion, advertising and information
Food quality and safety
Consumer behaviour
Choosing where and what food to acquire, prepare, cook, store and eat
Diets
Quantity
Quality
Diversity
Safety
Nutrition and health outcomes
Impacts
Social
Economic
Environmental
Political, programme and institutional actions
Sustainable Development Goals
AVAILABILITY
ACCESS
UTILIZATION

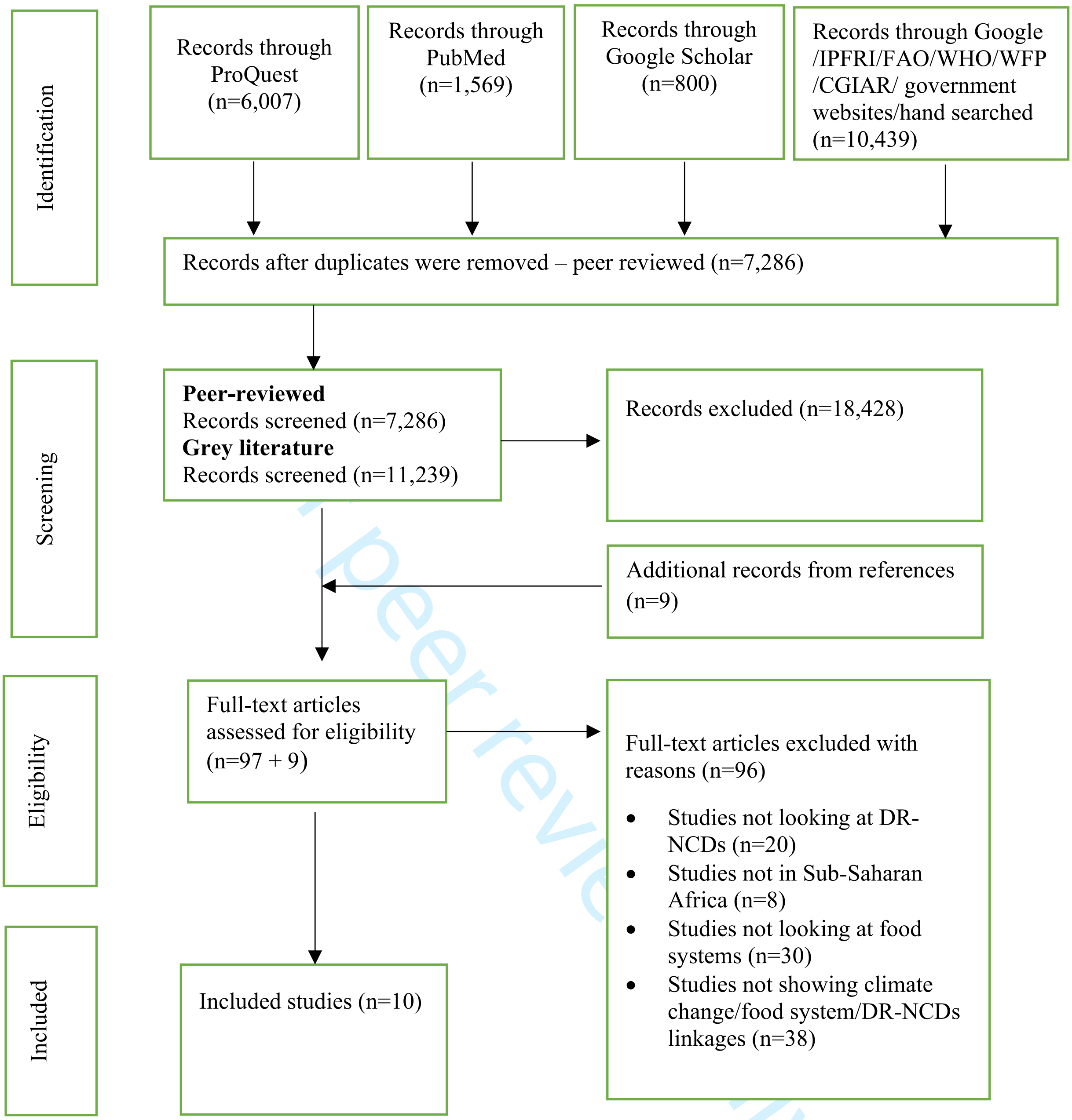
Identification
Records through ProQuest (n=6,007)
Records through PubMed (n=1,569)
Records through Google Scholar (n=800)
Records through Google /IPFRI/FAO/WHO/WFP /CGIAR/ government websites/hand searched (n=10,439)
Records after duplicates were removed – peer reviewed (n=7,286)
Screening
Peer-reviewed
Records screened (n=7,286)
Grey literature
Records screened (n=11,239)
Records excluded (n=18,428)
Additional records from references (n=9)
Eligibility
Full-text articles assessed for eligibility (n=97 + 9)
Full-text articles excluded with reasons (n=96)
Studies not looking at DR-NCDs (n=20)
Studies not in Sub-Saharan Africa (n=8)
Studies not looking at food systems (n=30)
Studies not showing climate change/food system/DR-NCDs linkages (n=38)
Included
Included studies (n=10)

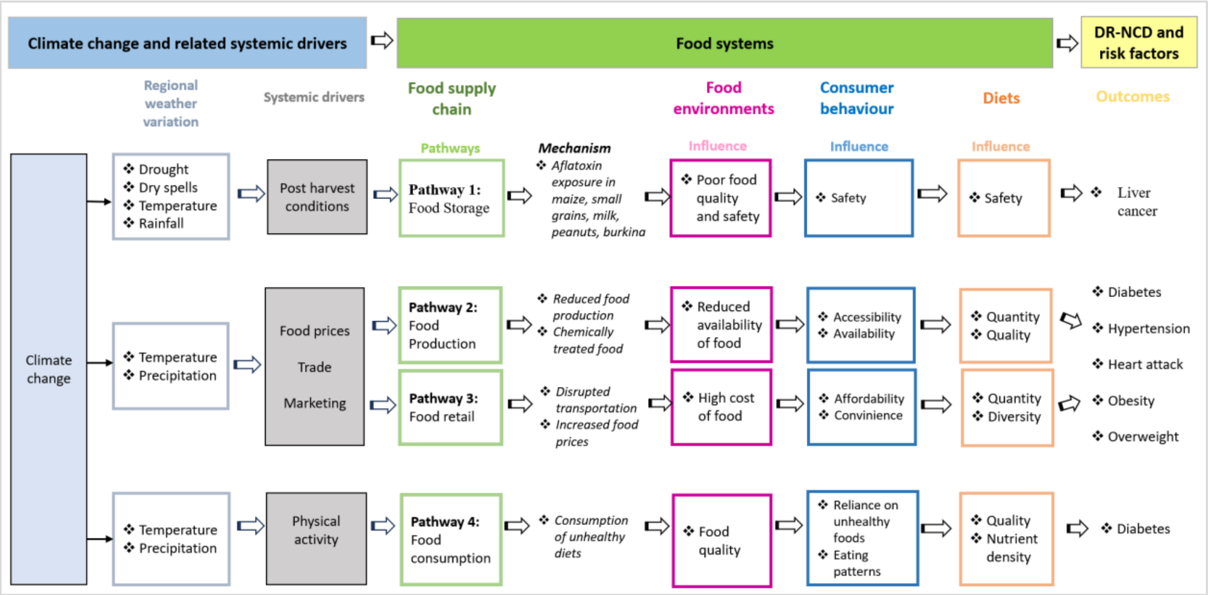

For peer review only

Appendix 1

Search terms-Academic databases

"Sub-Saharan Africa" OR Angola OR Benin OR Botswana OR Burkina Faso OR Burundi OR "Cabo Verde" OR Cameroon OR "Central African Republic" OR Chad OR Comoros OR "Democratic Republic of Congo" OR "Republic of Congo" OR "Cote d'Ivoire" OR "Equatorial Guinea" OR Eritrea OR Eswatini OR Swaziland OR Ethiopia OR Gabon OR Gambia OR Ghana OR Guinea OR Guinea-Bissau OR Kenya OR Lesotho OR Liberia OR Madagascar OR Malawi OR Mali OR Mauritania OR Mauritius OR Mozambique OR Namibia OR Niger OR Nigeria OR Rwanda OR "Sao Tome and Principe" OR Senegal OR Seychelles OR "Sierra Leone" OR Somalia OR "South Africa" OR "South Sudan" OR Sudan OR Tanzania OR Togo OR Uganda OR Zambia OR Zimbabwe AND "Noncommunicable Diseases"[Mesh] OR "Noncommunicable Disease*" [tw] OR "Non-communicable Diseases" [tw] OR "Non communicable Diseases" [tw] OR "Non-communicable Chronic Diseases" [tw] OR "Non-communicable Chronic Disease*" [tw] OR "Non-infectious Diseases" [tw] OR "Chronic diseases" [tw] OR diabetes*[tw] OR DM* OR T2DM* OR overweight OR Hypertension*[tw] OR "high blood pressure"[tw] OR obesity*[tw] OR cholestrol* [tw] OR cancer*[tw] OR "Chronic respiratory diseases" AND "Climate Change"[Mesh] OR "Climate Changes*" [tw] OR "Climate change*" [tw] OR "climate impacts" OR "greenhouse gas" [tw] OR floods*[tw] OR drought*[tw] OR "heat waves*" [tw] OR greenhouse*[tw] OR "global warming"[tw] OR famine*[tw] OR temperature[tw] OR "extreme weather*"[tw] OR humidity OR precipitation OR "water-related"

For peer review only

Appendix 2

Search terms-Grey literature

Google Scholar and Google

Sub-Saharan Africa AND Noncommunicable Diseases OR Noncommunicable Disease OR Non-communicable Diseases OR Non communicable Diseases OR Non-communicable Chronic Diseases OR Non-communicable Chronic Disease OR Non-infectious Diseases OR Chronic diseases OR diabetes OR DM OR T2DM OR overweight OR Hypertension OR high blood pressure OR obesity OR cholesterol OR cancer OR Chronic respiratory diseases AND Climate Change OR Climate Changes OR Climate change OR climate impacts OR greenhouse gas OR floods OR drought OR heat waves OR greenhouse OR global warming OR famine OR temperature OR extreme weather OR humidity OR precipitation OR water-related

FAO

Climate change OR heat OR famine OR floods AND diabetes OR NCDs OR noncommunicable diseases OR Obesity OR cancer OR cardiovascular diseases AND Sub-Saharan Africa OR Republic of Congo OR Cote d'Ivoire OR Equatorial Guinea OR Eritrea OR Eswatini OR Swaziland OR Ethiopia OR Gabon OR Gambia OR Ghana OR Guinea OR Guinea-Bissau OR Kenya OR Lesotho OR Liberia OR Madagascar OR Malawi OR Mali OR Mauritania OR Mauritius OR Mozambique OR Namibia OR Sub-Saharan Africa OR Angola OR Benin OR Botswana OR Burkina Faso OR Burundi OR Cabo Verde OR Cameroon OR Central African Republic OR Chad OR Comoros OR Democratic Republic of Congo OR Niger OR Nigeria OR Rwanda OR Sao Tome and Principe OR Senegal OR Seychelles OR Sierra Leone OR Somalia OR South Africa OR South Sudan" OR Sudan OR Tanzania OR Togo OR Uganda OR Zambia OR Zimbabwe

WHO, WFP and World Bank

Climate change OR heat OR famine OR floods AND diabetes OR noncommunicable diseases OR NCDs OR Obesity OR cancer OR cardiovascular diseases AND Sub-Saharan

IPFRI, CGIAR

Search terms were not used in IPFRI and CGIAR websites, but hand searches were used to search studies under the publications and tools tab. This is because the search terms were bringing null results.

Government websites

Climate change OR heat OR famine OR floods AND diabetes OR NCDs OR noncommunicable diseases OR obesity OR cancer OR cardiovascular diseases AND Sub-Saharan Africa

No search limits used.

For peer review only

Appendix 3: Extraction sheet

Author	Year	Study type	Aim of the study	Country of study	Climate change components discussed	Food systems pathway	Outcomes	Results on the effects of climate change on the outcomes	Causal or hypothetical association

For peer review only

Supplementary Table 1
List of Sub-Saharan Africa countries

No	Country	Population (World Bank 2022)	GDP per capita (PPP) ($) (World Bank 2022)	World Bank 2023 Income Classification
1	Angola	35,588,987	6,976.0	Lower middle income
2	Benin	13,352.86	4,057.5	Lower middle income
3	Botswana	2,384,246	18,329.8	Upper middle income
4	Burkina Faso	22,673,762	2,549.9	Low income
5	Burundi	12,889,576	836.5	Low income
6	Cabo Verde	593,149	8,716.0	Lower middle income
7	Cameroon	27,914,536	4,398.0	Lower middle income
8	Central African Republic	5,579,144	973.2	Low income
9	Chad	17,723,315	1,668.6	Low income
10	Comoros	836,774	3,833.7	Lower middle income
11	Congo, Democratic Republic	99,010,212	1,337.8	Low income
12	Congo Republic	5,970,424	4,335.	Lower middle income
13	Côte d'ivoire	28,160,542	6,540.5	Lower middle income
14	Equatorial Guinea	1,674,908	17,620.7	Upper middle income
15	Eritrea	3,684,032	1,628.8	Low income
16	Eswatini	1,201,670	10,699.5	Lower middle income
17	Ethiopia	123,379,924	2,812.5	Low income
18	Gabon	2,388,992	16,465.0	Upper middle income
19	The Gambia	2,705,992	2,496.5	Low income
20	Ghana	33,475,870	6,473.1	Lower middle income
21	Guinea	13,859,341	3,188.1	Lower middle income
22	Guinea-Bissau	2,105,566	2,191.2	Low income
23	Kenya	54,027,487	5,765.8	Lower middle income
24	Lesotho	2,305,825	2,646.2	Lower middle income
25	Liberia	5,302,681	1,570.5	Low income
26	Madagascar	29,611,714	1,774.7	Low income
27	Malawi	20,405,317	1,732.6	Low income
28	Mali	22,593,590	2,518.9	Low income
29	Mauritania	4,736,139	6,295.8	Lower middle income
30	Mauritius	1,262,523	26,979.1	Upper middle income
31	Mozambique	32,969,518	1,477.3	Low income
32	Namibia	2,567,012	11,531.3	Upper middle income
33	Niger	26,207,977	1,505.5	Low income
34	Nigeria	218,541,212	5,862.2	Lower middle income
35	Rwanda	13,776,698	2,793.2	Low income
36	Sao Tome and Principe	227,38	4,061.8	Lower middle income
37	Senegal	17,316,449	4,210.4	Lower middle income
38	Seychelles	119,878	29,771.7	High income

For peer review only

39	Sierra Leone	8,605,718	1,930.9	Low income
40	Somalia	17,597,511	1,711.0	Low income
41	South Africa	59,893,885	15,920.4	Upper middle income
42	South Sudan	10,913,164	1,181.9	Low income
43	Sudan	46,874,204	4,217.4	Low income
44	Tanzania	65,497,748	3,099.2	Lower middle income
45	Togo	8,848,699	2,601.8	Low income
46	Uganda	47,249,585	2,693.1	Low income
47	Zambia	20,017,675	3,975.6	Lower middle income
48	Zimbabwe	16,320,537	2,607.9	Lower middle income

Data retrieved from the World Bank website.

For peer review only

Preferred Reporting Items for Systematic reviews and Meta-Analyses extension for Scoping Reviews (PRISMA-ScR) Checklist

SECTION	ITEM	PRISMA-ScR CHECKLIST ITEM	REPORTED ON PAGE #
**TITLE**			
Title	1	Identify the report as a scoping review.	1
**ABSTRACT**			
Structured summary	2	Provide a structured summary that includes (as applicable): background, objectives, eligibility criteria, sources of evidence, charting methods, results, and conclusions that relate to the review questions and objectives.	2
**INTRODUCTION**			
Rationale	3	Describe the rationale for the review in the context of what is already known. Explain why the review questions/objectives lend themselves to a scoping review approach.	4-6
Objectives	4	Provide an explicit statement of the questions and objectives being addressed with reference to their key elements (e.g., population or participants, concepts, and context) or other relevant key elements used to conceptualize the review questions and/or objectives.	6
**METHODS**			
Protocol and registration	5	Indicate whether a review protocol exists; state if and where it can be accessed (e.g., a Web address); and if available, provide registration information, including the registration number.	NA
Eligibility criteria	6	Specify characteristics of the sources of evidence used as eligibility criteria (e.g., years considered, language, and publication status), and provide a rationale.	8-9
Information sources*	7	Describe all information sources in the search (e.g., databases with dates of coverage and contact with authors to identify additional sources), as well as the date the most recent search was executed.	9
Search	8	Present the full electronic search strategy for at least 1 database, including any limits used, such that it could be repeated.	9
Selection of sources of evidence†	9	State the process for selecting sources of evidence (i.e., screening and eligibility) included in the scoping review.	10
Data charting process‡	10	Describe the methods of charting data from the included sources of evidence (e.g., calibrated forms or forms that have been tested by the team before their use, and whether data charting was done independently or in duplicate) and any processes for obtaining and confirming data from investigators.	10
Data items	11	List and define all variables for which data were sought and any assumptions and simplifications made.	9
Critical appraisal of individual sources of evidence§	12	If done, provide a rationale for conducting a critical appraisal of included sources of evidence; describe the methods used and how this information was used in any data synthesis (if appropriate).	NA
Synthesis of results	13	Describe the methods of handling and summarizing the data that were charted.	10

For peer review only

SECTION	ITEM	PRISMA-ScR CHECKLIST ITEM	REPORTED ON PAGE #
**RESULTS**			
Selection of sources of evidence	14	Give numbers of sources of evidence screened, assessed for eligibility, and included in the review, with reasons for exclusions at each stage, ideally using a flow diagram.	10
Characteristics of sources of evidence	15	For each source of evidence, present characteristics for which data were charted and provide the citations.	11
Critical appraisal within sources of evidence	16	If done, present data on critical appraisal of included sources of evidence (see item 12).	NA
Results of individual sources of evidence	17	For each included source of evidence, present the relevant data that were charted that relate to the review questions and objectives.	10
Synthesis of results	18	Summarize and/or present the charting results as they relate to the review questions and objectives.	11-17
**DISCUSSION**			
Summary of evidence	19	Summarize the main results (including an overview of concepts, themes, and types of evidence available), link to the review questions and objectives, and consider the relevance to key groups.	18
Limitations	20	Discuss the limitations of the scoping review process.	19
Conclusions	21	Provide a general interpretation of the results with respect to the review questions and objectives, as well as potential implications and/or next steps.	20
**FUNDING**			
Funding	22	Describe sources of funding for the included sources of evidence, as well as sources of funding for the scoping review. Describe the role of the funders of the scoping review.	25

JBI = Joanna Briggs Institute; PRISMA-ScR = Preferred Reporting Items for Systematic reviews and Meta-Analyses extension for Scoping Reviews.

* Where *sources of evidence* (see second footnote) are compiled from, such as bibliographic databases, social media platforms, and Web sites.

† A more inclusive/heterogeneous term used to account for the different types of evidence or data sources (e.g., quantitative and/or qualitative research, expert opinion, and policy documents) that may be eligible in a scoping review as opposed to only studies. This is not to be confused with *information sources* (see first footnote).

‡ The frameworks by Arksey and O'Malley (6) and Levac and colleagues (7) and the JBI guidance (4, 5) refer to the process of data extraction in a scoping review as data charting.

§ The process of systematically examining research evidence to assess its validity, results, and relevance before using it to inform a decision. This term is used for items 12 and 19 instead of "risk of bias" (which is more applicable to systematic reviews of interventions) to include and acknowledge the various sources of evidence that may be used in a scoping review (e.g., quantitative and/or qualitative research, expert opinion, and policy document).

From: Tricco AC, Lillie E, Zarin W, O'Brien KK, Colquhoun H, Levac D, et al. PRISMA Extension for Scoping Reviews (PRISMAScR): Checklist and Explanation. Ann Intern Med. 2018;169:467–473. doi: 10.7326/M18-0850.

For peer review only - http://bmjopen.bmj.com/site/about/guidelines.xhtml
